# Synthetic Studies to Help Elucidate the Metabolism of the Preclinical Candidate TBAJ-876—A Less Toxic and More Potent Analogue of Bedaquiline

**DOI:** 10.3390/molecules25061423

**Published:** 2020-03-20

**Authors:** Peter J. Choi, Daniel Conole, Hamish S. Sutherland, Adrian Blaser, Amy S.T. Tong, Christopher B. Cooper, Anna M. Upton, Brian D. Palmer, William A. Denny

**Affiliations:** 1 Auckland Cancer Society Research Centre, School of Medical Sciences, University of Auckland, Private Bag 92019, Auckland 1142, New Zealand; conoledaniel@gmail.com (D.C.); h.sutherland@auckland.ac.nz (H.S.S.); a.blaser@auckland.ac.nz (A.B.); amytong0606@gmail.com (A.S.T.T.); b.palmer@auckland.ac.nz (B.D.P.); b.denny@auckland.ac.nz (W.A.D.); 2 Maurice Wilkins Centre, University of Auckland, Private Bag 92019, Auckland 1142, New Zealand; 3 Global Alliance for TB Drug Development, 40 Wall St, New York, NY 10005, USA; christopher.cooper@tballiance.org (C.B.C.); anna.upton@tballiance.org (A.M.U.)

**Keywords:** bedaquiline, TMC207, Sirturo, bedaquiline analogues, TBAJ-876, mycobacterium tuberculosis, tuberculosis, drug development

## Abstract

Bedaquiline is a novel drug approved in 2012 by the FDA for treatment of drug-resistant tuberculosis (TB). Although it shows high efficacy towards drug-resistant forms of TB, its use has been limited by the potential for significant side effects. In particular, bedaquiline is a very lipophilic compound with an associated long terminal half-life and shows potent inhibition of the cardiac potassium hERG channel, resulting in QTc interval prolongation in humans that may result in cardiac arrhythmia. To address these issues, we carried out a drug discovery programme to develop an improved second generation analogue of bedaquiline. From this medicinal chemistry program, a candidate (TBAJ-876) has been selected to undergo further preclinical evaluation. During this evaluation, three major metabolites arising from TBAJ-876 were observed in several preclinical animal models. We report here our synthetic efforts to unequivocally structurally characterize these three metabolites through their independent directed synthesis.

## 1. Introduction

Tuberculosis (TB) is one of the top ten leading causes of death in the world and is the leading cause of death from a single infectious agent. In fact, TB is more lethal than HIV/AIDS globally [1]. It was estimated TB caused 1.5 million deaths in 2018, with 10 million new cases of TB estimated for that year alone. The often late detection of TB [2], coupled with the need to use long and complex multi-drug treatment regimens to treat it, has led to an alarming increase in cases which are resistant to the standard front-line drugs for drug sensitive TB (i.e., rifamicin, isoniazid, pyrazinamide, and ethambutol). In 2017, about 558,000 people developed TB that was resistant to rifampicin, and of these, 82% had multidrug-resistant TB (MDR TB), and this proportion has been rising rapidly [1].

**Figure 1:**
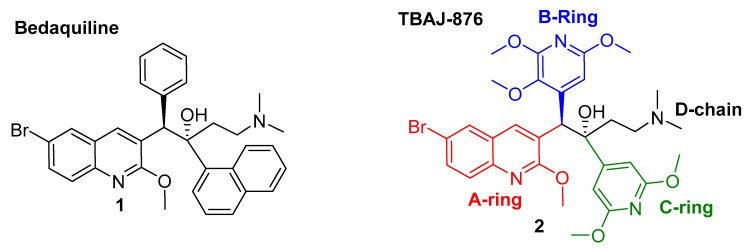
Structure of bedaquiline **1** and preclinical candidate TBAJ-876 **2**.

The discovery and regulatory approval of the novel antitubercular agent bedaquiline **1** (TMC207, Sirturo; Figure 1) has been of great significance within the TB community. It has a unique diarylquinoline (DARQ) structure and a novel mechanism of action (inhibition of the mycobacterial ATP synthase [3]), and has shown significant activity against drug-resistant tuberculosis strains (MDR TB, and XDR TB). When added to standard background therapy used for MDR TB, it demonstrated more rapid bactericidal activity than standard therapy alone [4] and was approved by the FDA for treatment of MDR TB in 2012, making it the first TB drug with a novel mode of action in 40 years. However, bedaquiline does have some significant draw-backs. In a registration trial, bedaquiline showed an increased incidence of death compared to the placebo treatment group, possibly due to several pharmacological factors [5]. It is very lipophilic (measured log P of 7.25), and binds to fatty tissues in the body and has been associated with an increased incidence of phospholipidosis [6]. This lipophilicity also likely contributes to its long terminal half-life of 5–6 months [7]. It inhibits the cardiac potassium hERG channels to cause QTc interval prolongation (resulting in delayed ventricular repolarization). This is a serious consideration for combination regimens with other anti-TB drugs such as the fluoroquinolones or clofazimine, which also exhibit such cardiovascular side effects [8]. Due to such potential toxicities, the WHO advises caution when using bedaquiline, and recommends that strict monitoring procedures be put in place for patients taking bedaquiline [9]. 

With such limitations present with bedaquiline, the identification of new analogues with similar or better anti-bacterial potency but lower clogP and diminished inhibition of hERG channel activity would be of tremendous interest. In light of this, an exploration of the diarylquinolines to identify improved second generation analogues of bedaquiline was initiated [10,11,12,13,14,15]. After extensive drug discovery efforts, TBAJ-876 **2** was selected for preclinical evaluation as a more potent, less lipophilic analogue with lower cardiotoxic potential than bedaqiline **1** (Table 1) [16]. 

**Scheme 1:**
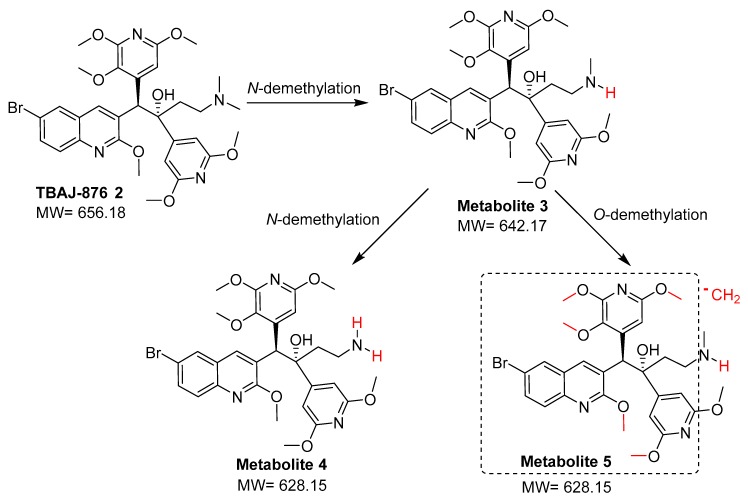
Proposed metabolic pathways of TBAJ-876 **2** in rat plasma.

The metabolic fate of TBAJ-876 **2** was evaluated over the course of several preclinical in vivo studies. In particular, TBAJ-876 **2** was administered orally at 10.0 mg/kg QD to Sprague Dawley rats for 5 days, and plasma samples were obtained at the conclusion of the final dosing period. While the majority of the test article remained intact, several circulating metabolites were identified in pooled rat plasma, and their structures were tentatively assigned by mass spectrometry fragmentation. From this in vivo experiment, three major metabolites were identified and assigned structures **3**–**5** as shown in Scheme 1. There were no species difference between rat and mouse metabolism of TBAJ-876 and rats were selected as they were well represented across various preclinical studies.

Metabolite **3** is conjectured to arise from mono-demethylation in the dimethylamino sidechain (D-chain) of TBAJ-876 **2** while metabolite **4** represents the bis-demethylated analogue. Metabolite **5** had two sites of demethylation, mono-demethylated in the D-chain, together with a second mono-demethylation of one of the other methoxy groups in the molecule. Such metabolites are important factors to consider when conducting drug safety studies for new pharmaceutical drugs. The identification and characterization of major metabolites of TBAJ-876 would help establish their impacts on safety and efficacy of TBAJ-876 for further preclinical studies. Synthetic studies towards these possible metabolites to help elucidate the metabolism of TBAJ-876, and to provide authentic standards for mass spectrometry and for metabolite profiling studies, are outlined below.

## 2. Results and Discussion

### 2.1. Synthesis of Metabolite ***3***

**Scheme 2:**
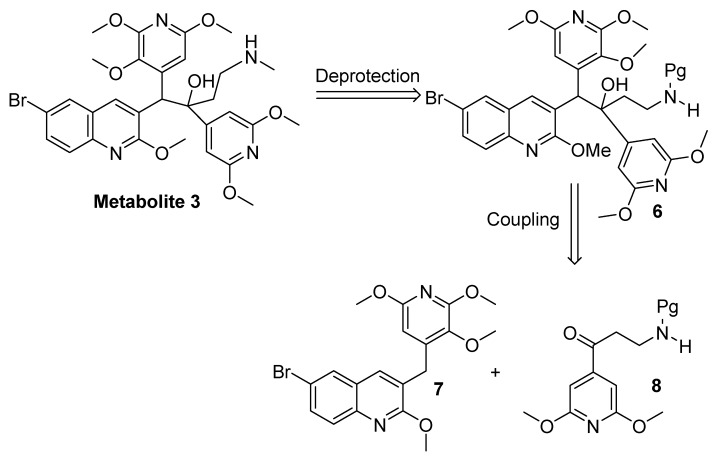
Proposed synthesis of Metabolite **3**.

The chemistry to access metabolite **3** is shown in Scheme 2.

**Scheme 3:**
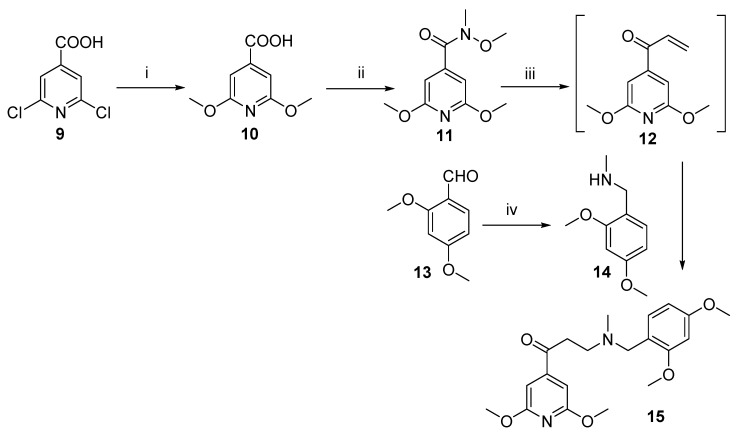
Synthesis of Mannich base **15**. Reagents and conditions: (i) NaOMe, MeOH, 130 °C, 18 h, 87%; (ii) (COCl)_2_, cat. DMF, DCM, then MeNH(OMe).HCl, pyridine, 20 °C, 18 h, 83%; (iii) vinylMgBr, THF, 0 °C then **14**, H_2_O, 68%; (iv) MeNH_2_, NaBH_4_, MeOH, 2 h, 100%.

The A/B unit **7** [14] would be coupled with an appropriately protected Mannich base **8** to give **6**, which, following protecting group removal would yield the desired metabolite **3**. It was imperative that the protecting group for Mannich base **8** be acid labile, as base labile protecting groups would be difficult to remove cleanly. Diarylquinoline analogues of bedaquiline are unstable under basic conditions leading to retro-aldol reactions and reversion to their respective starting units [17]. The protecting group would also need to be stable enough to survive the LDA used in the standard DARQ coupling conditions. After several trials, dimethoxybenzyl (DMB) was chosen as the most appropriate protecting group (other acid labile protecting groups such as BOC and silyl protecting groups had failed to yield Mannich base **8**). Commercially available acid **9** was converted to **10** using sodium methoxide at 130 °C for 18 h (Scheme 3). It was important to form the sodium methoxide in situ using methanol and sodium metal as premade commercial grade reagents gave varying results including incomplete reaction and mono displacement of methoxide. **10** was converted to the Weinreb amide **11** using oxalyl chloride, followed by addition of hydroxylamine hydrochloride salt and pyridine. With **11** in hand, it was added to vinyl magnesium bromide to provide vinyl ketone intermediate **12** in situ. The addition of amine **14** and water gave a 68% yield of the desired protected Mannich base **15**.

**Scheme 4:**
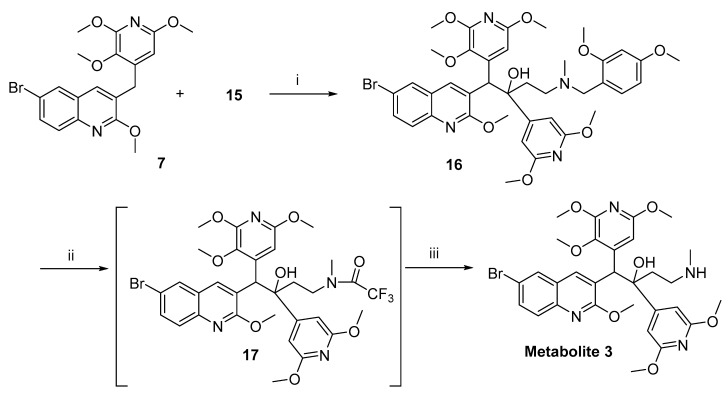
Synthesis of metabolite **3**. Reagents and conditions: (i) LDA, THF, −75 °C, 1.5 h then **15**, −75 °C, 5 h, 69%; (ii) TFAA, DCM, 1 h; (iii) Cs_2_CO_3_, MeOH, −20 °C, 12 h, (49%).

Gratifyingly, Mannich base **15** underwent coupling with A/B unit **7** under standard DARQ coupling conditions [10,11,12] to give coupled product DARQ **16** in 69% yield as a 1:1 mixture of diastereomers (Scheme 4). The direct removal of the DMB protecting group using various acidic conditions (TFA, 2M HCl) was unsuccessful. Instead, we resorted to converting DMB group to trifluoroacetate **17** by reacting **16** with TFAA in DCM and then removing the trifluoroacetate group using a mild base such as cesium carbonate [18] despite our previous experience that this could lead to retro-coupling. Treatment of **17** in MeOH with cesium carbonate at r.t. did indeed cause retro-coupling to yield A/B unit **7** with no signs of desired metabolite **3**. However, after the careful optimisation of the reaction conditions by carrying out the reaction at −20 °C gave the desired metabolite **3** with minimal retro-coupling.

### 2.2. Synthesis of Metabolite ***4***

**Scheme 5:**
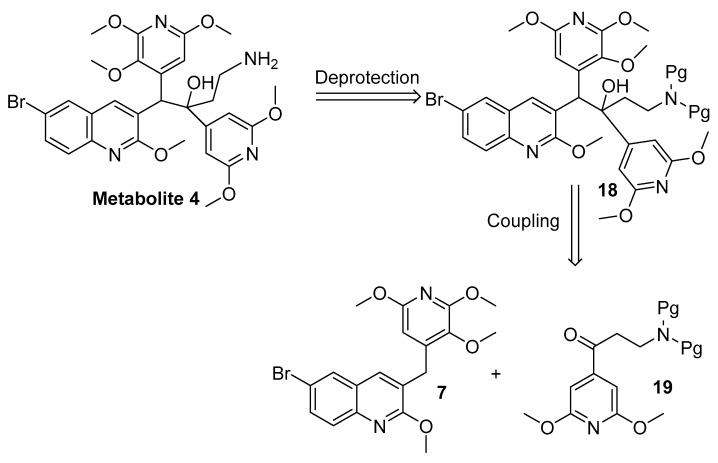
Proposed synthesis of metabolite **4**.

The chemistry to access metabolite **4** is outlined in Scheme 5 below. 

**Scheme 6:**
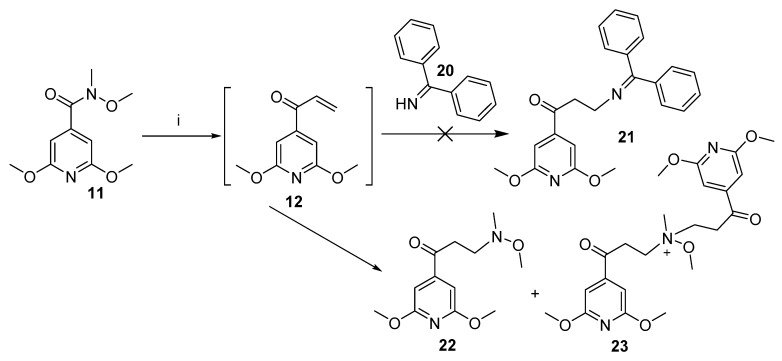
Attempted synthesis of intermediate **21**. Reagents and conditions: (i) vinylMgBr, THF, 0 °C then **20**, H_2_O.

The synthetic plan to access metabolite **4** was similar to that for metabolite **3**, but instead of using mono-protected Mannich base **15**, a suitably bis-protected Mannich base **19** was needed (Scheme 5). The benzophenone imine protected Mannich base **21** was considered to be a suitable protecting group as it could be readily cleaved in acidic conditions and yet also be stable during the coupling reaction conditions. Synthesis began with Weinreb amide **11**, to which was added vinyl magnesium bromide to form vinyl ketone intermediate **12**, which was then added to benzophenone imine **20** followed by addition of water (Scheme 6). However, instead of generating desired ketone **21**, it formed two major by-products **22** and **23**. This could be explained by the cleaved *N*,*O*-dimethylhydroxylamine in the reaction mixture from **11** reacting with vinyl ketone intermediate **12** to yield by-product **22**. **23** is a quaternary salt formed from another equivalent of vinyl ketone **12** being added to by-product **22**. 

**Scheme 7:**
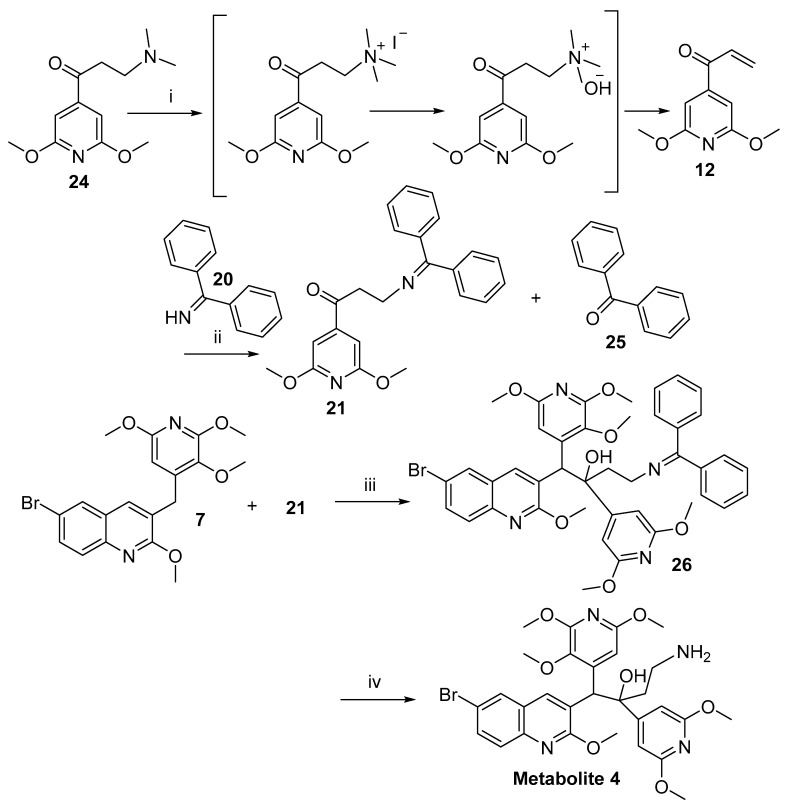
Synthesis of intermediate **21** and metabolite **4**. Reagents and conditions: (i) MeI, DCM, 14 h, 41%; (ii) benzophenone imine **20**, THF, 0.5 h, 68%; (iii) LDA, THF, −75 °C, 1.5 h then **21**, −75 °C, 5 h, 35%; (iv) NH_2_OH-HCl, Na_2_CO_3_, DCM, 48 h, 53%.

This problem was overcome by isolating the vinyl ketone intermediate **12** from the reaction mixture instead of using it in situ, hence avoiding the presence of competing nucleophiles, such as cleaved *N*,*O*-dimethylhydroxylamine. By using a modified reported procedure [19], Mannich base **24** [14] was treated with methyl iodide for 72 h to form quaternary ammonium iodide salt which was washed with water and subsequent elimination liberated pure vinyl compound **12** (Scheme 7). With **12** isolated, it was dissolved in THF, added benzophenone imine **20** and stirred at r.t. for 30 min. After work up and silica chromatography, desired Mannich base **21** was isolated along with ~10% of benzophenone **25** which could be removed during purification on the next step. Several other protecting groups were also investigated, however were unsuccessful in forming bis-BOC protected or bis-DMB protected Mannich base **19**.

Compound **21** was then coupled successfully with A/B unit **7** to yield DARQ **26**. Removal of benzophenone imine protecting group was achieved using hydroxylamine and sodium carbonate in DCM to give metabolite **4** in 53% yield. 

### 2.3. Synthesis of Metabolite ***5***

**Figure 2:**
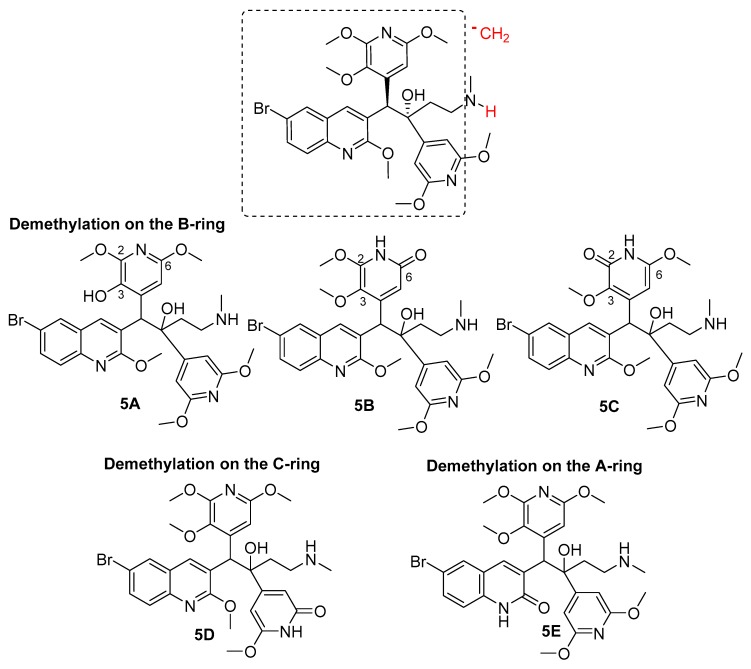
Five possible structures of metabolite **5**.

The exact structure of metabolite **5** was unknown. From mass spectral evidence, it had two sites of demethylation, being mono-demethylated in the D-chain and mono-demethylated somewhere in one of the methoxy groups in the molecule. This leaves five possible sites of demethylation and the possible structures for metabolite **5** are outlined in Figure 2. 

#### 2.3.1. Metabolite **5A**

**Scheme 8:**
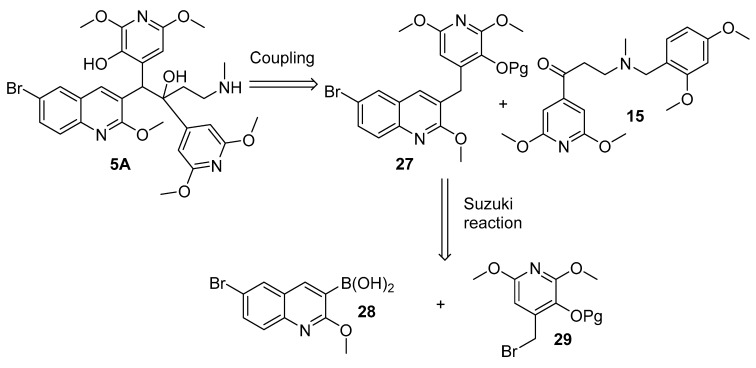
Proposed synthesis of metabolite **5A**.

Metabolite **5A** has a demethylated D-chain with a demethylation at the methoxy group on the 3-position of the pyridine B-ring (Scheme 8). Metabolite **5A** was synthesized from suitably protected A/B unit **27**, which was coupled with the previously synthesized Mannich base **15**.

**Scheme 9:**
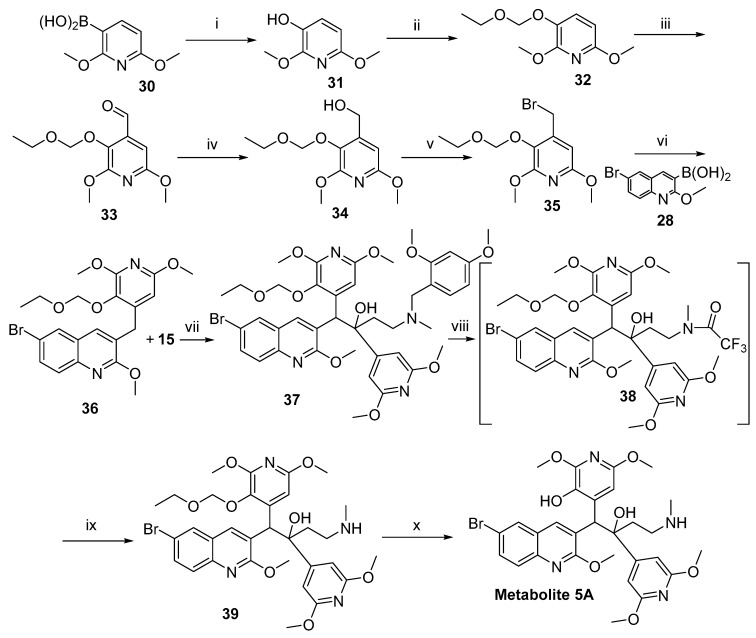
Synthesis of key intermediate **36** and metabolite **5A**. Reagents and conditions: (i) 32% peracetic acid in acetic acid, THF, 2 h, 90%; (ii) NaH (60% in mineral oil), chloromethyl ethyl ether, DMF, 2 h, 93%; (iii) LDA, THF, DMF, 3 h 51%; (iv) NaBH_4_, MeOH, 2 h, 93%; (v) MsCl, DCM, 0.5 h then LiBr, acetone, 100 °C, 99%; (vi) Cs_2_CO_3_, Pd(PPh_3_)_4_, PhMe/DMF, 35 °C (sealed tube), 1 h, 76%; (vii) LDA, THF, −75 °C, 1.5 h then **15**, −75 °C, 5 h, 69%; (viii) TFAA, DCM, 1 h; (ix) Cs_2_CO_3_, MeOH, −20 °C, 72 h, 49%; (x) 1M HCl, dioxane, 24 h, Isomer A: 20%, Isomer B: 27%.

Synthesis of A/B unit **27** began with boronic acid **30** which was converted to alcohol **31** in 90% yield (Scheme 9). Alcohol **31** was protected with an ethoxymethyl ether (EOM) protecting group to yield **32**. Formylation of **32** gave aldehyde **33** which was reduced to alcohol **34** using sodium borohydride in good yields. The conversion of alcohol **34** to a mesylate group which was displaced by lithium bromide furnished bromide **35**. Suzuki reaction between bromide **35** and boronic acid **28** produced desired A/B unit **36**. It was important to carry out the Suzuki reaction at 35 °C as higher temperatures (80 °C) led to degradation of products. With **36** in hand, it was coupled with Mannich base **15** to give **37** as 1:1 mixture of diastereomers in 69% yield. The subsequent removal of the DMB-protecting group using previously established chemistry followed by the removal of the EOM-protecting group using 1M HCl in dioxane yielded desired metabolite **5A**.

#### 2.3.2. Metabolite **5B**

**Scheme 10:**
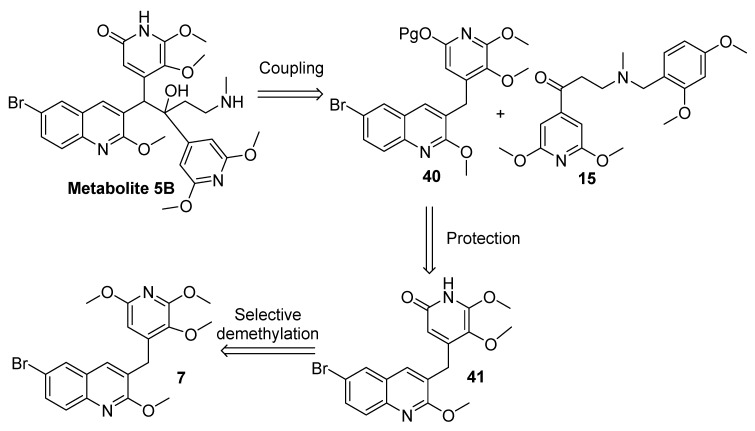
Proposed synthesis of metabolite **5B**.

Metabolite **5B** has a demethylated D-chain with a demethylation at the methoxy group on the 6-position of the pyridine B-ring (Scheme 10). Metabolite **5B** was synthesized from suitably protected A/B unit **40** which was coupled with previously synthesized Mannich base **15**. A selective mono-demethylation of A/B unit **7** was proposed to yield **41** as 6-methoxy group adjacent to the nitrogen of the B-ring in **7** would be activated. Although methoxy group at the 2-position is also activated, steric hindrance from methoxy group at the 3-position would hinder demethylation from occurring.

**Scheme 11:**
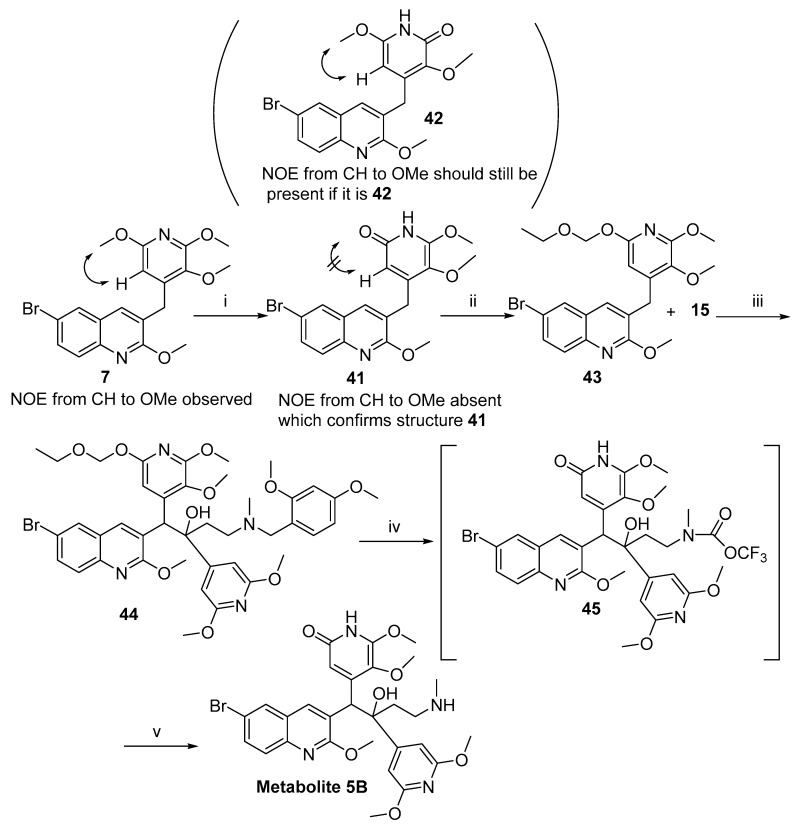
Synthesis of key intermediate **43** and metabolite **5B**. Reagents and conditions: (i) LiCl, pTSA, DMF, 120 °C, 1 h, 90%; (ii) DIPEA, chloromethyl ethyl ether, DCM, 18 h, 49%; (iii) LDA, THF, −75 °C, 1.5 h then **15**, −75 °C, 5 h, 83%; (iv) TFAA, DCM, 1 h; (v) Cs_2_CO_3_, MeOH, −20 °C, 48 h, Isomer A: 19%, Isomer B: 21%.

The selective demethylation of **7** was achieved using lithium chloride and pTSA in DMF [20] (Scheme 11). This reaction condition was a mild alternative which gave a far superior yield (90%) compared to BBr_3_ (15%), a common demethylation reagent. The structure of **41** was confirmed by ^1^H NMR and 2D NOESY experiments. A/B unit **7** shows a clear NOE signal between the 5-H and the adjacent CH_3_ on the 6-methoxy group in the pyridine B-ring while for product **41**, NOE signal clearly disappears, confirming it is structure **41** as this signal would still be present if it was structure **42**. It was pleasing demethylation had occurred selectively at the desired 6-position of the pyridine B-ring with excellent yields. With structure of **41** confirmed, it was dissolved in DCM, added DIPEA and chloromethyl ethyl ether and stirred at r.t. for 18 h to give EOM protected A/B unit **43** in adequate yields. **43** was submitted to our standard coupling reaction conditions with DMB protected Mannich base **15** to yield DARQ **44**. Using similar chemistry as the previous metabolites, treatment of **44** with TFAA led to deprotection of both the EOM and the DMB group to furnish trifluoroacetamide **45**. Subsequent mild basic hydrolysis conditions yielded metabolite **5B** as pure high and low Rf diastereomers. 

#### 2.3.3. Metabolite **5C**

**Scheme 12:**
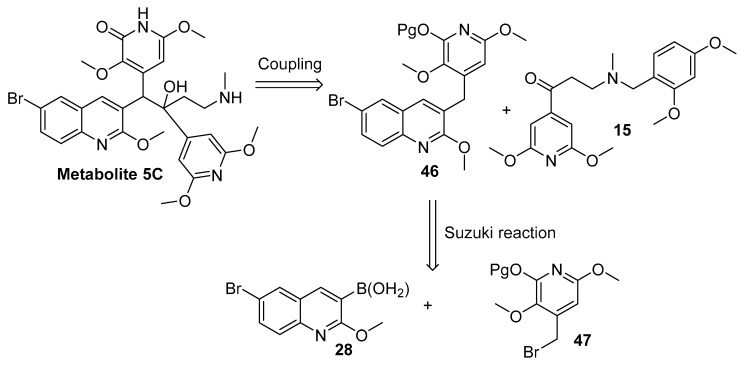
Proposed synthesis of metabolite **5C**.

Metabolite **5C** has a demethylated D side chain with mono demethylation on the methoxy group at the 2 position in the B-ring (Scheme 12). Metabolite **5C** was accessed from a coupling reaction between suitably protected A/B unit **46** with Mannich base **15**. A/B unit **46** could be synthesized from bromide **47** which would undergo a Suzuki reaction with boronic acid **28**.

**Scheme 13:**
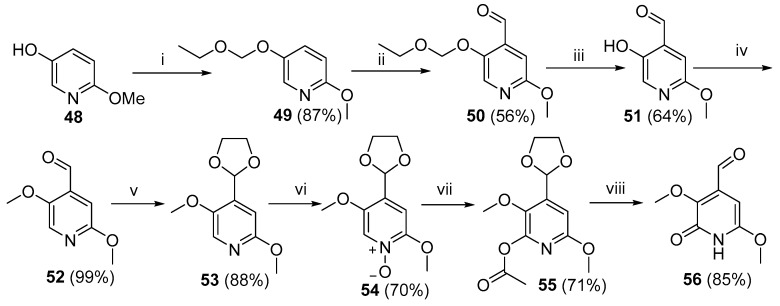
Synthesis of intermediate **56**. Reagents and conditions: (i) NaH, chloromethyl ethyl ether, DMF, 2 h, 87%; (ii) LDA, THF, 0 °C, 3 h then *n*-formylpiperidine, 56%; (iii) 3M HCl, THF, 0 °C, 3 h, 64%; (iv) K_2_CO_3_, MeI, DMF, 0 °C, 3 h, 99%; (v) 1,2-ethanediol, PPTS, PhMe, 0 °C, 4 h, 88%; (vi) *m*-CPBA, DCM, 18 h, 70%; (vii) Ac_2_O, 120 °C, 4 h, 71%; (viii) TFA, CHCl_3_, 18 h, 85%.

The synthesis of suitably protected bromide **47** began with commercially available pyridine **48**, which underwent protection with chloromethyl ethyl ether to yield **49** (Scheme 13). Formylation of **49** with *N*-formylpiperidine gave **50** followed by removal of EOM protecting group and methylation using iodomethane gave aldehyde **52**. Reactive aldehyde functionality needed to be protected before *N*-oxide chemistry could be attempted. Aldehyde **52** was protected with 1,2-ethanediol and pyridinium tosylate to give dioxalane **53** in good yields. With aldehyde protected, oxidation to *N*-oxide using *m*-CPBA gave **54** in 70% yield. Upon refluxing **54** in acetic anhydride, it gave **55** in 71% yield. Dioxalane protecting group and acetate group was removed using trifluoroacetic acid to yield desired aldehyde **56**.

**Scheme 14:**
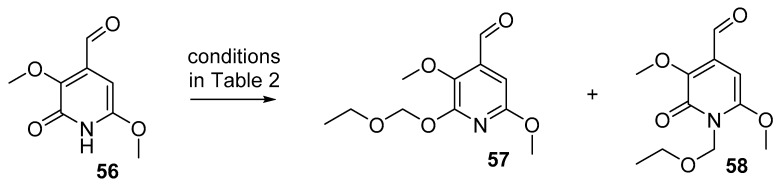
Synthesis of intermediates **57** and **58**.

The next step was EOM protection of **56** (Scheme 14). Using our standard EOM protection procedure, **56** was dissolved in DCM and added DIPEA and chloromethyl ethyl ether. This reaction gave a mixture of EOM-protected products **57** and **58** where EOM alkylation had occurred on both the nitrogen and the oxygen of the pyridone **56**. *N*-alkylation was favoured yielding 4:1 ratio of **58**:**57**. (Table 2, condition 1). Although it did not matter which position the protecting group went as it would be removed in the final step to give the same product, we ideally wanted *O*-protection (**57**) as similar *O*-protected A/B units (**46,** Scheme 12) were known to undergo successful DARQ coupling reactions. 

Interestingly, solvent polarity had a huge influence on the ratio of *O* and *N*-alkylated products being formed. The product formation of *O*-alkylation and *N*-alkylation could be reversed by using a range of solvents with different polarities. Using a non-polar solvent such as benzene (condition 2) promoted more *N*-alkylation **58** over *O*-alkylation **57** (9.5:5) while carrying out the reaction with a polar solvent like DMF promoted more *O*-alkylation **57** over *N*-alkylation **58** (4:1). The addition of silver carbonate to form silver salt of 2-pyridones, which is reported to promote *O*-alkylation under non-polar solvents [21] did not yield any *O*-alkylated product, instead only yielding *N*-alkylated product **58** (conditions 4 and 5).

**Scheme 15:**
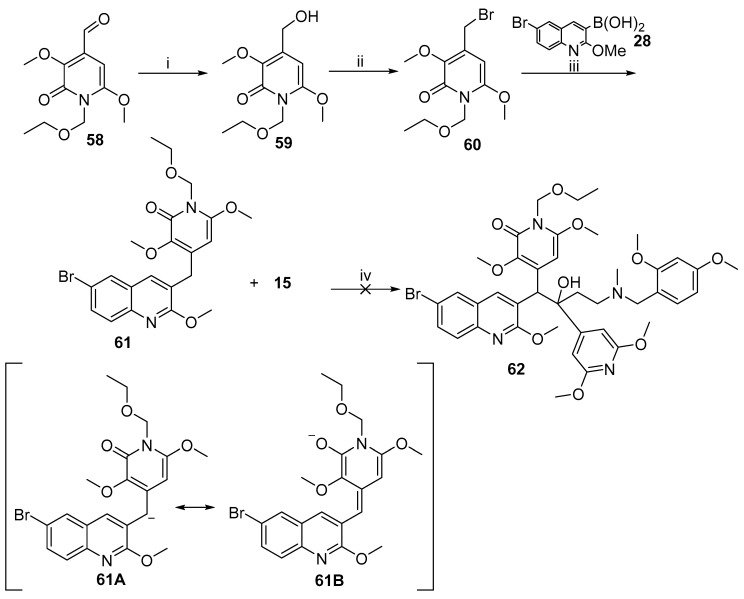
Synthesis of intermediate **61** and attempted coupling reaction with **15**. Reagents and conditions: (i) NaBH_4_, MeOH, 2 h, 98%; (ii) MsCl, DCM, 0.5 h then LiBr, acetone, 100 °C, 93%; (iii) Cs_2_CO_3_, Pd(PPh_3_)_4_, PhMe/DMF, 35 °C (sealed tube), 1 h, 61%; (iv) LDA, THF, −75 °C, 1.5 h then **15**, −75 °C, 5 h.

**Scheme 16:**
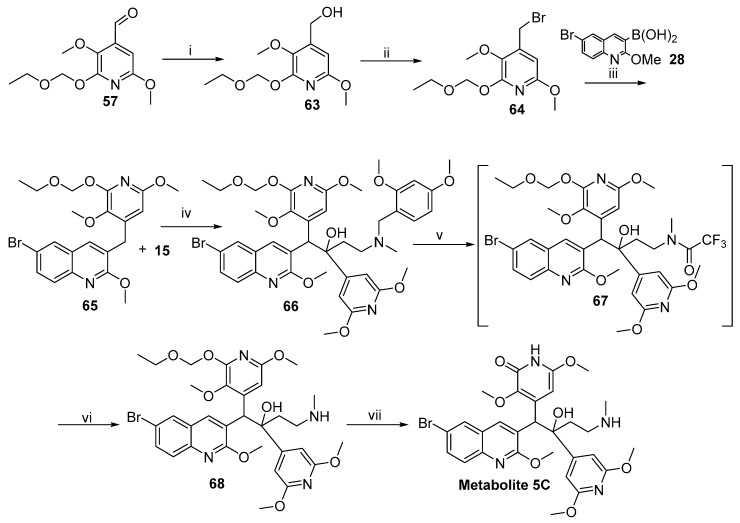
Synthesis of key intermediate **65** and metabolite **5C**. Reagents and conditions: (i) NaBH_4_, MeOH, 2 h, 92%; (ii) MsCl, DCM, 0.25 h then LiBr, acetone, 0 °C, 98%; (iii) Cs_2_CO_3_, Pd(PPh_3_)_4_, PhMe/DMF, 50 °C (sealed tube), 1 h, 81%; (iv) LDA, THF, −75 °C, 1.5 h then **15**, −75 °C, 5 h, 59%; (v) TFAA, DCM, 1 h; (vi) Cs_2_CO_3_, MeOH, −20 °C, 48 h, 67%; (vii) 2M HCl in Et_2_O, 0 °C, 0.5 h, Isomer A: 54%, Isomer B: 23%.

With both **57** and **58** in hand, using similar chemistry as before, they were successfully modified to A/B units **65** and **61** respectively (Scheme 15 and Scheme 16). However only O-alkylated A/B unit **65** underwent a successful coupling reaction with Mannich base **15** to yield **66** (Scheme 16). A/B unit **61** did form the characteristic dark-purple/wine coloured reaction mixture once LDA was added, which suggested the successful formation of anion at the benzylic position of **61** (a common phenomenon in most DARQ coupling reactions). However, it is presumed the benzylic anion formed is stabilized by the changed electronics in the B-ring to prevent reaction with Mannich base **15**. Failed coupling reactions were commonly observed with A/B units with B-rings which stabilized the anion formed at the benzylic position.

Gratifyingly, A/B unit **65** was successfully coupled with **15** to yield DARQ **66**. Following established chemistry as for the previous metabolites, the DMB group deprotection was carried out to give trifluoroacetamide **67** followed by DARQ **68**. Final removal of EOM group was achieved using anhydrous 2M HCl in diethyl ether to give metabolite **5C** as 1:1 mixture of diastereomers (isomer A) and some as pure low Rf diastereomers (isomer B).

**Figure 3:**
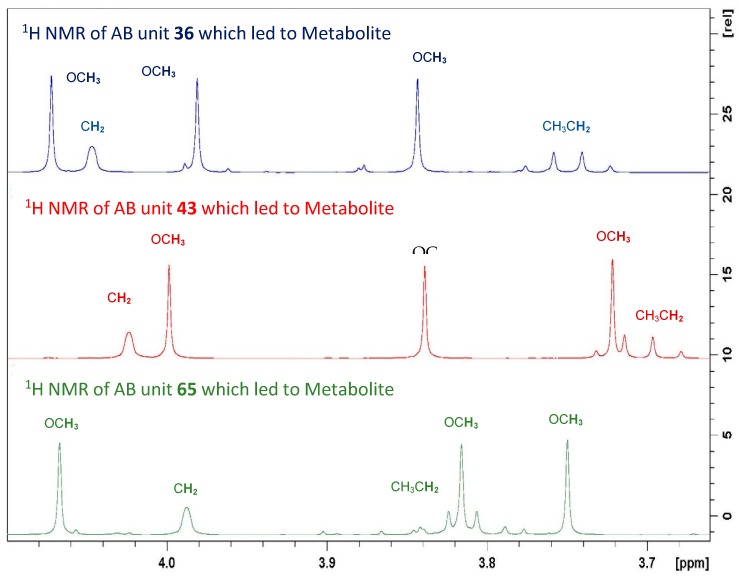
^1^H NMR spectrums of A/B units **36**, **43** and **65** which were used to synthesize metabolites **5A**, **5B** and **5C** respectively.

Comparison of ^1^H NMR run in deuterated chloroform of A/B units **36**, **43** and **65** which were used to synthesize its corresponding metabolites **5A**, **5B** and **5C**, respectively, showed a clear difference between the ^1^H NMR spectrums (Figure 3). A close up of the 3.7 to 5 ppm region showed a clear distinction in the positions of the three methoxy peaks and the position of the benzylic CH_2_ group. It was comforting to confirm that the three A/B units were indeed distinguishable with each isomer synthesized from its corresponding starting material.

#### 2.3.4. Metabolite **5D**

**Scheme 17:**
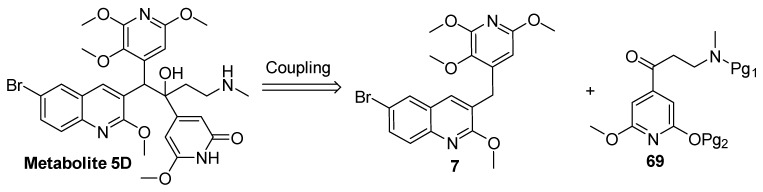
Proposed synthesis of metabolite **5D**.

Metabolite **5D** was synthesized from coupling reaction between previously synthesized A/B unit **7** and a suitably bis-protected Mannich base **69** (Scheme 17).

**Scheme 18:**
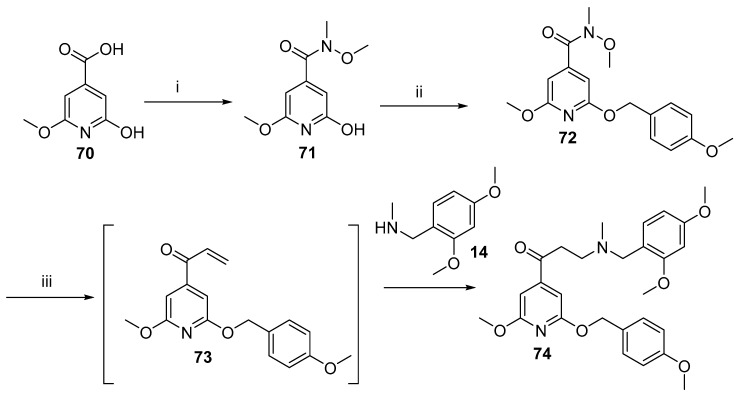
Synthesis of intermediate **74**. Reagents and conditions: (i) EDCI, HOBT, HNMeOMe.HCl. TEA, 24 h, 70%; (ii) NaH, DMF, PMB-Cl, 18 h, 32%; (iii) vinylMgBr, THF, 0 °C then **14**, H_2_O, 64%.

Synthesis of suitably protected Mannich base **69** began with pyridine **70**, which was converted to Weinreb amide **71** (Scheme 18). After experimenting with various protecting groups, *p*-methoxybenzyl (PMB) protecting group was used to protect **71** to yield **72**. Addition of vinyl magnesium bromide to **72** followed by addition of dimethoxy benzyl amine **14** and water gave desired Mannich base **74**.

**Scheme 19:**
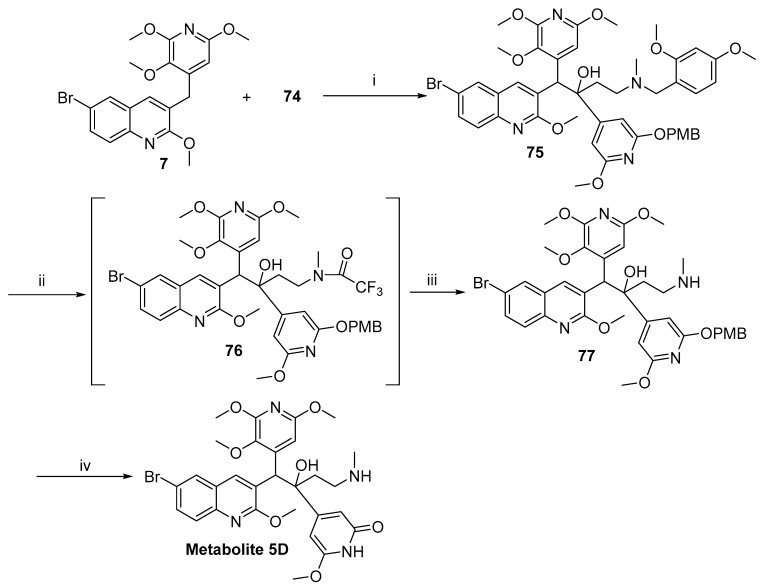
Synthesis of metabolite **5D**. Reagents and conditions: (i) LDA, THF, −75 °C, 1.5 h then **55**, −75 °C, 5 h, 67%; (ii) TFAA, DCM, 1 h; (iii) Cs_2_CO_3_, MeOH, −20 °C, 48 h, 53%; (iv) TFA:DCM (1:4), 15 min, 95%.

A/B unit **7** and Mannich base **74** were coupled using standard coupling conditions (Scheme 19). It gave desired DARQ **75** in 67% yield. Removal of DMB group using established conditions gave **77** in 53% over 2 steps. The final deprotection of the PMB group using a TFA:DCM (1:4) mixture gave desired metabolite **5D** in 95% yield. 

#### 2.3.5. Metabolite **5E**

**Scheme 20:**
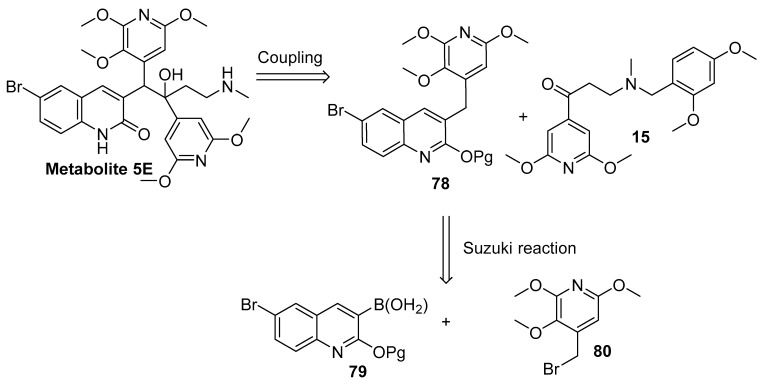
Proposed synthesis of metabolite **5E**.

Metabolite **5E** was demethylated on the methoxy group on the quinoline A-ring and mono-demethylated in the dimethylamine D-chain (Scheme 20). Metabolite **5E** was synthesized from coupling reaction between A/B unit **78** and Mannich base **15**. A/B unit **78** was accessed via Suzuki reaction between boronic acid **79** and bromide **80**.

**Scheme 21:**
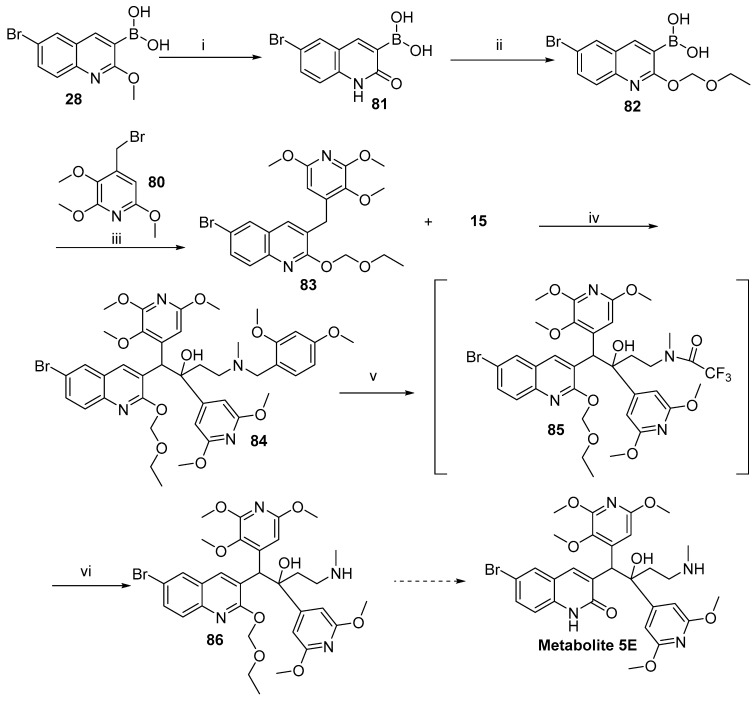
Synthesis of key intermediates **83** and **86**. Reagents and conditions: (i) 1M BBr_3_ in DCM, DMF, 24 h, 91%; (ii) DIPEA, chloromethyl ethyl ether, DMF, 66 h, 69%; (iii) Cs_2_CO_3_, Pd(PPh_3_)_4_, PhMe/DMF, 50 °C (sealed tube), 2.5 h, 71%; (iv) LDA, THF, −75 °C, 1.5 h then **15**, −75 °C, 5 h, 53%; (v) TFAA, DCM, 1 h; (vi) Cs_2_CO_3_, MeOH, −20 °C, 96 h, 63%.

The synthesis of A/B unit **78** began with boronic acid **31** which was demethylated using 1M BBr_3_ solution in DCM (Scheme 21). The reaction was carried out in DMF to give 91% yield of **81**. DCM and acetonitrile were also screened but only provided low to modest yields of **81** most likely due to solubility issues. **81** was protected with EOM group to form **82** in 69% yield. The Suzuki reaction between **82** and bromide **80** [14] proceeded well to yield A/B unit **83** in 71% yield. The coupling reaction between **83** and Mannich base **15** was successful which gave DARQ **84**. Deprotection of the DMB group was achieved using standard conditions to yield **86** in 63% over 2 steps. 

**Scheme 22:**
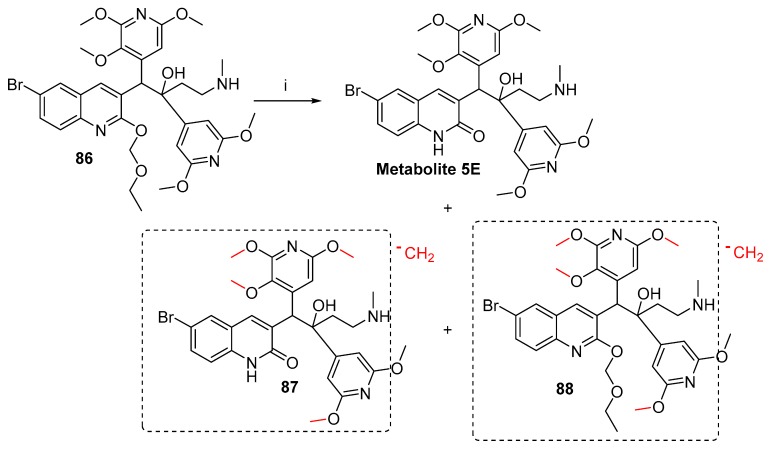
Synthesis of metabolite **5E**. Reagents and conditions: (i) 1M BBr_3_ in DCM, DCM, −75 °C, 3 h then −20 °C, 72 h, 14%.

The removal of the EOM protecting group of **86** was problematic (Scheme 22). EOM group deprotection is normally carried out in acidic conditions (e.g., using 2M HCl in diethyl ether at room temperatures). However, the EOM group off substrate **86**, was not cleaved even in strong acidic conditions at refluxing temperatures (Table 3, conditions 1, 2). Various other conditions were screened (Table 3). Conditions 3–6 led either to decomposition or gave back unreacted starting materials. When a 1M BBr_3_ solution in DCM was used (conditions 7, 8), it was possible to remove the EOM protecting group to yield metabolite **5E**. However, it also generated byproducts **87** and **88** in which a methoxy group was also removed. After the careful optimisation of the reaction temperature and the equivalence of BBr_3_, reaction mixture containing higher ratio of the desired product **5E** along with demethylated byproducts **87** and **88** could be obtained (condition 8). The mixture products **5E**, **87** and **88** were separated into pure fractions using preparative supercritical fluid chromatography to give a pure batch of metabolite **5E**.

## 3. Materials and Methods

### General

Melting points were determined on an Electrothermal 2300 melting point apparatus. NMR spectra were obtained on a Bruker Avance 400 spectrometer at 400 MHz for ^1^H and 100 MHz for ^13^C spectra and are referenced to Me_4_Si. Chemical shifts and coupling constants are recorded in units of ppm and Hz, respectively. Low resolution atmospheric pressure chemical ionization mass spectra ([M + H]) of intermediates were measured for methanol solutions on a ThermoFinnigan Surveyor MSQ mass spectrometer. Thin-layer chromatography was carried out on aluminium-backed silica gel plates (Merck 60 F_254_) with visualization of components by UV light (254 nm) and/or exposure to I_2_. Column chromatography was carried out on silica gel (Merck 230–400 mesh) unless stated otherwise. Alumina for column chromatography was Merck aluminium oxide 90 (standardised). Analysis of the final test compounds was carried out on an Agilent 1200-6110 LCMS system, using the following conditions; Column: Sunfrie C-18, 4.6 × 50 mm; Mobile phase: ACN (0.05%TFA)-water (0.05%TFA); Gradient: 5% ACN to 95% ACN in 1.0 min, hold 1.0 min, total 2.5 min; flow rate: 1.8 mL/min; LC detector: UV 214 nm, 254 nm; MS ([M + H]): atmospheric pressure electrospray ionisation; MS cone voltage: (V) Positive 4000, Negative 3000. All test compounds were determined to have >95% purity.

*(6-Bromo-2-methoxyquinolin-3-yl)boronic acid* (**28**). A solution of 2,2,6,6-tetramethylpiperidine (14.4 mL, 84.8 mmol) in THF (100 mL, dist. Na) at −78 °C was treated with *n*-BuLi (33 mL, 2.5 M in hexanes, 82.5 mmol), the solution was then warmed to −20 °C for 20 min and then cooled to −78 °C. A solution of 6-bromo-2-methoxyquinoline (10.0 g, 42.0 mmol) and triisopropylborate (20.0 mL, 87.2 mmol) in THF (100 mL, dist. Na) was added dropwise and the orange solution was stirred for 3 h at −78 °C, warmed to −40 °C and then quenched with sat. aq. NH_4_Cl (500 mL). The mixture was diluted with water (1 L) and the white precipitate was filtered, triturated with hexanes and dried to give **28** (11.17 g, 94%) as a white solid [22]. ^1^H NMR (DMSO-d_6_) δ 8.44 (s, 1H), 8.15–8.18 (m, 3H), 7.76 (dd, *J* = 8.8, 2.3 Hz, 1H), 7.68 (d, *J* = 8.9 Hz, 1H), 3.99 (s, 3H). LRMS: calculated for C_10_H_9_BBrNO_3_: 281.0; found: [M + H − OH + OCH_3_] = 296.2.

*N,2,6-Trimethoxy-N-methylisonicotinamide* (**11**). Oxalyl chloride (1.34 mL, 15.8 mmol) was added to a suspension of 2,6-dimethoxyisonicotinic acid (2.41 g, 13.2 mmol) in DCM (70 mL) and DMF (0.20 mL, 2.6 mmol) at r.t.. The mixture was stirred for 1 h to give a colourless solution which was cooled to 0 °C. *N*,*O*-dimethylhydroxylamine hydrochloride (1.42 g, 14.6 mmol) and pyridine (3.51 mL, 28.9 mmol) were added sequentially and the mixture was stirred at r.t. for 18 h, then partitioned between EtOAc and sat. aq. NaHCO_3_. Column chromatography with hexanes:EtOAc (2:1) gave **11** as a light yellow oil (2.49 g, 83%) [22]. ^1^H NMR (CDCl_3_) δ 6.47 (s, 2H), 3.93 (s, 6H), 3.58 (br s, 3H), 3.32 (s, 3H). LRMS: calculated for C_10_H_14_N_2_O_4_: 226.1; found: [M + H] = 227.2.

*3-((2,4-Dimethoxybenzyl)(methyl)amino)-1-(2,6-dimethoxypyridin-4-yl)propan-1-one* (**15**). Vinylmagnesium bromide (17.7 mL of a 1N solution in THF, 17.7 mmol) was added to a solution of **11** (2.00 g, 8.84 mmol) in dry THF (30 mL) at 0 °C. The brown solution was warmed to r.t. for 1 h then a solution of *N*-methyl-2,4-dimethoxybenzylamine (4.00 g, 22.0 mmol) in THF (10 mL), and water (10 mL) was added. The solution was stirred at r.t. for 1 h, then partitioned between EtOAc and water. The solution was dried and evaporated to give a brown oil, which was chromatographed. Elution with EtOAc/hexanes gave fore fractions, then elution with EtOAc gave **15** (2.27 g, 68%) as a light yellow oil [22]. ^1^H NMR (CDCl_3_) δ 7.13 (d, *J* = 8.9 Hz, 1H), 6.73 (s, 2H), 6.44–6.41 (m, 2H), 3.59 (s, 6H), 3.88 (s, 3H), 3.53 (s, 3H), 3.50 (s, 2H), 3.12 (t, *J* = 7.0 Hz, 1H), 2.84 (t, *J* = 7.0 Hz, 2H), 2.26 (s, 3H). LRMS: calculated for C_20_H_26_N_2_O_5_: 374.2; found: [M + H] = 375.3.

General Coupling Procedure. *n*-BuLi (0.91 mL of a 2N solution in cyclohexane, 1.81 mmol) was added at −30 °C under dry nitrogen to a solution of dry diisopropylamine (0.25 mL, 1.81 mmol) in dry THF (6 mL) and the solution was stirred at this temperature for 10 min, then cooled to −78 °C. A solution of **7** (0.63 g, 1.51 mmol) in dry THF (6 mL) was added dropwise and the mixture was stirred at −78 °C for 90 min, to give a dark, wine-red colored solution. A solution of **15** (0.56 g, 1.51 mmol) in dry THF (7 mL) was added and the reaction mixture was stirred at this temperature for 5 h. Water (100 mL) was added and the mixture was extracted with EtOAc (2x). The combined organic extract was washed with sat. aq. NaHCO_3_ solution, and brine, then dried (Na_2_SO_4_) and the solvent removed under reduced pressure. The residue was purified by flash column chromatography.

*1-(6-Bromo-2-methoxyquinolin-3-yl)-4-((2,4-dimethoxybenzyl)(methyl)amino)-2-(2,6-dimethoxypyridin-4-yl)-1-(2,3,6-trimethoxypyridin-4-yl)butan-2-ol* (**16**). The product was prepared from **7** and **15** using the General Coupling Procedure above. Column chromatography with EtOAc:hexanes (1:1) gave fore fractions, then **15** as a mixture of isomers (75%), as a yellow foam which were used crude for the next step. LRMS: calculated for C_39_H_45_BrN_4_O_9_: 792.2; found: [M + H] = 793.2.

*1-(6-Bromo-2-methoxyquinolin-3-yl)-2-(2,6-dimethoxypyridin-4-yl)-4-(methylamino)-1-(2,3,6-trimethoxypyridin-4-yl)butan-2-ol* (**3**). To a solution of **16** (1.38 g, 1.87 mmol) in DCM (10 mL) cooled to 0 °C, was added triethylamine (0.57 mL, 4.11 mmol) and trifluoroacetic anhydride (0.52 mL, 3.73 mmol). The reaction mixture was stirred for 1 h, poured onto sat. aq. NaHCO_3_ (50 mL), extracted with DCM (3 × 10 mL). The combined organic layers were dried over Na_2_SO_4_, filtered and concentrated under reduced pressure to obtain a yellowish residue. The crude material was dissolved in MeOH (100 mL) and cooled to −78 °C. Cesium carbonate (1.82 g, 5.59 mmol) was added and the reaction mixture was stirred at −20 °C for 45 h. Reaction mixture was added water (100 mL) and extracted with EtOAc (3 × 50 mL). The combined organic layers washed with brine (100 mL), dried over Na_2_SO_4_, filtered and concentrated under reduced pressure to obtain a yellow residue. Column chromatography with DCM/MeOH (9:1) gave 3* as a foamy solid (0.74 g, 63%). ^1^H NMR (CDCl_3_, 400 MHz) δ 8.73 (s, 1H), 8.12 (s, 1H), 7.83 (dd, *J* = 4.7, 2.2 Hz, 1H), 7.68 (d, *J* = 8.9 Hz, 1H), 7.60 (dd, *J* = 8.9, 2.2 Hz, 1H), 7.54–7.51 (m, 2H), 7.22 (s, 1H), 6.55 (br s, 3H), 6.49 (s, 2H), 5.49 (s, 1H), 5.38 (s, 1H), 4.21 (s, 3H), 4.01 (s, 3H), 3.93 (s, 3H), 3.87 (s, 9H), 3.82 (s, 9H), 3.81 (s, 3H), 3.80 (s, 3H), 2.63–2.51 (m, 2H), 2.32–2.23 (m, 2H), 2.21 (s, 3H), 2.13 (s, 3H), 1.88–1.75 (m, 2H), 1.64–1.53 (m, 2H). (no OH, NH observed). Found: [M + H]^+^ = 643.2. HRMS: calculated for C_30_H_35_BrN_4_O_7_: 642.1689; found: 642.1701.

*1-(2,6-Dimethoxypyridin-4-yl)-3-(dimethylamino)propan-1-one* (**24**). Vinylmagnesium bromide (32 mL of a 1N solution in THF, 32 mmol) was added to a solution of **11** (2.45 g, 10.8 mmol) in dry THF (100 mL) at 0 °C. The brown solution was warmed to r.t. for 1 h then dimethylamine (32 mL of a 2N solution in THF, 64 mmol) and water (30 mL) were added. The solution was stirred at r.t. for 1 h, then partitioned between EtOAc and water. The solution was dried and evaporated and column chromatography with DCM:MeOH (95:5) eluted impurities while DCM:MeOH (9:1) gave **24** as an oil (0.81 g, 31%) [22]. ^1^H NMR (CDCl_3_) δ 6.74 (s, 2H), 3.95 (s, 6H), 3.06 (t, *J* = 7.0 Hz, 2H), 2.72 (t, *J* = 7.0 Hz, 2H), 2.27 (s, 6H). LRMS: calculated for C_12_H_18_N_2_O_3_: 238.1; found: [M + H] = 239.1.

*1-(2,6-Dimethoxypyridin-4-yl)prop-2-en-1-one* (**12**). To a solution of **24** (3.00 g, 11.0 mmol) in DCM (50 mL) was added iodomethane (15.5 g, 109.0 mmol). The reaction mixture was stirred at r.t. for 14 h. Reaction mixture was washed with water (3 × 50 mL). The organic layer was collected and washed with brine (100 mL), dried over Na_2_SO_4_, filtered and concentrated under reduced pressure to obtain a yellow residue. Column chromatography with hexanes:EtOAc (2:1) gave **12** as an oil (0.87 g, 41%). ^1^H NMR (CDCl_3_) δ 7.26 (s, 1H), 6.96 (dd, *J* = 17.2, 10.6 Hz, 1H), 6.43 (dd, *J* = 13.3, 1.4 Hz, 1H), 6.00 (dd, *J* = 10.6, 1.4 Hz, 1H), 3.96 (s, 6H). Found: [M + H] = 194.1. HRMS: calculated for C_10_H_11_NO_3_: 193.0739; found: 193.0730.

*1-(2,6-Dimethoxypyridin-4-yl)-3-((diphenylmethylene)amino)propan-1-one* (**21**). To a solution of **12** (0.87 g, 4.45 mmol) in THF (10 mL), benzophenone imine (2.42 g, 13.4 mmol) was added. The reaction mixture was stirred at r.t. for 0.5 h. Water (50 mL) was then added to the reaction mixture and extracted with EtOAc (3 × 50 mL). The combined organic fractions were dried over Na_2_SO_4_, filtered and concentrated under reduced pressure to obtain a yellow residue. Column chromatography with hexanes:EtOAc (9:1) gave **21** as an oil (1.13 g, 68%). ^1^H NMR (CDCl_3_) δ 7.60–7.15 (m, 10H), 6.75 (s, 2H), 3.94 (s, 6H), 3.76 (t, *J* = 6.8 Hz, 2H), 3.29 (t, *J* = 6.8 Hz, 2H). Found: [M + H] = 375.2. HRMS: calculated for C_23_H_22_N_2_O_3_: 374.1630; found: 374.1638.

*1-(6-Bromo-2-methoxyquinolin-3-yl)-2-(2,6-dimethoxypyridin-4-yl)-4-((diphenylmethylene)amino)-1-(2,3,6-trimethoxypyridin-4-yl)butan-2-ol* (**26**). The product was prepared from **7** and **21** using the General Coupling Procedure above. Column chromatography with EtOAc:hexanes (9:1) gave fore fractions, then **26** as a mixture of isomers (35%), as a yellow foam. ^1^H NMR (CDCl_3_, 400 MHz) δ 8.85 (s, 1H), 8.22 (s, 1H), 7.92–7.87 (m, 2H), 7.69–7.30 (m, 21H), 7.18–7.06 (m, 2H), 6.96–6.87 (m, 4H), 6.65–6.42 (m, 3H), 5.56 (s, 1H), 5.44 (s, 1H), 4.18 (s, 3H), 4.00 (s, 3H), 3.92 (s, 3H), 3.88 (s, 3H), 3.86 (s, 6H), 3.84 (s, 3H), 3.82 (s, 3H), 3.82 (s, 3H), 3.80 (s, 6H), 3.40 (s, 3H), 3.22–3.12 (m, 2H), 3.01–2.40 (m, 2H), 2.11–1.85 (m, 2H), 1.70–1.40 (m, 2H). Found: [M + H] = 793.2. HRMS: calculated for C_42_H_41_BrN_4_O_7_: 792.2159; found: 792.2160.

*4-Amino-1-(6-bromo-2-methoxyquinolin-3-yl)-2-(2,6-dimethoxypyridin-4-yl)-1-(2,3,6-trimethoxypyridin-4-yl)butan-2-ol* (**4**). To a solution of **29** (0.42 g, 0.528 mmol) in DCM (10 mL) was added sodium carbonate (0.40 g, 3.75 mmol) and hydroxylamine hydrochloride (0.279 g, 4.01 mmol) at 0 °C. The reaction mixture was stirred at r.t. for 48 h. The reaction mixture was added water (50 mL) and extracted with EtOAc (3 × 50 mL). The combined organic fractions were dried over Na_2_SO_4_, filtered and concentrated under reduced pressure to obtain a yellow residue. Column chromatography with DCM:MeOH (9:1) gave 4* as foamy solid (0.175 g, 53%) as a mixture of isomers. ^1^H NMR (DMSO, 400 MHz) δ 8.81 (s, 1H), 8.22–8.16 (m, 3H), 7.72–7.51 (m, 4H), 7.19 (s, 1H), 6.52 (s, 1H), 6.46–6.41 (m, 4H), 5.42 (s, 1H), 5.31 (s, 1H), 4.17 (s, 3H), 3.93 (s, 3H), 3.86 (s, 3H), 3.82 (s, 3H), 3.81 (s, 3H), 3.75 (s, 3H), 3.74 (s, 3H), 3.73 (s, 3H), 3.73 (s, 9H), 3.31 (s, 3H), 2.57–2.45 (m, 2H), 2.26–2.13 (m, 2H), 1.85–1.76 (m, 2H), 1.68–1.40 (m, 2H) (no OH, NH observed). Found: [M + H] = 629.1. HRMS: calculated for C_29_H_33_BrN_4_O_7_: 628.1533; found: 628.1542.

*(2,6-Dimethoxypyridin-3-yl)boronic acid* (**30**). To a solution of 2,6-dimethoxypyridine (10 g, 71.84 mmol) and *N*,*N*-diisopropylamine (0.50 mL, 3.59 mmol) in THF (200 mL, dist. Na) at −40 °C under nitrogen was added *n*-BuLi (43.10 mL, 86.21 mmol) dropwise. The resultant solution was stirred at −40 °C for 5 min, and then warmed to 0 °C and stirred at this temperature for a further 3 h. The solution was then again cooled to −40 °C, and triisopropylborate (24.87 mL, 107.76 mmol) was added dropwise, and the mixture stirred at r.t. for another 1 h. Water (50 mL) was added and the solvent was removed in vacuo. To the residue, 1M NaOH (100 mL) was added and the aqueous layer was washed with EtOAc (2 × 100 mL). The aqueous layer was then acidified to pH 3 and a solid precipitated. This solid was filtered and dried to afford the product **30** (8.10 g, 61%) [22]. ^1^H NMR (DMSO-d_6_) δ 7.87 (d, *J* = 7.9 Hz, 1H), 6.36 (d, *J* = 7.9 Hz, 1H), 3.90 (s, 3H), 3.87 (s, 3H). LRMS: calculated for C_7_H_10_BNO_4_: 183.1; found: Found: [M + H] = 184.2.

*2,6-Dimethoxypyridin-3-ol* (**31**). To a solution of **30** (8.00 g, 43.49 mmol) in THF (150 mL, dist. Na) at 0 °C was added dropwise 32% peracetic acid in acetic acid (21.53 mL, 86.98 mmol) over 10 min. The resultant solution was stirred at r.t. for 2 h. A 10% solution of sodium sulfite (75 mL) was then added and the mixture stirred at r.t. for 0.5 h. The solvent was evaporated and the residue partitioned between EtOAc and water. The aqueous layer was extracted twice and the organic layer dried and evaporated. Column chromatography with 9:1 hexanes/EtOAc afforded the product **31** (6.05 g, 90%) as white solid [22]. ^1^H NMR (CDCl_3_) δ 7.12 (d, *J* = 8.3 Hz, 1H), 6.21 (d, *J* = 8.2 Hz, 1H), 4.90 (s, 1H), 7.00 (s, 3H), 3.86 (s, 3H). LRMS: calculated for C_7_H_9_NO_3_: 155.1; found: [M + H] = 156.7.

*3-(Ethoxymethoxy)-2,6-dimethoxypyridine* (**32**). To a solution of **31** (6.45 g, 40.97 mmol) in DMF (70 mL, anhydrous) at 0 °C was added 60% sodium hydride in mineral oil (41.97 g, 9.16 mmol) in portions. The mixture was warmed to r.t. and stirred for 1 h. 1-Chloro-2-methoxyethane (4.37 mL, 47.11 mmol) was then added, and the resultant mixture stirred at r.t. for a further 2 h. The reaction was diluted with brine (100 mL) and extracted with EtOAc three times. The organic layer was washed with brine three times, dried and evaporated. Column chromatography with 19:1 hexanes/EtOAc afforded the product **32** (8.14 g, 93%) as an oil [22]. ^1^H NMR (CDCl_3_) δ 7.41–7.33 (m, 1H), 6.26–6.17 (m, 1H), 5.15 (d, *J* = 1.9 Hz, 2H), 3.98 (d, *J* = 1.8 Hz, 3H), 3.87 (d, *J* = 2.0 Hz, 3H), 3.77 (dq, *J* = 1.8, 7.1 Hz, 2H), 1.22 (dt, *J* = 2.9, 7.0 Hz, 3H). LRMS: calculated for C_10_H_15_NO_4_: 213.1; found: [M + H] = 214.1.

*3-(Ethoxymethoxy)-2,6-dimethoxyisonicotinaldehyde* (**33**). To a solution of **32** (4.00 g, 18.78 mmol) and *N*,*N*-diisopropylamine (0.13 mL, 0.94 mmol) in THF (60 mL, dist. Na) at −40 °C under nitrogen was added *n*-BuLi (14.09 mL, 28.17 mmol) dropwise. The resultant solution was stirred at −40 °C for 5 min, and then warmed to 0 °C and stirred at this temperature for a further 3 h. The solution was then again cooled to −40 °C, and 1-formylpiperidine (3.75 mL, 33.80 mmol) was added dropwise, and the mixture stirred at r.t. for another 1 h. Acetic acid (7.5 mL) was added and the solvent was removed in vacuo. The resultant mixture was partitioned between EtOAc and water, and the organic fraction dried and evaporated. Column chromatography with 19:1 hexanes/EtOAc afforded the product **33** (2.30 g, 51%) [22]. ^1^H NMR (CDCl_3_) δ 10.39 (s, 1H), 6.61 (s, 1H), 6.19 (s, 2H), 4.02 (s, 3H), 3.88 (s, 3H), 3.78 (q, *J* = 10.1 Hz, 2H), 1.21 (t, *J* = 7.1 Hz, 3H). LRMS: calculated for C_11_H_15_NO_5_: 241.1; found: [M + H] = 242.2.

*(3-(Ethoxymethoxy)-2,6-dimethoxypyridin-4-yl)methanol* (**34**). A mixture of **33** (1.20 g, 4.98 mmol) in MeOH (30 mL, anhydrous) at 0 °C was added sodium borohydride (0.38 g, 9.96 mmol). The reaction mixture was stirred at r.t. for 2 h. The solvent was then removed and the residue partitioned between EtOAc and water. The organic layer was dried and evaporated to afford the product **34** (1.12 g, 93%). ^1^H NMR (CDCl_3_) δ 6.31 (s, 1H), 5.08 (s, 2H), 4.59 (d, *J* = 6.5 Hz, 2H), 3.96 (s, 3H), 3.88 (s, 3H), 3.81–3.75 (m, 2H), 3.03 (t, *J* = 6.6 Hz, 1H), 1.26–1.22 (m, 3H). Found: [M + H] = 244.2. HRMS: calculated for C_11_H_17_NO_5_: 243.1107; found: 243.1110.

*4-(Bromomethyl)-3-(ethoxymethoxy)-2,6-dimethoxypyridine* (**35**). To a solution of **34** (1.12 g, 4.61 mmol) and triethylamine (1.03 mL, 7.37 mmol) in DCM (20 mL, anhydrous) at r.t., mesyl chloride (0.43 mL, 5.53 mmol) was added dropwise. After 30 min, the reaction was diluted with DCM (20 mL) and the organic layer washed with sat. aq. NaHCO_3_, dried and evaporated. The residue was dissolved in acetone (40 mL, anhydrous), lithium bromide (1.20 g, 14.0 mmol) added, and the mixture heated at reflux for 30 min. The solution was then cooled and the solvent evaporated, and the residue partitioned between EtOAc and water. The aqueous layer was extracted twice with EtOAc and the organic layer was dried and evaporated to give the product **35** (1.40 g, 99%). ^1^H NMR (CDCl_3_) δ 6.32 (s, 1H), 5.14 (s, 2H), 4.45 (s, 2H), 3.95 (s, 3H), 3.87 (s, 3H), 3.83 (q, *J* = 7.1 Hz, 2H), 1.26 (t, *J* = 7.1 Hz, 3H). Found: [M + H] = 306.1. HRMS: calculated for C_11_H_16_BrNO_4_: 305.0263; found: 305.0265.

*6-Bromo-3-((3-(ethoxymethoxy)-2,6-dimethoxypyridin-4-yl)methyl)-2-methoxyquinoline* (**36**). A mixture of **28** (1.84 g, 6.56 mmol), **35** (2.00 g, 6.56 mmol) and cesium carbonate (4.49 g, 14.0 mmol) in toluene (40 mL, anhydrous) and DMF (20 mL, anhydrous) was purged with nitrogen. Pd(PPh_3_)_4_ (0.38 g, 0.33 mmol) was then added, the mixture purged with nitrogen then heated to 35 °C under nitrogen for 1 h. The reaction was partitioned between EtOAc and water and the organic fraction was dried and evaporated. Column chromatography (9:1 hexanes/EtOAc) gave the product **36** (3.01 g, 76%). ^1^H NMR (CDCl_3_) δ 7.76 (d, *J* = 2.2 Hz, 1H), 7.69 (d, *J* = 8.9 Hz, 1H), 7.61 (dd, *J* = 8.9, 2.2 Hz, 1H), 7.53 (s, 1H), 6.02 (s, 1H), 5.07 (s, 2H), 4.07 (s, 3H), 4.05 (s, 2H), 3.97 (s, 3H), 3.83 (s, 3H), 3.75 (q, *J* = 7.1 Hz, 2H), 1.18 (t, *J* = 7.1 Hz, 3H). ^13^C NMR (CDCl_3_) δ 161.1, 158.2, 155.4, 145.4, 144.4, 136.3, 132.8, 132.2, 129.4, 128.7, 126.9, 125.2, 117.3, 101.5, 97.4, 65.7, 53.9, 53.8, 53.7, 30.1, 15.3. Found: [M + H] = 463.1. HRMS: calculated for C_21_H_23_BrN_2_O_5_: 462.0790; found: 462.0794.

*1-(6-Bromo-2-methoxyquinolin-3-yl)-4-((2,4-dimethoxybenzyl)(methyl)amino)-2-(2,6-dimethoxypyridin-4-yl)-1-(3-(ethoxymethoxy)-2,6-dimethoxypyridin-4-yl)butan-2-ol* (**37**). The product was prepared from **36** and **15** using the General Coupling Procedure above. Column chromatography with EtOAc:hexanes (1:1) gave fore fractions, then **37** as a mixture of isomers (69%), as a yellow foam which were used crude for the next step. LRMS: calculated for C_41_H_49_BrN_4_O_10_: 836.3; found: [M + H] = 837.2.

*1-(6-Bromo-2-methoxyquinolin-3-yl)-2-(2,6-dimethoxypyridin-4-yl)-1-(3-(ethoxymethoxy)-2,6-dimethoxypyridin-4-yl)-4-(methylamino)butan-2-ol* (**39**). To a solution of **37** (1.87 g, 2.24 mmol) in DCM (100 mL) cooled to 0 °C, was added triethylamine (0.69 mL, 4.93 mmol) and trifluoroacetic anhydride (0.62 mL, 4.47 mmol). The reaction mixture was stirred for 1 h, poured onto sat. aq. NaHCO_3_ (150 mL), extracted with DCM (3 × 100 mL). The combined organic layers were dried over Na_2_SO_4_, filtered and concentrated under reduced pressure to obtain a yellowish residue. The crude material was dissolved in MeOH (100 mL) and cooled to −78 °C. Cesium carbonate (1.82 g, 5.60 mmol) in water (3 mL) was added and the reaction mixture was stirred at −20 °C for 72 h. Reaction mixture was added water (150 mL) and extracted with EtOAc (3 × 100 mL). The combined organic layers washed with brine (100 mL), dried over Na_2_SO_4_, filtered and concentrated under reduced pressure to obtain a yellow residue. Column chromatography with DCM:MeOH (9:1) gave **39** (0.75 g, 49%) as a mixture of isomers. ^1^H NMR (CDCl_3_, 400 MHz) δ 8.69 (s, 1H), 8.04 (s, 1H), 7.83–7.80 (m, 2H), 7.68 (d, J = 8.8 Hz, 1H), 7.60 (dd, J = 8.9, 2.2 Hz, 1H), 7.56–7.49 (m, 2H), 7.28–7.26 (m, 2H), 6.58 (s, 2H), 6.48 (s, 2H), 5.49 (s, 1H), 5.38 (s, 1H), 5.21 (q, J = 4.6 Hz, 2H), 4.82–4.73 (m, 2H), 4.18 (s, 3H), 4.01–3.98 (m, 2H), 3.98 (s, 6H), 3.87 (s, 3H), 3.85 (s, 3H), 3.83 (s, 3H), 3.82 (s, 3H), 3.80 (s, 6H), 3.79 (s, 3H), 3.79–3.73 (m, 2H), 2.65–2.53 (m, 2H), 2.32–2.22 (m, 2H), 2.22 (s, 3H), 2.12 (s, 3H), 1.92–1.82 (m, 2H), 1.63–1.55 (m, 2H), 1.39 (t, J = 7.1 Hz, 3H), 1.23 (t, J = 7.1 Hz, 3H) (no OH, NH observed). Found: [M + H] = 687.2. HRMS: calculated for C_32_H_39_BrN_4_O_8_: 686.1951; found: 686.1955.

*4-(1-(6-Bromo-2-methoxyquinolin-3-yl)-2-(2,6-dimethoxypyridin-4-yl)-2-hydroxy-4-(methylamino)butyl)-2,6-dimethoxypyridin-3-ol* (**5A**). To a solution of **39** (0.70 g, 1.02 mmol) in dioxane (5 mL) was added 1 M HCl (5 mL). The reaction mixture was stirred for 24 h, poured onto sat. aq. NaHCO_3_ (10 mL), extracted with EtOAc (3 × 10 mL). The combined organic layers were dried over Na_2_SO_4_, filtered and concentrated under reduced pressure to obtain a yellowish residue. Column chromatography with EtOAc:MeOH (9:1) gave fore fractions, followed by isomer A of 5A* (0.13 g, 20%). Elution with EtOAc:MeOH (4:1) gave isomer B of 5A* (0.18 g, 27%). Isomer A: ^1^H NMR (CDCl_3_, 400 MHz) δ 8.48 (s, 1H), 7.90 (d, *J* = 2.0 Hz, 1H), 7.68–7.60 (m, 2H), 6.51 (s, 2H), 6.08 (s, 1H), 5.00 (s, 1H), 4.15 (s, 3H), 3.88 (s, 9H), 3.61 (s, 3H), 2.65–2.60 (m, 1H), 2.40–2.32 (m, 1H), 2.19 (s, 3H), 1.90–1.85 (m, 1H), 1.77–1.69 (m, 1H), (no OH, NH observed) Found: [M + H] = 629.2. HRMS: calculated for C_29_H_33_BrN_4_O_7_: 628.1533; found: 628.1543. Isomer B: ^1^H NMR (CDCl_3_, 400 MHz) δ 8.28 (s, 1H), 7.78 (d, *J* = 1.9 Hz, 1H), 7.58–7.51 (m, 2H), 6.32 (s, 2H), 6.18 (s, 1H), 5.04 (s, 1H), 4.01 (s, 3H), 3.83 (s, 6H), 3.81 (s, 3H), 3.77 (s, 3H), 2.95–2.90 (m, 1H), 2.74–2.68 (m, 1H), 2.51 (s, 3H), 2.20–2.02 (m, 2H) (no OH, NH observed) Found: [M + H] = 629.2. HRMS: calculated for C_29_H_33_BrN_4_O_7_: 628.1533; found: 628.1543.

*4-((6-Bromo-2-methoxyquinolin-3-yl)methyl)-5,6-dimethoxypyridin-2(1H)-one* (**41**). To a solution of **7** (0.60 g, 1.44 mmol) in DMF (5 mL) was added lithium chloride (0.30 g, 7.18 mmol) and *p*-toluenesulfonic acid (1.24 g, 7.18 mmol). The reaction mixture was heated at 120 °C for 1 h. Reaction mixture was washed with water, extracted with EtOAc (3 × 20 mL). The organic fractions were collected and washed with brine (100 mL), dried over Na_2_SO_4_, filtered and concentrated under reduced pressure to obtain a yellow residue. Column chromatography with EtOAc gave **41** (0.52 g, 90%). ^1^H NMR (DMSO) δ 12.0 (s, 1H), 7.89 (d, *J* = 2.2 Hz, 1H), 7.58 (dd, *J* = 8.7, 2.2 Hz, 1H), 7.55 (s, 1H), 7.24 (d, *J* = 8.8 Hz, 1H), 6.16 (s, 1H), 3.91 (s, 3H), 3.78 (s, 3H), 3.76 (s, 2H), 3.64 (s, 3H). Found: [M + H] = 405.1. HRMS: calculated for C_18_H_17_BrN_2_O_4_: 404.0372; found: 404.0374.

*6-Bromo-3-((6-(ethoxymethoxy)-2,3-dimethoxypyridin-4-yl)methyl)-2-methoxyquinoline* (**43**). To a solution of **41** (0.52 g, 1.29 mmol) in DCM (25 mL) was added diisopropylethylamine (0.34 mL, 1.94 mmol) followed by chloromethyl ethyl ether (0.24 mL, 2.58 mmol). The mixture was warmed to 50 °C and stirred for 18 h. The reaction was washed with water (50 mL) and extracted with EtOAc (3 × 50 mL). The organic fractions were collected and washed with brine (100 mL), dried over Na_2_SO_4_, filtered and concentrated under reduced pressure to obtain a yellow residue. Column chromatography with hexanes:EtOAc (9:1) gave **43** (0.30 g, 49%). ^1^H NMR (CDCl_3_) δ 7.77 (d, *J* = 2.1 Hz, 1H), 7.67 (d, *J* = 8.9 Hz, 1H), 7.63–7.60 (m, 2H), 6.04 (s, 1H), 5.75 (s, 2H), 4.02 (s, 2H), 4.00 (s, 3H), 3.84 (s, 3H), 3.72 (s, 3H), 3.70 (q, *J* = 7.1 Hz, 2H), 1.22 (t, *J* = 7.0 Hz, 3H). ^13^C NMR (CDCl_3_) δ 159.7, 157.8, 155.7, 144.7, 144.2, 136.9, 135.8, 132.4, 129.3, 129.0, 127.0, 125.2, 117.7, 101.4, 91.2, 65.9, 60.7, 53.8, 53.7, 30.2, 15.4. Found: [M + H] = 463.1. HRMS: calculated for C_21_H_23_BrN_2_O_5_: 462.0790; found: 462.0788.

*1-(6-Bromo-2-methoxyquinolin-3-yl)-4-((2,4-dimethoxybenzyl)(methyl)amino)-2-(2,6-dimethoxypyridin-4-yl)-1-(6-(ethoxymethoxy)-2,3-dimethoxypyridin-4-yl)butan-2-ol* (**44**). The product was prepared from **43** and **15** using the General Coupling Procedure above. Column chromatography with EtOAc:hexanes (1:1) gave fore fractions, then **44** as a mixture of isomers (83%), as a yellow foam which were used crude for the next step. LRMS: calculated for C_41_H_49_BrN_4_O_10_: 836.3; found: [M + H] = 837.2.

*4-(1-(6-Bromo-2-methoxyquinolin-3-yl)-2-(2,6-dimethoxypyridin-4-yl)-2-hydroxy-4-(methylamino)butyl)-5,6-dimethoxypyridin-2(1H)-one* (**5B**). To a solution of **44** (0.44 g, 0.53 mmol) in DCM (50 mL) cooled to 0 °C, was added triethylamine (0.16 mL, 1.16 mmol) and trifluoroacetic anhydride (0.15 mL, 1.06 mmol). The reaction mixture was stirred for 1 h, poured onto sat. aq. NaHCO_3_ (150 mL), extracted with DCM (3 × 50 mL). The combined organic layers were dried over Na_2_SO_4_, filtered and concentrated under reduced pressure to obtain a yellowish residue. The crude material was dissolved in MeOH (100 mL) and cooled to −78 °C. Cesium carbonate (0.52 g, 1.58 mmol) in water (3 mL) was added and the reaction mixture was stirred at −20 °C for 48 h. Reaction mixture was added water (150 mL) and extracted with EtOAc (3 × 50 mL). The combined organic layers washed with brine (100 mL), dried over Na_2_SO_4_, filtered and concentrated under reduced pressure to obtain a yellow residue. Column chromatography with EtOAc:MeOH (9:1) gave fore fractions, followed by isomer A of 5B* (0.064 g, 19%). Elution with EtOAc:MeOH (4:1) gave isomer B of 5B* (0.069 g, 21%). Isomer A: ^1^H NMR (DMSO, 400 MHz) δ 8.34 (s, 1H), 7.95 (d, J = 2.2 Hz, 1H), 7.57 (d, J = 8.7 Hz, 1H), 7.12 (d, J = 8.8 Hz, 1H), 6.56 (s, 1H), 6.42 (s, 2H), 5.24 (s, 1H), 3.91 (s, 3H), 3.90 (s, 3H), 3.76 (s, 9H), 2.53–2.48 (m, 1H), 2.36–2.31 (m, 1H), 2.24 (s, 3H), 2.03–1.70 (m, 2H) (no OH, NH observed) Found: [M + H] = 629.2. HRMS: calculated for C_29_H_33_BrN_4_O_7_: 628.1533; found: 628.1543. Isomer B: ^1^H NMR (DMSO, 400 MHz) δ 8.01 (d, J = 2.2 Hz, 1H), 7.92–7.85 (m, 1H), 7.59 (dd, J = 8.8, 1.9 Hz, 1H), 7.24 (d, J = 8.7 Hz, 1H), 7.10–7.08 (m, 1H), 6.43–6.35 (m, 2H), 5.47 (s, 1H), 3.79 (s, 6H), 3.75 (s, 3H), 3.73 (s, 3H), 3.72 (s, 3H), 2.38–2.22 (m, 1H), 2.14–2.03 (m, 1H), 2.03 (s, 3H), 1.82–1.66 (m, 2H) (no OH, NH observed) Found: [M + H] = 629.2. HRMS: calculated for C_29_H_33_BrN_4_O_7_: 628.1533; found: 628.1543.

*5-(Ethoxymethoxy)-2-methoxypyridine* (**49**). To a solution of 6-pyridin-3-ol (3.20 g, 25.4 mmol) in DMF (50 mL, anhydrous) at 0 °C was added 60% sodium hydride in mineral oil (1.23 g, 30.5 mmol) in portions. The mixture was warmed to r.t. and stirred for 1 h. 1-Chloro-2-methoxyethane (2.73 mL, 25.9 mmol) was then added, and the resultant mixture stirred at r.t. for a further 4 h. The reaction was diluted with brine (100 mL) and extracted with EtOAc three times. The organic layer was washed with brine three times, dried and evaporated. Column chromatography with 19:1 hexanes/EtOAc afforded the product **49** (4.44 g, 95%). ^1^H NMR (CDCl_3_) δ 7.95 (d, *J* = 3.0 Hz, 1H), 7.34–7.31 (m, 1H), 6.66 (d, *J* = 9.0 Hz, 1H), 5.13 (s, 2H), 3.88 (s, 3H), 3.72 (q, *J* = 7.1 Hz, 2H), 1.22 (t, *J* = 7.0 Hz, 3H). Found: [M + H] = 184.6. HRMS: calculated for C_9_H_13_NO_3_: 183.0895; found: 183.0899.

*5-(Ethoxymethoxy)-2-methoxyisonicotinaldehyde* (**50**). To a solution of **49** (3.64 g, 19.9 mmol) and *N*,*N*-diisopropylamine (0.14 mL, 0.1 mmol) in THF (60 mL, dist. Na) at −40 °C under nitrogen was added *n*-BuLi (15.0 mL, 30.0 mmol) dropwise. The resultant solution was stirred at −40 °C for 5 min, and then warmed to 0 °C and stirred at this temperature for a further 3 h. The solution was then again cooled to −40 °C, and 1-formylpiperidine (3.98 mL, 36.0 mmol) was added dropwise, and the mixture stirred at r.t. for another 1 h. Acetic acid (8 mL) was added and the solvent was removed in vacuo. The resultant mixture was partitioned between EtOAc and water, and the organic fraction dried and evaporated. Column chromatography with 9:1 hexanes/EtOAc afforded the product **50** (2.74 g, 65%). ^1^H NMR (CDCl_3_) δ 10.42 (s, 1H), 8.29 (s, 1H), 7.07 (s, 1H), 5.30 (s, 2H), 3.92 (s, 3H), 3.78 (q, *J* = 7.1 Hz, 2H), 1.23 (t, *J* = 7.1 Hz, 3H). Found: [M + MeOH] = 244.2. HRMS: calculated for C_10_H_13_NO_4_: 211.0845; found: 211.0849.

*5-Hydroxy-2-methoxyisonicotinaldehyde* (**51**). A solution of **50** (2.74 g, 13.0 mmol) and 3M hydrochloric acid (30 mL) in THF (40 mL, dist. Na) was heated at 40 °C for 3 h. The solution was then cooled, diluted with water, and the pH adjusted to 7 using NaHCO_3_. The aqueous layer was then extracted with EtOAc three times, and the organic layer dried and evaporated. Column chromatography with 4:1 hexanes/EtOAc afforded the product **51** (1.28 g, 64%) [23]. ^1^H NMR (CDCl_3_) δ 9.97 (s, *J* = 0.4 Hz, 1H), 9.46 (s, 1H), 8.08 (s, 1H), 6.93 (d, *J* = 0.5 Hz, 1H), 3.94 (s, 3H). LRMS: calculated for C_7_H_7_NO_3_: 153.0; found: [M + H] = 154.2.

*2,5-Dimethoxyisonicotinaldehyde* (**52**). A mixture of **51** (1.28 g, 8.36 mmol) and potassium carbonate (1.73 g, 12.5 mmol) in DMF (50 mL, anhydrous) was heated at 50 °C for 10 min. Methyl iodide (1.42 g, 10.0 mmol) was then added and the mixture stirred at this temperature for 2 h. The resultant solution was diluted with EtOAc and washed with brine three times. The organic layer was dried and evaporated to afford the product **52** (1.39 g, 99%) [23]. ^1^H NMR (CDCl_3_) δ 10.43 (s, 1H), 8.01 (s, 1H), 7.08 (d, *J* = 0.6 Hz, 1H), 3.97 (s, 3H), 3.91 (s, 3H). LRMS: calculated for C_8_H_9_NO_3_: 167.1; found: [M + H] = 168.2.

*4-(1,3-Dioxolan-2-yl)-2,5-dimethoxypyridine* (**53**). A mixture of **52** (0.20 g, 1.20 mmol), pyridinium p-toluenesulfonate and ethylene glycol (0.37 g, 5.99 mmol) in toluene (6 mL, anhydrous) was heated at 120 °C for 6 h. The resultant solution was diluted with EtOAc and washed with sat. NaHCO3 solution. The organic layer was dried and evaporated. Column chromatography with 9:1 hexanes/EtOAc afforded the product **53** (0.22 g, 88%). ^1^H NMR (CDCl_3_) δ 7.87 (s, 1H), 6.90 (s, 1H), 6.07 (s, 1H), 4.15–3.89 (m, 4H), 3.89 (s, 3H), 3.88 (s, 3H). Found: [M + H] = 212.2. HRMS: calculated for C_10_H_13_NO_4_: 211.0845; found: 211.0836.

*4-(1,3-Dioxolan-2-yl)-2,5-dimethoxypyridine 1-oxide* (**54**). A mixture of **53** (0.22 g, 1.04 mmol) in DCM (8 mL, anhydrous) was added 77% m-CPBA (0.47 g, 2.08 mmol) at 0 °C. The reaction mixture was stirred at r.t. for 17 h. The solvent was removed from the reaction mixture and crude product was purified using silica column chromatography with 9:1 DCM/MeOH afforded the product **54** (0.17 g, 70%). ^1^H NMR (CDCl_3_) δ 8.02 (s, 1H), 7.09 (s, 1H), 6.00 (s, 1H), 4.15–4.02 (m, 4H), 4.05 (s, 3H), 3.85 (s, 3H). Found: [M + H] = 228.2. HRMS: calculated for C_10_H_13_NO_5_: 227.0794; found: 227.0781.

*4-(1,3-Dioxolan-2-yl)-3,6-dimethoxypyridin-2-yl acetate* (**55**). A mixture of **54** (1.84 g, 8.10 mmol) in acetic anhydride (5 mL) was heated at 150 °C for 3 h. Acetic anhydride was removed from the reaction mixture in vacuo and crude product was purified using silica column chromatography with 1:1 hexanes/EtOAc afforded the product **55** (1.54 g, 71%). ^1^H NMR (CDCl_3_) δ 6.81 (s, 1H), 6.05 (s, 1H), 4.12–4.02 (m, 4H), 3.88 (s, 3H), 3.81 (s, 3H), 2.36 (s, 3H). Found: [M + H] = 270.2. HRMS: calculated for C_12_H_15_NO_6_: 269.0899; found: 269.0887.

*3,6-Dimethoxy-2-oxo-1,2-dihydropyridine-4-carbaldehyde* (**56**). A mixture of **55** (1.43 g, 5.32 mmol) in chloroform (4 mL) was added to TFA (1 mL) and stirred at r.t. for 2 h. Solvent was removed from the reaction mixture in vacuo to give the product **56** (0.83 g, 85%). ^1^H NMR (CDCl_3_) δ 10.38 (s, 1H), 9.08 (s, 1H), 5.74 (s, 1H), 4.07 (s, 3H), 3.86 (s, 3H). Found: [M + H] = 184.2. HRMS: calculated for C_8_H_9_NO_4_: 183.0532; found: 183.0521.

*2-(Ethoxymethoxy)-3,6-dimethoxyisonicotinaldehyde* (**57**). To a solution of **56** (0.59 g, 3.22 mmol) in DMF (10 mL) was added to diisopropylethylamine (1.12 mL, 6.44 mmol) followed by chloromethyl ethyl ether (0.914 g, 9.67 mmol). The mixture was stirred at r.t. for 3 h. The reaction was washed with water (50 mL) and extracted with EtOAc (3 × 50 mL). The organic fractions were collected and washed with brine (100 mL), dried over Na_2_SO_4_, filtered and concentrated under reduced pressure to obtain a yellow residue. Column chromatography with hexanes:EtOAc (9:1) gave **57** (0.54 g, 69%). ^1^H NMR (CDCl_3_) δ 10.40 (s, 1H), 6.64 (s, 1H), 5.68 (s, 2H), 3.96 (s, 3H), 3.88 (s, 3H), 3.82 (q, *J* = 7.1 Hz, 2H), 1.27 (t, *J* = 7.1 Hz, 3H). Found: [M + H] = 242.2. HRMS: calculated for C_11_H_15_NO_5_: 241.0950; found: 241.0940.

*(2-(Ethoxymethoxy)-3,6-dimethoxypyridin-4-yl)methanol* (**63**). To a mixture of **57** (0.54 g, 2.22 mmol) in MeOH (10 mL, anhydrous) at 0 °C, sodium borohydride was added (0.168 g, 4.45 mmol). The reaction mixture was stirred at r.t. for 0.5 h. The solvent was then removed and the residue partitioned between EtOAc and water. The organic layer was dried and evaporated to afford the product **63** (0.50 g, 92%). ^f1^H NMR (CDCl_3_) δ 6.36 (s, 1H), 5.65 (s, 2H), 4.68 (s, 2H), 3.85 (s, 3H), 3.83 (s, 3H), 3.80 (q, *J* = 7.1 Hz, 2H), 1.26 (t, *J* = 7.1 Hz, 3H). Found: [M + H] = 244.2. HRMS: calculated for C_11_H_17_NO_5_: 243.1107; found: 243.1110.

*4-(Bromomethyl)-2-(ethoxymethoxy)-3,6-dimethoxypyridine* (**64**). To a solution of **63** (0.40 g, 1.65 mmol) and triethylamine (0.345 mL, 2.47 mmol) in DCM (20 mL, anhydrous) at r.t., mesyl chloride (0.153 mL, 1.98 mmol) was added dropwise. After 30 min, the reaction was diluted with DCM (20 mL) and the organic layer washed with sat. aq. NaHCO_3_, dried and evaporated. The residue was dissolved in acetone (20 mL, anhydrous) and cooled to 0 °C and added lithium bromide (0.16 g, 1.82 mmol). The reaction mixture was stirred at 0 °C for 3 h. The reaction mixture was partitioned between EtOAc and water. The aqueous layer was extracted twice with EtOAc and the organic layer was dried and evaporated to give the product **64** (0.49 g, 98%). ^1^H NMR (CDCl_3_) δ 6.33 (s, 1H), 5.64 (s, 2H), 4.40 (s, 2H), 3.90 (s, 3H), 3.84 (s, 3H), 3.81 (q, *J* = 7.1 Hz, 2H), 1.26 (t, *J* = 7.1 Hz, 3H). Found: [M + H] = 306.1 HRMS: calculated for C_11_H_16_BrNO_4_: 305.0263; found: 305.0268.

*6-bromo-3-((2-(ethoxymethoxy)-3,6-dimethoxypyridin-4-yl)methyl)-2-methoxyquinoline* (**65**). A mixture of **28** (0.56 g, 1.97 mmol), **64** (0.50 g, 1.65 mmol) and cesium carbonate (1.07 g, 3.30 mmol) in toluene (20 mL, anhydrous) and DMF (10 mL, anhydrous) was purged with nitrogen. Pd(PPh_3_)_4_ (0.095 g, 0.083 mmol) was then added, the mixture purged with nitrogen then heated to 50 °C under nitrogen for 0.5 h. The reaction was partitioned between EtOAc and water and the organic fraction was dried and evaporated. Column chromatography (9:1 hexanes/EtOAc) gave the product **65** (0.77 g, 81%). ^1^H NMR (CDCl_3_) δ 7.77 (d, *J* = 2.2 Hz, 1H), 7.68 (d, *J* = 8.8 Hz, 1H), 7.61 (dd, *J* = 8.9, 2.2 Hz, 1H), 7.56 (s, 1H), 6.09 (s, 1H), 5.66 (s, 2H), 4.07 (s, 3H), 3.99 (s, 2H), 3.82 (s, 3H), 3.81 (q, *J* = 7.1 Hz, 2H), 3.75 (s, 3H), 1.25 (t, *J* = 7.1 Hz, 3H). ^13^C NMR (CDCl_3_) δ 161.2, 157.7, 153.9, 145.5, 144.5, 136.4, 135.9, 132.2, 129.3, 128.8, 126.9, 125.2, 117.3, 103.0, 90.8, 65.7, 60.8, 54.0, 53.8, 30.2, 15.4. Found: [M + H] = 463.1. HRMS: calculated for C_21_H_23_BrN_2_O_5_: 462.0790; found: 462.0794.

*1-(6-Bromo-2-methoxyquinolin-3-yl)-4-((2,4-dimethoxybenzyl)(methyl)amino)-2-(2,6-dimethoxypyridin-4-yl)-1-(2-(ethoxymethoxy)-3,6-dimethoxypyridin-4-yl)butan-2-ol* (**66**). The product was prepared from **65** and **15** using the General Coupling Procedure above. Column chromatography with EtOAc:hexanes (1:1) gave fore fractions, then **66** as a mixture of isomers (59%), as a yellow foam which were used crude for the next step.. Found: [M + H] = 837.2. HRMS: calculated for C_41_H_49_BrN_4_O_10_: 836.2632; found: 836.2662.

*1-(6-Bromo-2-methoxyquinolin-3-yl)-2-(2,6-dimethoxypyridin-4-yl)-1-(2-(ethoxymethoxy)-3,6-dimethoxypyridin-4-yl)-4-(methylamino)butan-2-ol* (**68**). To a solution of **66** (0.82 g, 0.98 mmol) in DCM (10 mL) cooled to 0 °C, triethylamine (0.30 mL, 2.16 mmol) and trifluoroacetic anhydride (0.25 mL, 1.96 mmol) were added. The reaction mixture was stirred for 20 min, poured onto sat. aq. NaHCO_3_ (150 mL), extracted with DCM (3 × 20 mL). The combined organic layers were dried over Na_2_SO_4_, filtered and concentrated under reduced pressure to obtain a yellowish residue. The crude material was dissolved in MeOH (30 mL) and cooled to −78 °C. Cesium carbonate (0.96 g, 2.94 mmol) was added and the reaction mixture was stirred at −20 °C for 48 h. Water (50 mL) was added to the reaction mixture and extracted with EtOAc (3 × 20 mL). The combined organic layers washed with brine (50 mL), dried over Na_2_SO_4_, filtered and concentrated under reduced pressure to obtain a yellow residue. Column chromatography with EtOAc:MeOH (9:1) gave **68** (0.45 g, 67%) as a mixture of isomers. ^1^H NMR (DMSO, 400 MHz) δ 8.70 (s, 1H), 8.11 (s, 1H), 7.83–7.50 (m, 6H), 7.25–7.22 (m, 1H), 6.64–6.40 (m, 5H), 5.76 (d, J = 6.0 Hz, 1H), 5.58 (d, J = 6.1 Hz, 1H), 5.51–5.43 (m, 3H), 5.37 (s, 1H), 4.20 (s, 3H), 3.97 (s, 3H), 3.88 (s, 6H), 3.82–3.78 (15H), 3.81–3.75 (m, 2H), 3.69–3.62 (m, 2H), 3.42 (s, 3H), 2.62–2.53 (m, 2H), 2.32–2.22 (m, 2H), 2.23 (s, 3H), 2.16 (s, 3H), 1.91–1.77 (m, 2H), 1.74–1.58 (m, 2H), 1.29–1.23 (m, 6H) (no OH, NH observed). Found: [M + H] = 687.2. HRMS: calculated for C_32_H_39_BrN_4_O_8_: 686.1951; found: 686.1960.

*4-(1-(6-Bromo-2-methoxyquinolin-3-yl)-2-(2,6-dimethoxypyridin-4-yl)-2-hydroxy-4-(methylamino)butyl)-3,6-dimethoxypyridin-2(1H)-one* (**5C**). Solution of **68** (0.45 g, 0.66 mmol) in THF (10 mL) cooled to 0 °C, was added 2M HCl in diethyl ether (1 mL). The reaction mixture was stirred at 0 °C for 30 min. Water (15 mL) was added to the reaction mixture and extracted with EtOAc (3 × 10 mL). The combined organic layers washed with brine (10 mL), dried over Na_2_SO_4_, filtered and concentrated under reduced pressure to obtain a yellow residue. Column chromatography with DCM:MeOH (9:1) gave 5C* (0.23 g, 54%) as mixture. ^1^H NMR (DMSO, 400 MHz) δ 8.73 (s, 1H), 8.34–8.17 (m, 3H), 7.77–7.63 (m, 3H), 7.53 (d, J = 8.8 Hz, 1H), 6.87 (s, 1H), 6.45–6.32 (m, 4H), 6.26 (s, 1H), 5.43 (s, 1H), 5.34 (s, 1H), 4.18 (s, 3H), 3.82 (s, 12H), 3.73–3.68 (m, 12H), 3.31 (s, 3H), 2.80–2.55 (m, 2H), 2.41–2.05 (m, 9H), 2.00–1.68 (3H) (no OH, NH observed). Found: [M + H] = 629.2. HRMS: calculated for C_29_H_33_BrN_4_O_7_: 628.1533; found: 628.1542.

*2-Hydroxy-6-methoxyisonicotinic acid* (**70**). Methyl 2-hydroxy-6-methoxyisonicotinate (3.74 g, 18.0 mmol) in MeOH:THF (1:1, 100 mL) was added LiOH (1.27 g, 53.0 mmol) dissolved in water (50 mL). The reaction mixture was stirred at r.t. for 3 h. The reaction mixture was concentrated under reduced pressure and added water (50 mL) and extracted with EtOAc (1 × 20 mL). The aqueous phase was added 2M HCl until pH was ~1 which led to formation of white precipitates. The solid was filtered and collected to give **70** (3.35 g, 94%). ^1^H NMR (DMSO, 400 MHz) δ 6.59 (s, 2H), 3.82 (s, 3H). Found: [M + H] = 170.2. HRMS: calculated for C_7_H_7_NO_4_: 169.0375; found: 169.0377.

*2-Hydroxy-N,6-dimethoxy-N-methylisonicotinamide* (**71**). **70** (3.35 g, 19.8 mmol) and triethylamine (9.66 mL, 69.3 mmol) in DMF (30 mL) was stirred at room temperature until homogeneous. *N*,*O*-dimethylhydroxylamine hydrochloride (4.18 g, 22.0 mmol) and hydroxybenzotriazole (2.01 g, 14.9 mmol) were added and the solution was stirred for 2 min. EDCI.HCl (4.18 g, 22.0 mmol) was added and the mixture was stirred at room temperature for 18 h. The solvent was completely removed under reduced pressure and the residue was triturated 3 times with acetone. The combined acetone triturates were concentrated to dryness and the residue was used crude for the next step (2.94 g, 70%).

*N,2-Dimethoxy-6-((4-methoxybenzyl)oxy)-N-methylisonicotinamide* (**72**). A solution of **71** (2.94 g, 14.0 mmol) in DMF (20 mL) was cooled in an ice bath. Sodium hydride 60% w/w in oil (0.83 g, 21.0 mmol) was added in portions with stirring over 5 min. 1-(Chloromethyl)-4-methoxybenzene (2.28 mL, 17.0 mmol) was then added and the solution was stirred at room temperature for 18 h. The product was partitioned between EtOAc and brine, and the combined organic layers washed with brine (50 mL), dried over Na_2_SO_4_, filtered and concentrated under reduced pressure to obtain a yellow residue. Column chromatography with hexanes:EtOAc (4:1) gave **72** (1.49 g, 32%). ^1^H NMR (CDCl_3_, 400 MHz) δ 7.40–7.36 (m, 2H), 6.92–6.88 (m, 2H), 6.51 (s, 1H), 6.47 (s, 1H), 5.31 (s, 2H), 3.93 (s, 3H), 3.81 (s, 3H), 3.81 (s, 3H), 3.57 (s, 3H), 3.31 (s, 3H). Found: [M + H] = 333.2. HRMS: calculated for C_17_H_20_N_2_O_5_: 332.1372; found: 332.1380.

*3-((2,4-Dimethoxybenzyl)(methyl)amino)-1-(2-methoxy-6-((4-methoxybenzyl)oxy)pyridin-4-yl)propan-1-one* (**74**). Vinylmagnesium bromide (8.97 mL of a 1N solution in THF, 8.75 mmol) was added to a solution of **72** (1.49 g, 4.48 mmol) in dry THF (30 mL) at 0 °C. The brown solution was warmed to r.t. for 1 h then a solution of *N*-methyl-2,4-dimethoxybenzylamine **14** (2.03 g, 11.2 mmol) in THF (10 mL), and water (20 mL) were added. The solution was stirred at r.t. for 20 min, then partitioned between EtOAc and water. The mixture was extracted with EtOAc (4 × 20 mL). The solution was dried and evaporated to give a brown oil, which was chromatographed. Elution with EtOAc/hexanes gave fore fractions, then elution with EtOAc gave **74** (1.38 g, 64%) as a light yellow oil. ^1^H NMR (CDCl_3_) δ 7.39–7.37 (m, 2H), 7.13–7.10 (m, 1H), 6.92–6.88 (m, 2H), 6.77 (d, *J* = 1.1 Hz, 1H), 6.72 (d, *J* = 1.1 Hz, 1H), 6.42–6.40 (m, 2H), 5.32 (s, 2H), 5.29 (s, 2H), 3.95 (s, 3H), 3.81 (s, 3H), 3.78 (s, 3H), 3.76 (s, 3H), 3.10 (t, *J* = 7.0 Hz, 2H), 2.83 (t, *J* = 7.0 Hz, 2H), 2.24 (s, 3H). Found: [M + H] = 481.3. HRMS: calculated for C_27_H_32_N_2_O_6_: 480.2260; found: 480.2272.

*1-(6-Bromo-2-methoxyquinolin-3-yl)-4-((2,4-dimethoxybenzyl)(methyl)amino)-2-(2-methoxy-6-((4-methoxybenzyl)oxy)pyridin-4-yl)-1-(2,3,6-trimethoxypyridin-4-yl)butan-2-ol* (**75**). The product was prepared from **7** and **74** using the General Coupling Procedure above. Column chromatography with EtOAc:hexanes (1:1) gave fore fractions, then **75** as a mixture of isomers (67%), as a yellow foam which were used crude for the next step. LRMS: calculated for C_46_H_51_BrN_4_O_10_: 898.3; found: [M + H] = 899.2.

*1-(6-Bromo-2-methoxyquinolin-3-yl)-2-(2-methoxy-6-((4-methoxybenzyl)oxy)pyridin-4-yl)-4-(methylamino)-1-(2,3,6-trimethoxypyridin-4-yl)butan-2-ol* (**77**). To a solution of **75** (1.43 g, 1.59 mmol) in DCM (30 mL) cooled to 0 °C, triethylamine (0.49 mL, 3.50 mmol) and trifluoroacetic anhydride (0.44 mL, 3.18 mmol) were added. The reaction mixture was stirred for 30 min, poured onto sat. aq. NaHCO_3_ (150 mL), extracted with DCM (3 × 20 mL). The combined organic layers were dried over Na_2_SO_4_, filtered and concentrated under reduced pressure to obtain a yellowish residue. The crude material was dissolved in MeOH (100 mL) and cooled to −78 °C. Cesium carbonate (1.55 g, 4.77 mmol) was added and the reaction mixture was stirred at −20 °C for 27 h. Water (50 mL) was added to the reaction mixture and extracted with EtOAc (3 × 20 mL). The combined organic layers were washed with brine (50 mL), dried over Na_2_SO_4_, filtered and concentrated under reduced pressure to obtain a yellow residue. Column chromatography with EtOAc:MeOH (9:1) gave **77** (0.45 g, 53%) as mixture of isomers. ^1^H NMR (CDCl_3_, 400 MHz) δ 8.73 (s, 1H), 8.10 (s, 1H), 7.83 (dd, J = 2.1 Hz, 2H), 7.68–7.50 (m, 4H), 7.39–7.23 (m, 5H), 6.93–6.84 (m, 4H), 6.62–6.45 (m, 5H), 5.47 (s, 1H), 5.35 (s, 1H), 5.25–5.17 (m, 4H), 4.19 (s, 3H), 4.01 (s, 3H), 3.93 (s, 3H), 3.88 (s, 3H), 3.86 (s, 3H), 3.82–3.79 (s, 18H), 3.39 (s, 3H), 2.61–2.49 (m, 2H), 2.31–2.21 (m, 2H), 2.19 (s, 3H), 2.12 (s, 3H), 1.86–1.68 (m, 4H) (no OH, NH observed). Found: [M + H] = 749.2. HRMS: calculated for C_37_H_41_BrN_4_O_8_: 748.2108; found: 748.2114.

*4-(1-(6-Bromo-2-methoxyquinolin-3-yl)-2-hydroxy-4-(methylamino)-1-(2,3,6-trimethoxypyridin-4-yl)butan-2-yl)-6-methoxypyridin-2-ol* (**5D**). To a solution of **77** (0.64 g, 0.85 mmol) in DCM (20 mL) cooled to 0 °C, TFA (5 mL) was added. The reaction mixture was stirred at r.t. for 15 min. The reaction mixture was concentrated under reduced pressure and was washed with sat. NaHCO_3_ (30 mL) and extracted with EtOAc (3 × 20 mL). Column chromatography with DCM:MeOH (9:1) gave 5D* (0.51 g, 95%) as mixture of isomers. ^1^H NMR (CDCl_3_, 400 MHz) δ 8.69 (s, 1H), 8.15 (s, 1H), 7.81 (dd, J = 10.9, 1.9 Hz, 2H), 7.66–7.50 (m, 4H), 7.12 (s, 1H), 6.49–6.30 (m, 3H), 5.97 (s, 2H), 5.46 (s, 1H), 5.32 (s, 1H), 4.16 (s, 3H), 3.98 (s, 3H), 3.92–3.87 (m, 6H), 3.84–3.78 (m, 12H), 3.74 (s, 3H), 3.40 (s, 3H), 2.69–2.50 (m, 4H), 2.29 (s, 3H), 2.22 (s, 3H), 2.06–1.70 (m, 4H) (no OH, NH observed). Found: [M + H] = 629.2. HRMS: calculated for C_29_H_33_BrN_4_O_7_: 628.1533; found: 628.1541.

*(6-Bromo-2-oxo-1,2-dihydroquinolin-3-yl)boronic acid* (**81**). (6-Bromo-2-methoxyquinolin-3-yl)boronic acid **28** (1.0 g, 3.56 mmol) was dissolved in DMF (5 mL) and cooled to 0 °C and 1M BBr_3_ solution was added in DCM (18.0 mL, 10.7 mmol) dropwise. The reaction mixture was stirred at r.t. for 24 h. The reaction mixture was washed with sat. NaHCO_3_ (30 mL) and extracted with EtOAc (3 × 20 mL). White precipitates formed in the organic layer which was filtered and collected to give **81** (0.862 g, 91%). ^1^H NMR (DMSO, 400 MHz) δ 12.27 (s, 1H), 8.83 (d, *J* = 9.3 Hz, 2H), 8.40 (s, 1H), 8.06 (d, *J* = 2.2 Hz, 1H), 7.72 (dd, *J* = 8.8, 2.3 Hz, 1H), 7.31 (d, *J* = 8.8 Hz, 1H). Found: [M + H − OH + MeOH] = 283.0. HRMS: calculated for C_9_H_7_BBrNO_3_: 266.9702; found: 266.9714.

*(6-Bromo-2-(ethoxymethoxy)quinolin-3-yl)boronic acid* (**82**). To a solution of **81** (1.55 g, 5.85 mmol) in DMF (30 mL, anhydrous) at 0 °C, DIPEA was added (3.06 mL, 17.5 mmol). 1-Chloro-2-methoxyethane (1.38 g, 14.6 mmol) was then added, and the resultant mixture stirred at r.t. for 48 h. The reaction was diluted with brine (50 mL) and extracted with EtOAc (3 × 20 mL). The combined organic layers washed with brine (50 mL), dried over Na_2_SO_4_, filtered and concentrated under reduced pressure to obtain a yellow solid. Crude product was triturated with hexanes:Et_2_O (1:1) mixture and filtered and collected to give **82** as a white solid (1.31 g, 69%) which was pure enough to be used for the next step. ^1^H NMR (DMSO, 400 MHz) δ 8.69 (s, 2H), 8.42 (s, 1H), 8.12 (d, *J* = 2.4 Hz, 1H), 7.83 (dd, *J* = 9.1, 2.4 Hz, 1H), 7.56 (d, *J* = 9.1 Hz, 1H), 5.73 (s, 2H), 3.55 (q, *J* = 7.1 Hz, 2H), 1.07 (t, *J* = 7.0 Hz, 3H). Found: [M + H − OH + MeOH − OH + MeOH] = 356.1. HRMS: calculated for C_12_H_13_BBrNO_4_: 325.0121; found: 325.0126.

*6-Bromo-2-(ethoxymethoxy)-3-((2,3,6-trimethoxypyridin-4-yl)methyl)quinolone* (**83**). A mixture of **82** (1.31 g, 4.06 mmol), 4-(bromomethyl)-2,3,6-trimethoxypyridine **80** (1.06 g, 4.06 mmol) and cesium carbonate (1.98 g, 6.10 mmol) in toluene (40 mL, anhydrous) and DMF (20 mL, anhydrous) was purged with nitrogen. Pd(PPh_3_)_4_ (0.47 g, 0.41 mmol) was then added, the mixture purged with nitrogen then heated to 55 °C under nitrogen for 2.5 h. The reaction was partitioned between EtOAc and water and the organic fraction was dried and evaporated. Column chromatography (9:1 hexanes/EtOAc) gave the product **83** (1.34 g, 71%). ^1^H NMR (CDCl_3_) δ 7.57–7.54 (m, 2H), 7.46–7.44 (m, 1H), 7.24 (s, 1H), 6.14 (s, 1H), 5.77 (s, 2H), 4.00 (s, 3H), 3.91 (s, 2H), 3.86 (s, 3H), 3.75 (s, 3H), 3.65 (q, *J* = 7.0 Hz, 2H), 1.19 (t, *J* = 7.1 Hz, 3H). ^13^C NMR (CDCl_3_) δ 162.5, 158.0, 155.8, 144.3, 137.4, 136.0, 135.9, 132.8, 132.8 130.5, 122.3, 117.1, 115.6, 101.4, 72.6, 64.9, 60.8, 53.9, 53.7, 30.8, 15.3. Found: [M + H] = 463.1. HRMS: calculated for C_21_H_23_BrN_2_O_5_: 462.0790; found: 462.0798.

*1-(6-Bromo-2-(ethoxymethoxy)quinolin-3-yl)-4-((2,4-dimethoxybenzyl)(methyl)amino)-2-(2,6-dimethoxypyridin-4-yl)-1-(2,3,6-trimethoxypyridin-4-yl)butan-2-ol* (**84**). The product was prepared from **83** and **15** using the General Coupling Procedure below. Column chromatography with EtOAc:hexanes (1:1) gave fore fractions, then **84** as a mixture of isomers (53%), as a yellow foam which were used crude for the next step. LRMS: calculated for C_41_H_49_BrN_4_O_10_: 836.3; found: [M + H] = 837.3.

*1-(6-Bromo-2-(ethoxymethoxy)quinolin-3-yl)-2-(2,6-dimethoxypyridin-4-yl)-4-(methylamino)-1-(2,3,6-trimethoxypyridin-4-yl)butan-2-ol* (**86**). To a solution of **84** (0.78 g, 0.93 mmol) in DCM (30 mL) cooled to 0 °C, was added triethylamine (0.28 mL, 2.04 mmol) and trifluoroacetic anhydride (0.26 mL, 1.85 mmol). The reaction mixture was stirred for 30 min, poured onto sat. aq. NaHCO_3_ (150 mL), extracted with DCM (3 × 20 mL). The combined organic layers were dried over Na_2_SO_4_, filtered and concentrated under reduced pressure to obtain a yellowish residue. The crude material was dissolved in MeOH (100 mL) and cooled to −78 °C. Cesium carbonate (0.91 g, 2.78 mmol) was added and the reaction mixture was stirred at −20 °C for 27 h. Reaction mixture was added water (50 mL) and extracted with EtOAc (3 × 20 mL). The combined organic layers washed with brine (50 mL), dried over Na_2_SO_4_, filtered and concentrated under reduced pressure to obtain a yellow residue. Column chromatography with EtOAc:MeOH (9:1) gave **86** (0.402 g, 63%) as a mixture of isomers. ^1^H NMR (DMSO, 400 MHz) δ 8.38 (s, 1H), 7.82 (s, 1H), 7.68–7.40 (m, 6H), 7.32–7.24 (m, 1H), 7.13 (s, 1H), 6.65–6.41 (m, 4H), 5.87–5.77 (m, 2H), 5.73–5.66 (m, 2H), 5.54–5.43 (m, 2H), 4.03 (s, 3H), 4.00 (s, 3H), 3.76–3.70 (m, 21H), 3.71–3.64 (m, 2H), 3.58 (s, 3H), 3.43–3.32 (m, 2H), 2.64–2.51 (m, 2H), 2.41–2.26 (m, 2H), 2.23 (s, 3H), 2.21 (s, 3H), 2.08–1.92 (m, 2H), 1.82–1.70 (m, 2H), 1.23–1.17 (m, 3H), 1.09–1.06 (m, 3H). (no OH, NH observed). Found: [M + H] = 687.2. HRMS: calculated for C_32_H_39_BrN_4_O_8_: 686.1951; found: 686.1964.

*6-Bromo-3-(2-(2,6-dimethoxypyridin-4-yl)-2-hydroxy-4-(methylamino)-1-(2,3,6-trimethoxypyridin-4-yl)butyl)quinolin-2(1H)-one* (**5E**). Solution of **86** (0.174 g, 0.25 mmol) in DCM (60 mL) cooled to −78 °C, was added 1M BBr_3_ solution in DCM (0.76 mL, 0.76 mmol). The reaction mixture was stirred at −78 °C for 4 h, followed by −20 °C for 72 h. MeOH (20 mL) was added to the reaction mixture, and concentrated under reduced pressure. Crude product was initially purified with column chromatography with DCM:MeOH (9:1) which was further purified using preparative supercritical fluid chromatography to give 5E* (0.022 g, 14%). ^1^H NMR (CDCl_3_, 400 MHz) δ 8.53 (s, 1H), 7.93 (s, 1H), 7.71–7.46 (m, 4H), 7.22–6.98 (m, 2H), 6.94–6.89 (m, 1H), 6.72–6.21 (m, 5H), 5.39 (s, 1H), 5.37 (s, 1H), 4.10 (s, 3H), 4.02 (s, 3H), 3.90 (s, 6H), 3.86 (s, 6H), 3.84 (s, 3H), 3.80 (s, 3H), 3.79 (s, 3H), 3.56 (s, 3H), 2.89–2.46 (m, 4H), 2.38 (s, 3H), 2.24 (s, 3H), 2.15–1.67 (m, 4H) (no OH, NH observed). Found: [M + H] = 629.2. HRMS: calculated for C_29_H_33_BrN_4_O_7_: 628.1533; found: 628.1504.

*The final DARQ compounds **3**, **4**, **5A**, **5B**, **5C**, **5D**, **5E** were resolved into four optical isomers using preparative supercritical fluid HPLC at BioDuro LLC (Beijing). The ^1^H NMR and ^13^C NMR spectra of key compounds are available in the Supplementary Materials.

## 4. Conclusions

Bedaquiline (**1**)—targeting the ATP synthase enzyme in the electron transport chain of Mycobacterium tuberculosis—is a key drug for the treatment of drug-resistant tuberculosis. Second generation analogue TBAJ-876 [24] **2** show much promise, with better potency and less hERG liability than bedaquiline. In an effort to aid the preclinical development of **2**, we set out to synthesize its major known desmethyl metabolites. We synthesized and fully characterised seven of these (metabolites **3**, **4** and **5A**–**5E**). This work has unequivocally identified and assigned the possible structures of major metabolites of TBAJ-876, and has provided quantities of these as authentic standards for further mass spectrometry and metabolite profiling studies. This will aid in the preclinical development of **2** as a potential second generation analogue of bedaquiline.

## Figures and Tables

**Table 1:** Physical and biological properties of bedaquiline **1** and TBAJ-876 **2**.

Property	Bedaquiline (1)	TBAJ-876 (2)
Lipophilicity (cLogP) ^a^	7.25	5.15
Solubility pH 7.4 (μM)	<0.06	<0.02
Mouse PO t _½_ (hr)	44	26
MIC (MABA) (μg/mL) ^b^	0.04	0.004
MIC (LORA) (μg/mL) ^b^	0.08	0.006
hERG IC_50_ (μM) ^c^	1.6	>30

^a^ clogP calculated by ChemDraw v19.0.0.22. (CambridgeSoft); ^b^ MIC_90_ (µg/mL); minimum inhibitory concentration for 90% inhibition of growth of M.tb strain H37Rv, determined under aerobic (replicating; MABA) or non-replicating (LORA) conditions; ^c^ Inhibition of hERG (IC_50_ in µM).

**Table 2:** Summary of conditions explored for protection of **56** with chloromethyl ethyl ether.

	Solvent	Base, Additives	Time	Product Ratio (57:58)
1	DCM	DIPEA	24 h	1:4
2	Benzene	DIPEA	24 h	0.5:9.5
3	DMF	DIPEA	24 h	4:1
4	Benzene	Ag_2_CO_3_	24 h	**58** only
5	Benzene	Ag_2_CO_3_, DIPEA	24 h	**58** only

**Table 3:** Summary of conditions explored for removal of EM protecting group from **86**.

	Reagents	Solvent	Temp	Time	Products (% Yield)
1	2M HCl diethyl ether	DCM	150 °C	24 h	-
2	4M HCl diethyl ether	benzene	150 °C	24 h	-
3	TFA	DCM	110 °C	24 h	-
4	Pyridinium *p*-toluenesulfonate	butanol	110 °C	24 h	Trace of **5E**
5	Tetra-*n*-butylammonium bromide, TMS-Cl	DCM	110 °C	24 h	-
6	CBr_4_	*i*-PrOH	110 °C	24 h	-
7	1M BBr_3_ (5 eq.)	DCM	0 °C	24 h	**5E**:**87**:**88** (~10%:8%:9%)
8	1M BBr_3_ (3 eq.)	DCM	−78 °C then −20 °C	3 h then 72 h	**5E**:**87**:**88** (~14%:5%:10%)

## Supplementary Materials

The ^1^H NMR and ^13^C NMR spectra of key compounds are available online.
