# Supplementary material for: Synthetic Studies to Help Elucidate the Metabolism of the Preclinical Candidate TBAJ-876—A Less Toxic and More Potent Analogue of Bedaquiline
Source: Molecules. 2020 Mar 20;25(6):1423. doi: 10.3390/molecules25061423 (PMC7144385; doi:10.3390/molecules25061423)

Synthetic studies to help elucidate the metabolism of the preclinical candidate TBAJ876 - a less toxic and more potent analogue of bedaquiline

Peter J. Choi[1,2*], Hamish S. Sutherland[1], Adrian Blaser[1], Amy S.T. Tong[1], Christopher B. Cooper[3], Anna M. Upton[3], Brian D. Palmer[1,2,], William A. Denny[1,2]

p.choi@auckland.ac.nz

Electronic Supplementary Information

CONTENTS

^{1}H NMR spectrum of **28, 11** 2

^{1}H NMR spectrum of **15, 3** 3

^{1}H NMR spectrum of **24, 12** 4

^{1}H NMR spectrum of **21, 4** 5

^{1}H NMR spectrum of **34, 35** 6

^{1}H NMR spectrum of **36, 39** 7

^{1}H NMR spectrum of **5A** 8

^{1}H NMR spectrum of **41, 43** 9

^{1}H NMR spectrum of **5B** 10

^{1}H NMR spectrum of **50, 51** 11

^{1}H NMR spectrum of **52, 53** 12

^{1}H NMR spectrum of **54, 55** 13

^{1}H NMR spectrum of **56, 57** 14

^{1}H NMR spectrum of **63, 64** 15

^{1}H NMR spectrum of **65, 5C** 16

^{1}H NMR spectrum of **70, 72** 17

^{1}H NMR spectrum of **77, 5D** 18

^{1}H NMR spectrum of **84, 85** 19

^{1}H NMR spectrum of **83** 20

^{1}H NMR spectrum of **5E** 21

*Figure 1: ^{1}H NMR of **28***

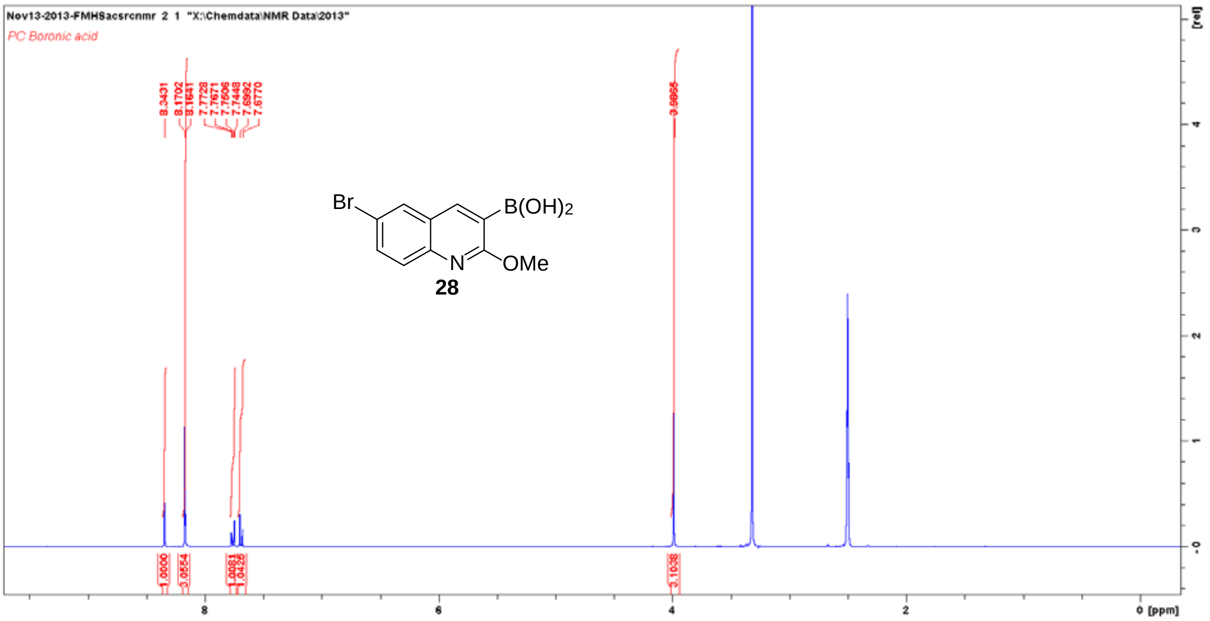

*Figure 2: ^{1}H NMR of **11***

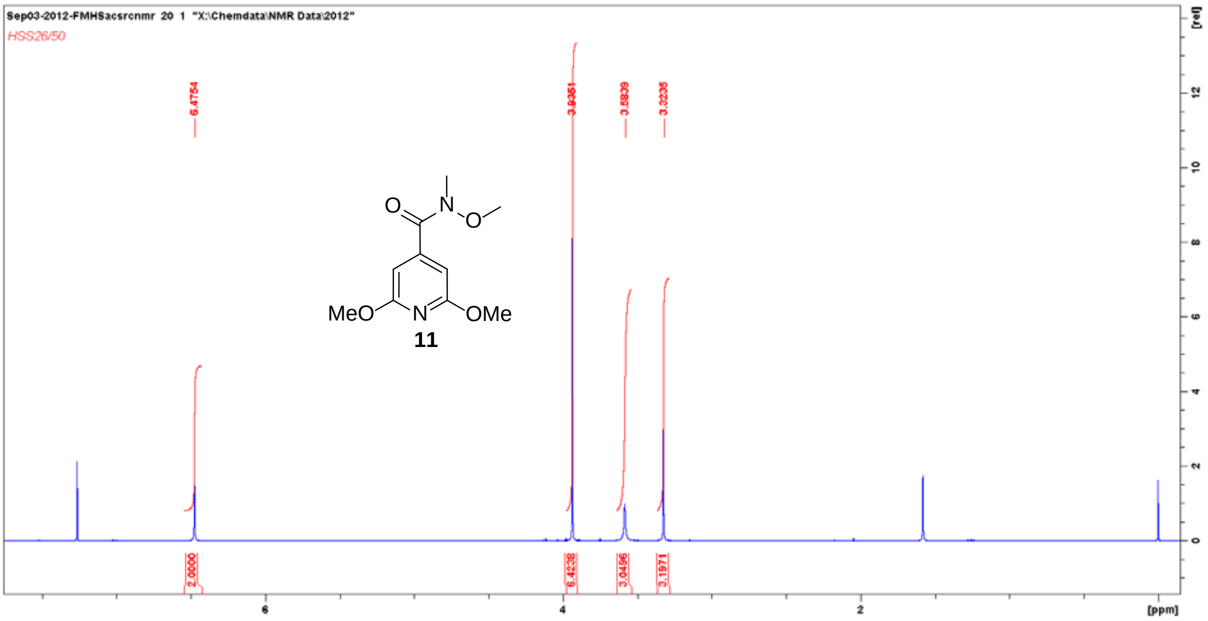

*Figure 3: ^{1}H NMR of **15***

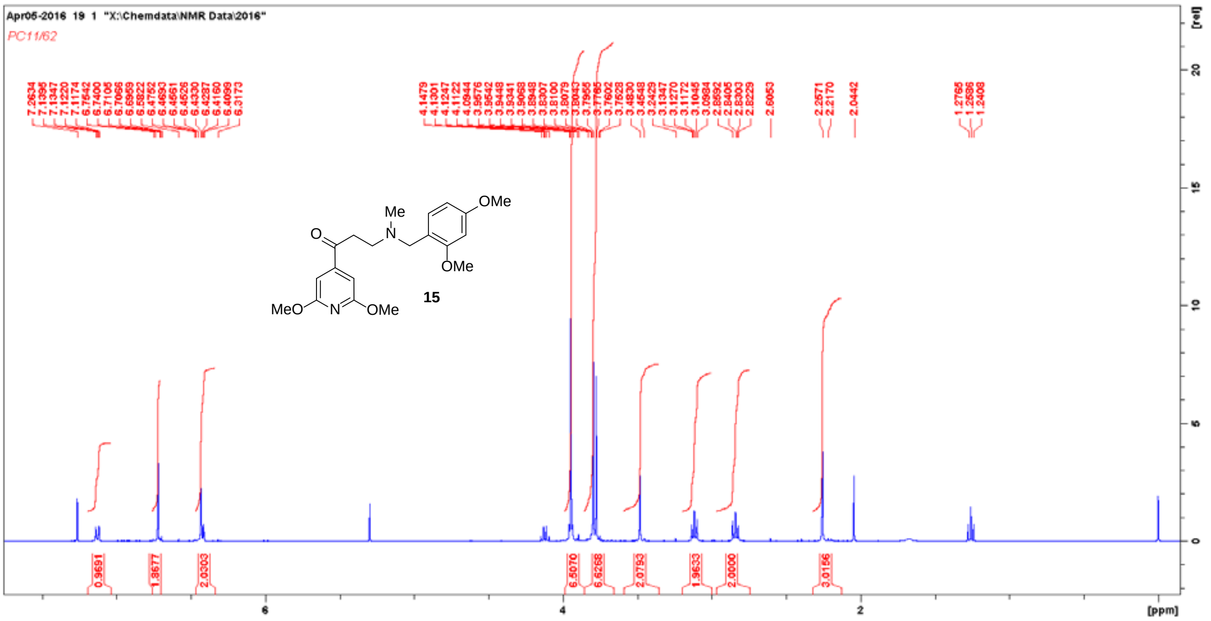

*Figure 4: ^{1}H NMR of **3***

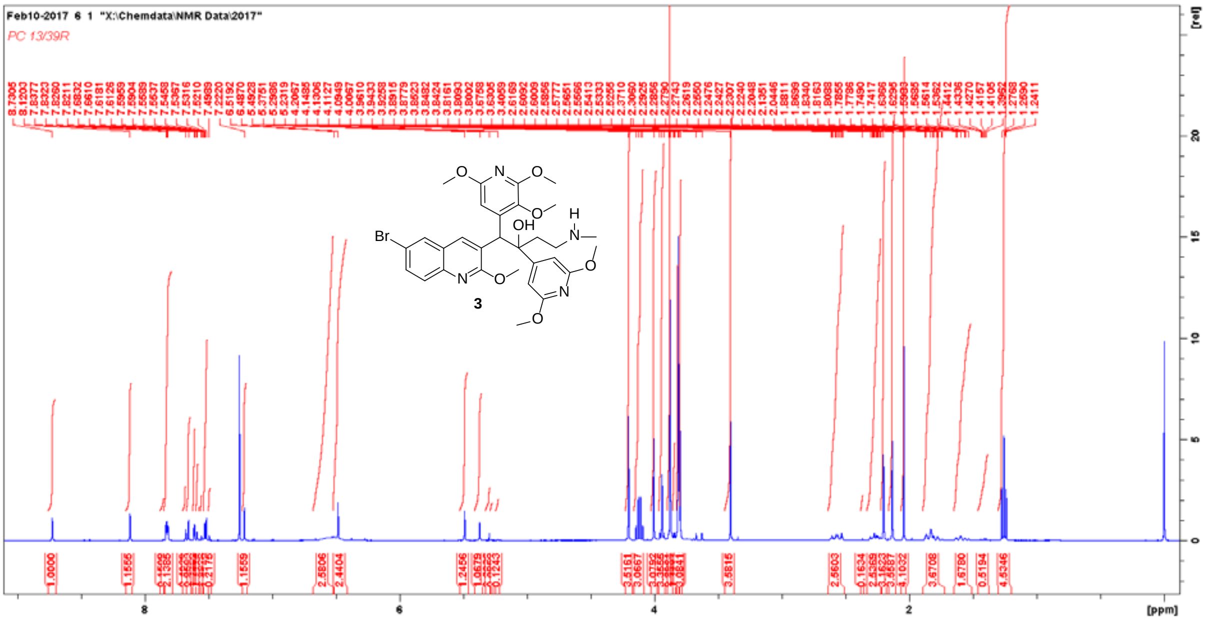

*Figure 5: ^{1}H NMR of **24***

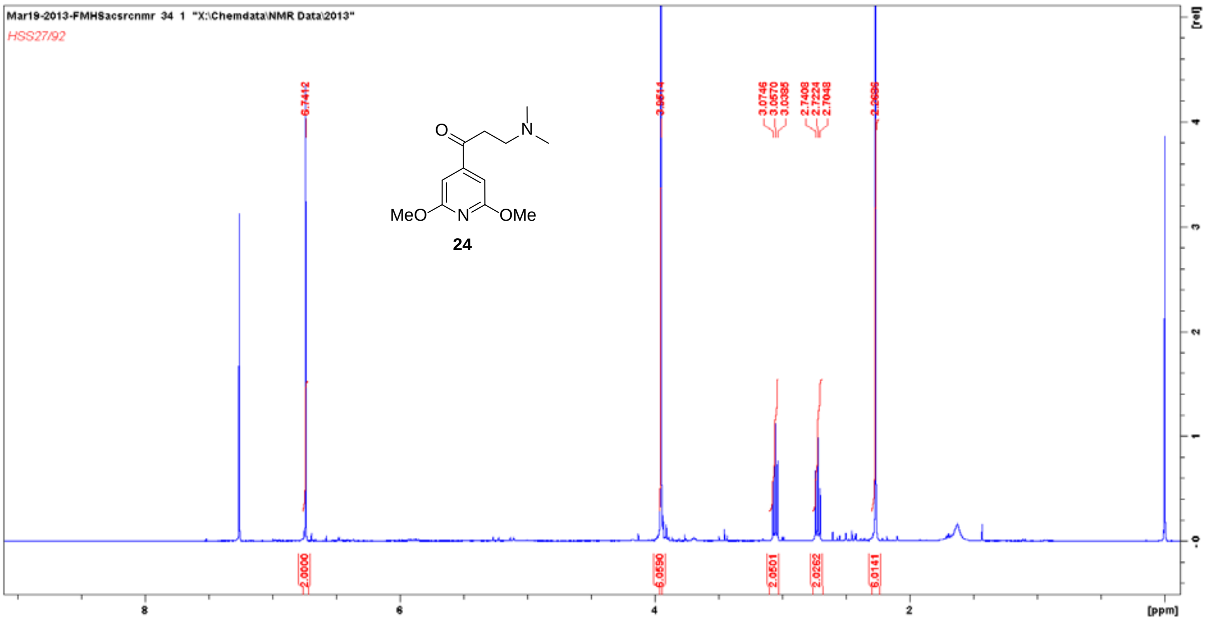

*Figure 6: ^{1}H NMR of **12***

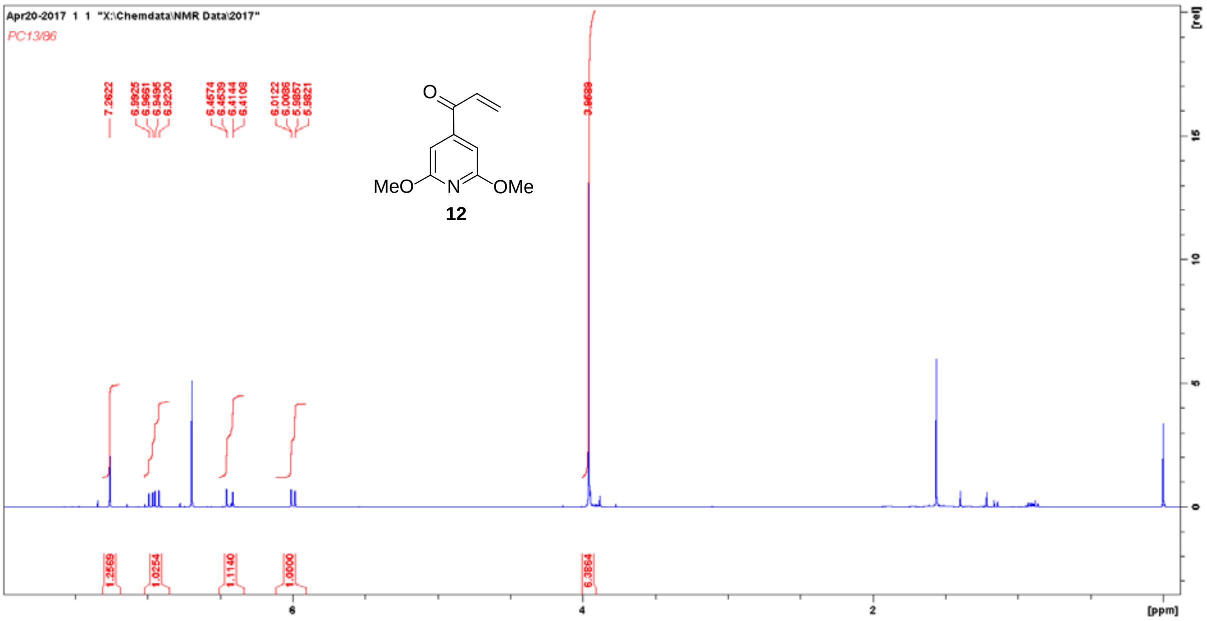

*Figure 7: ^{1}H NMR of **21***

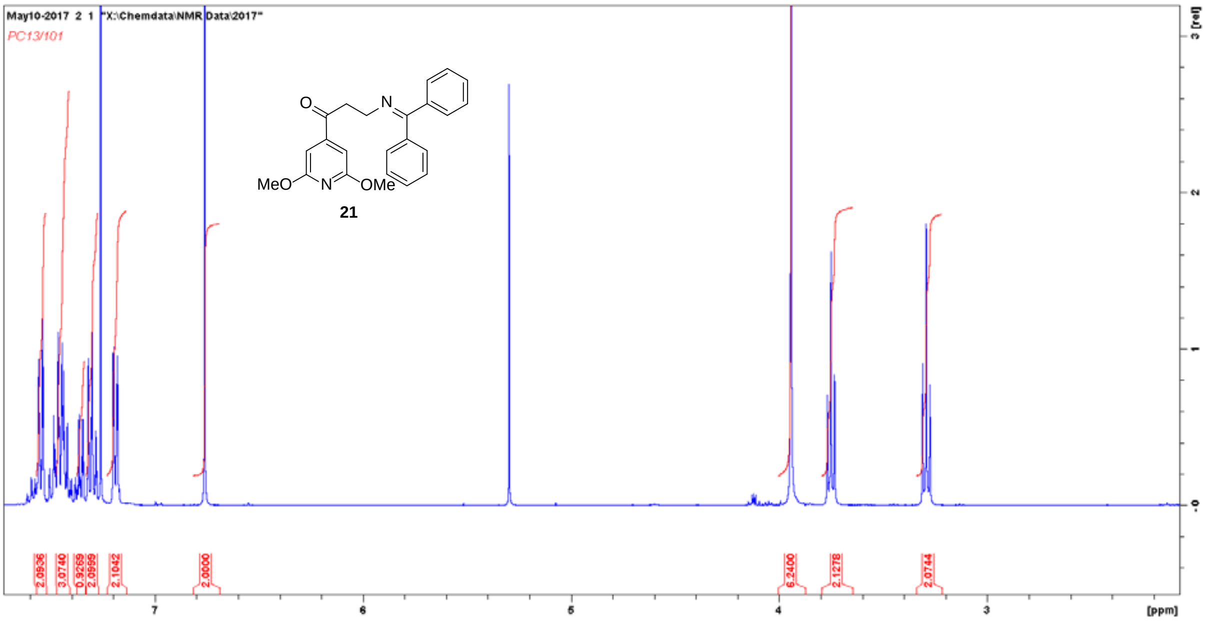

*Figure 8: ^{1}H NMR of **4***

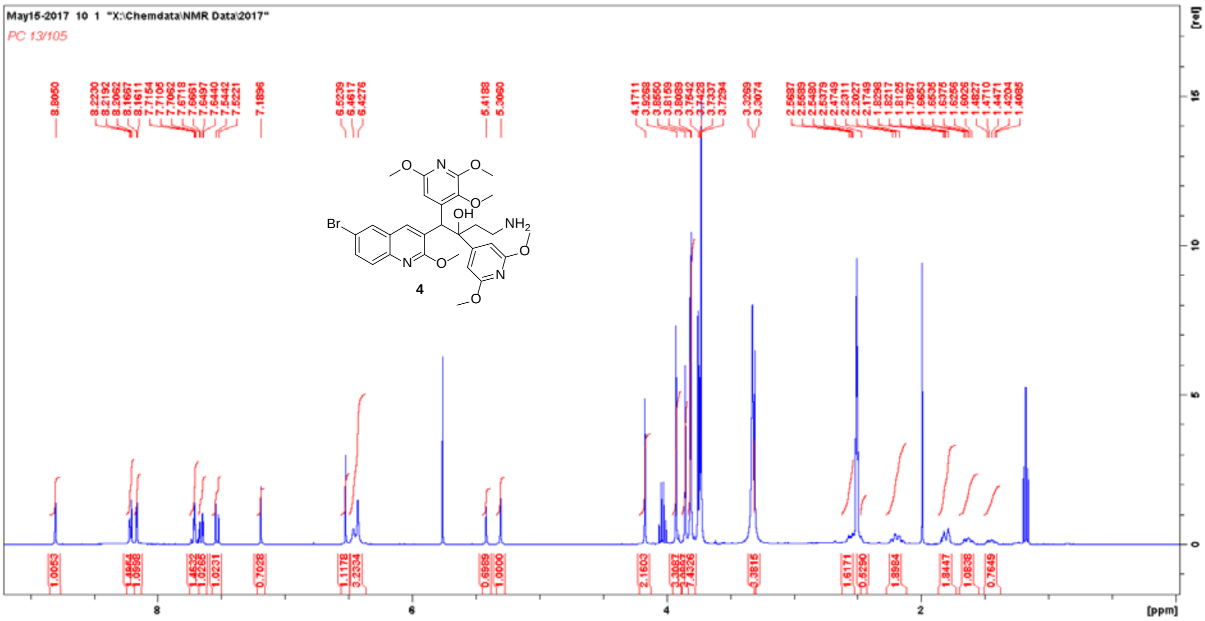

*Figure 9: ^{1}H NMR of **34***

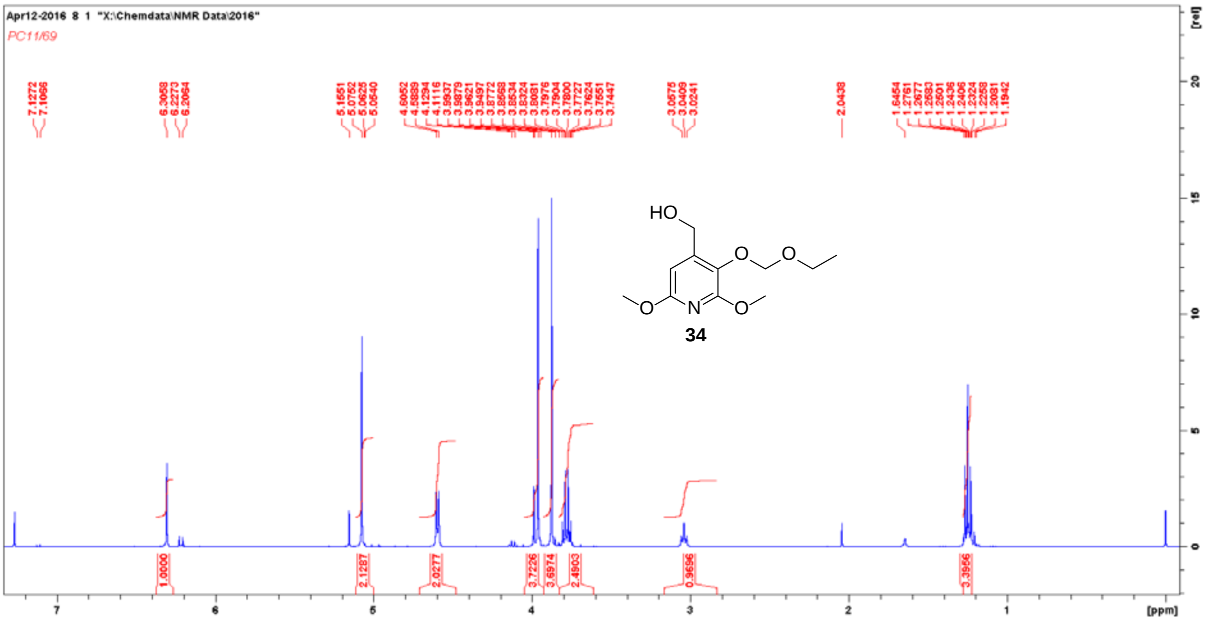

*Figure 10: ^{1}H NMR of **35***

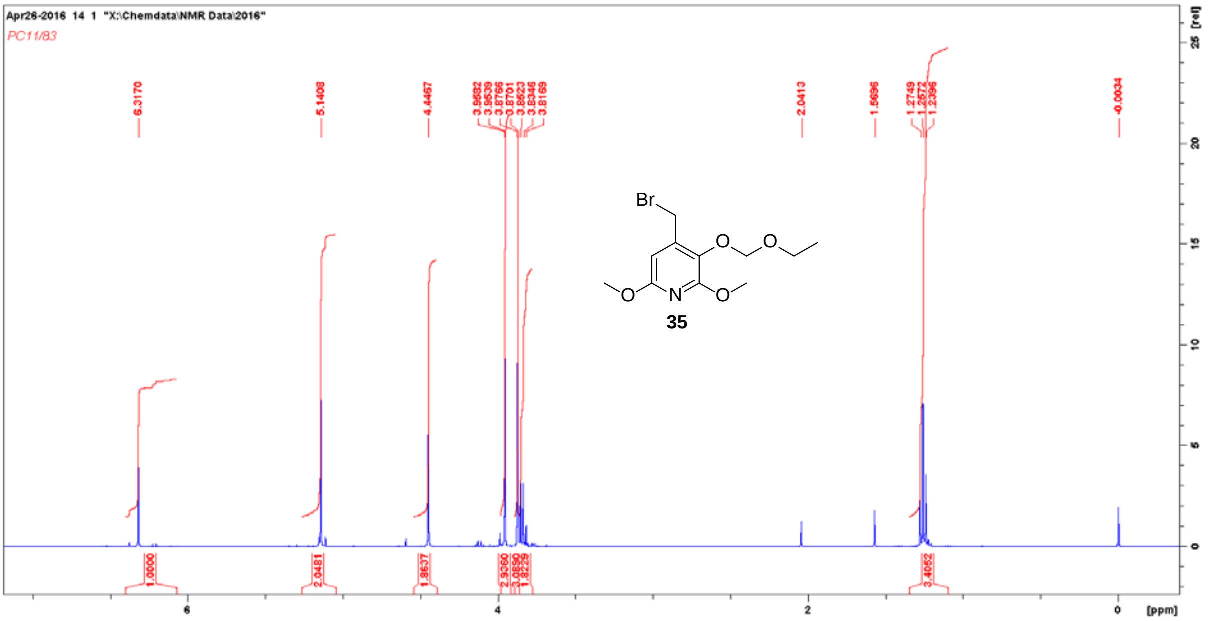

Figure 11: ^{1}H NMR of ***36***

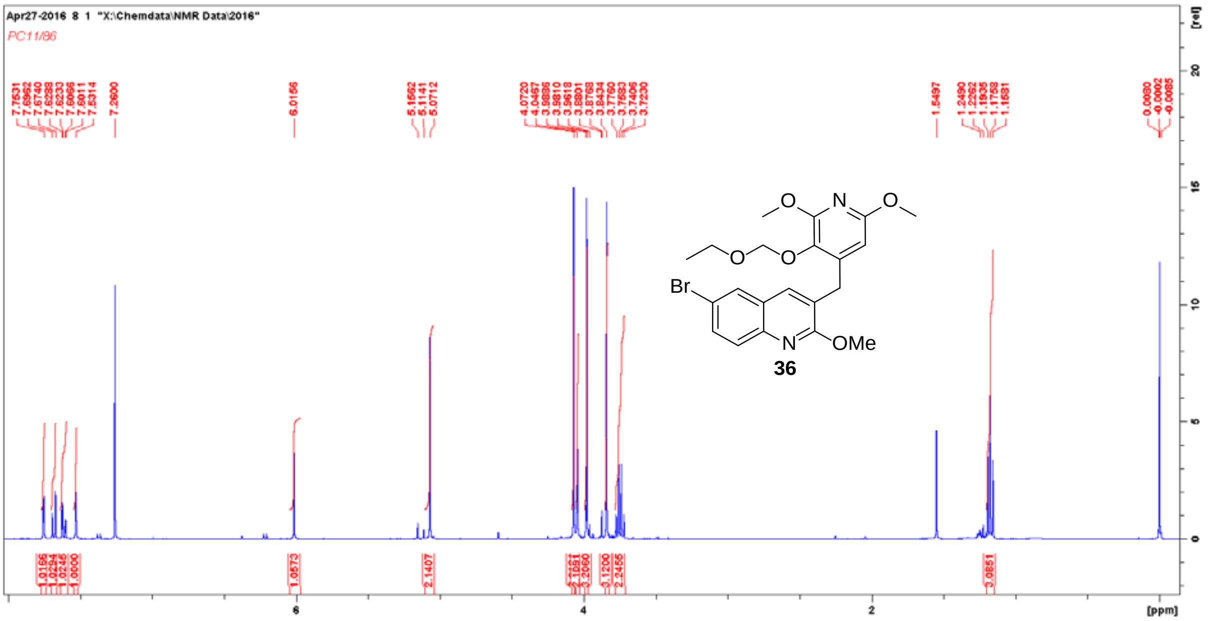

Figure 12: ^{1}H NMR of ***39***

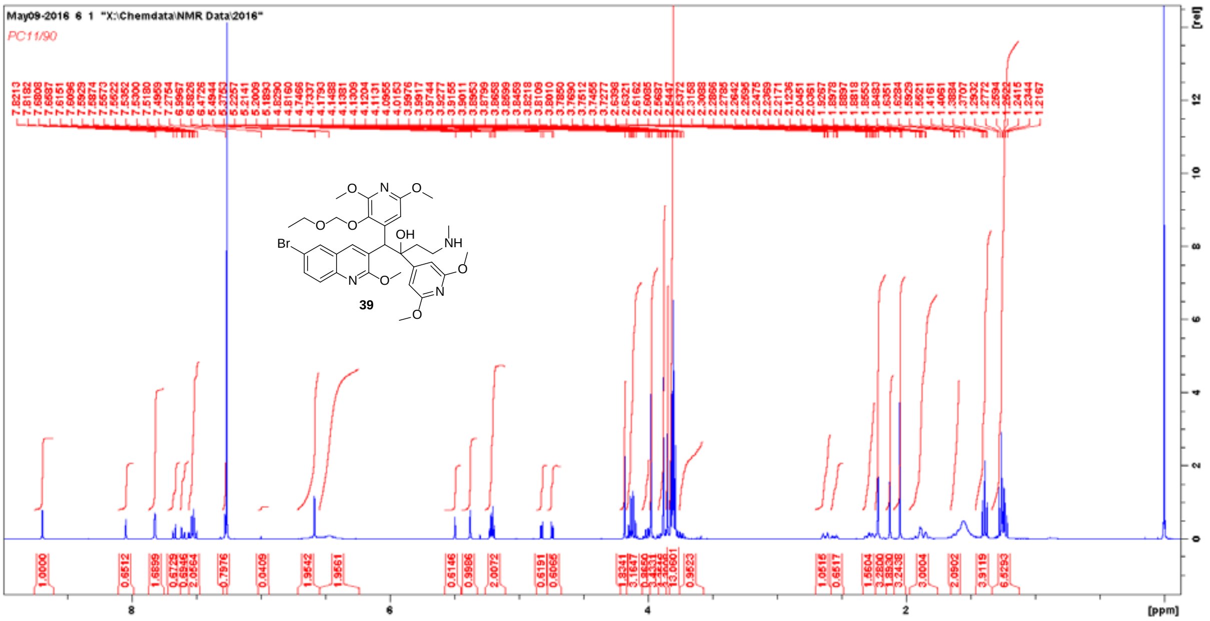

*Figure 13: ^{1}H NMR of **5A isomer A***

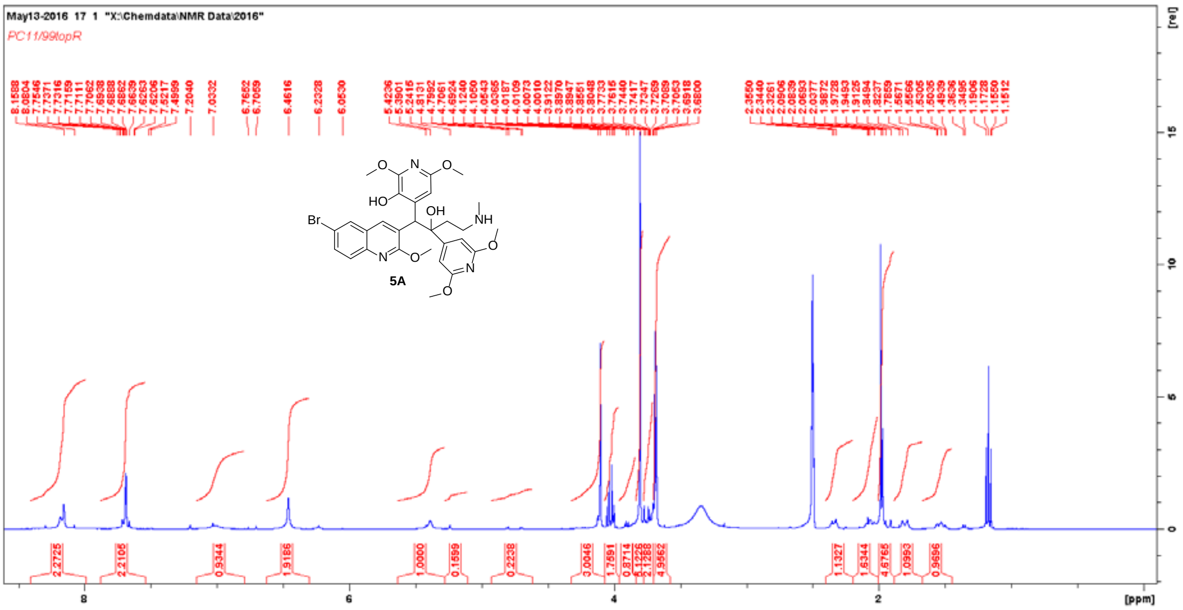

*Figure 14: ^{1}H NMR of **5A isomer B***

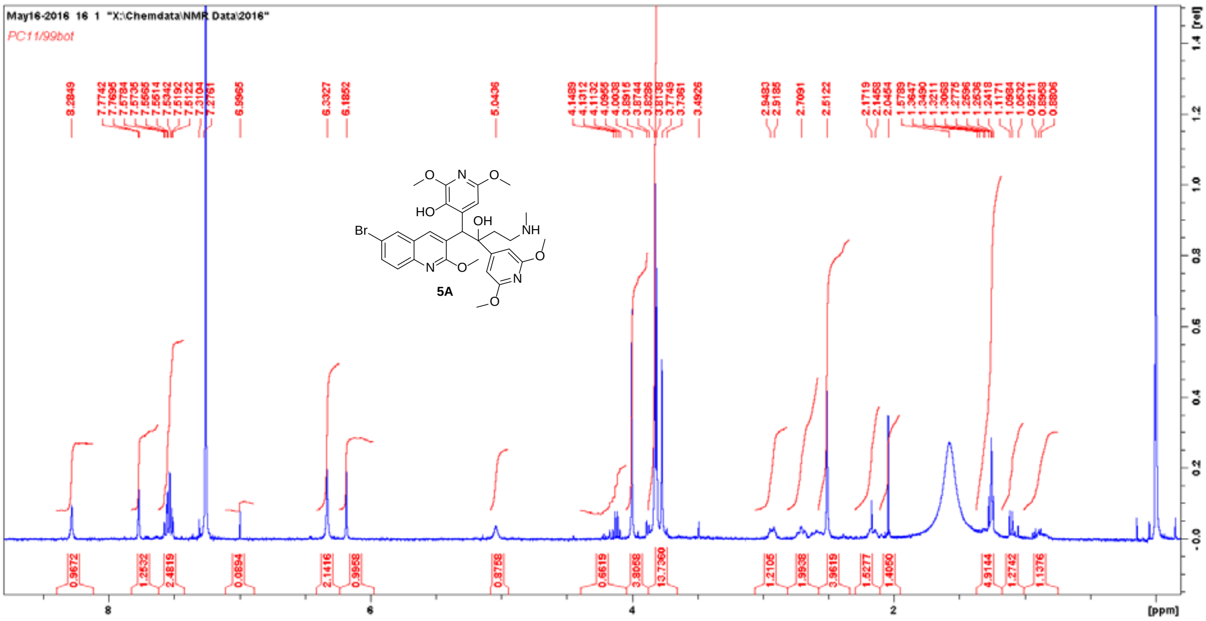

*Figure 15: ^{1}H NMR of **41***

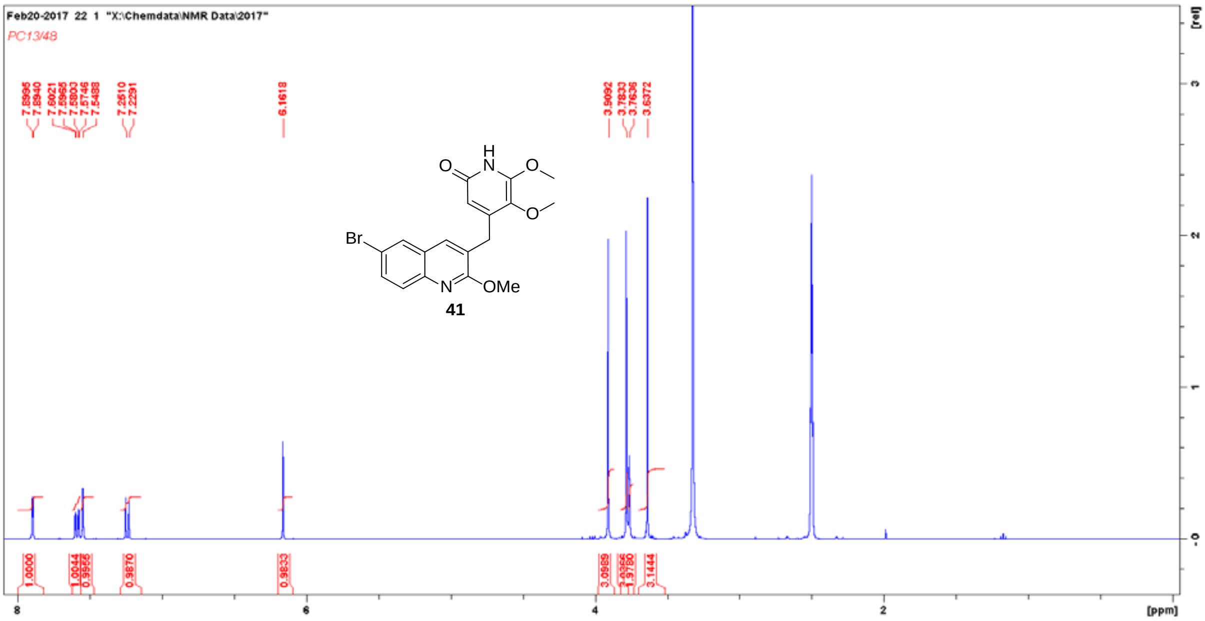

*Figure 16: ^{1}H NMR of **43***

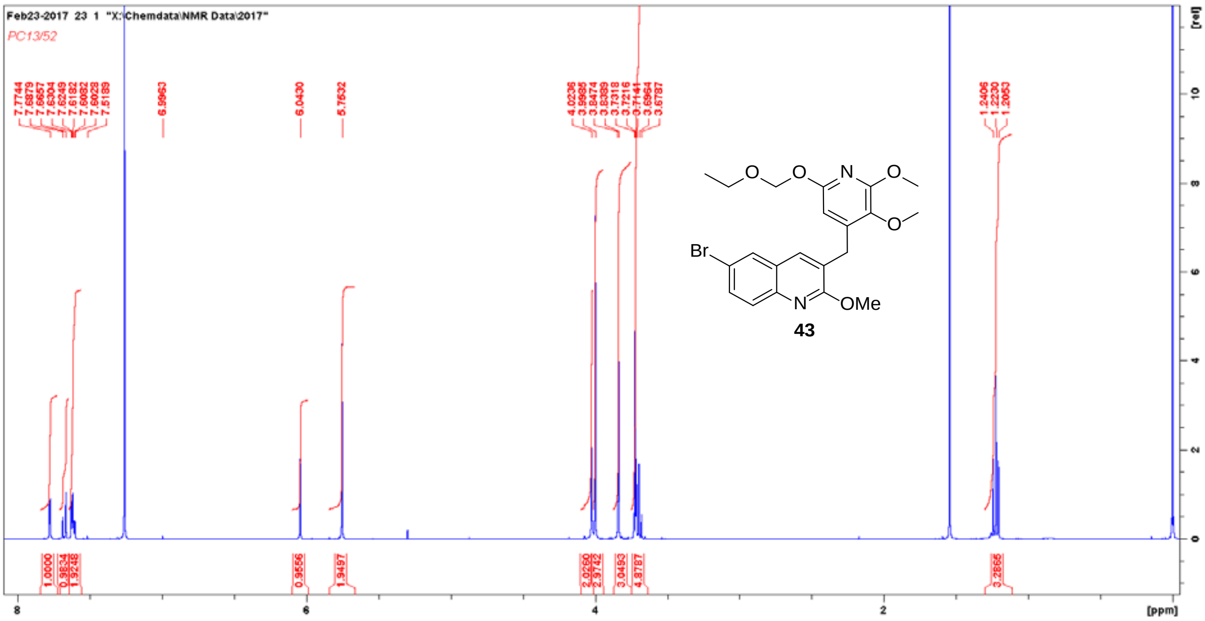

Figure 17: ^{1}H NMR of **5B isomer A**

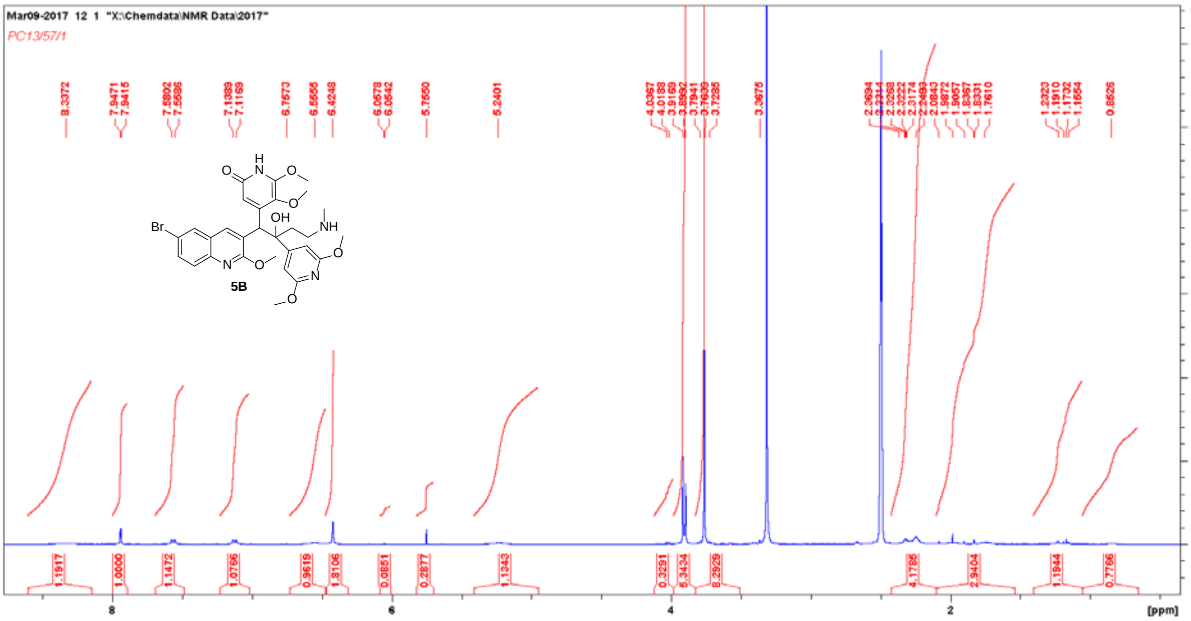

Figure 18: ^{1}H NMR of **5B isomer B**

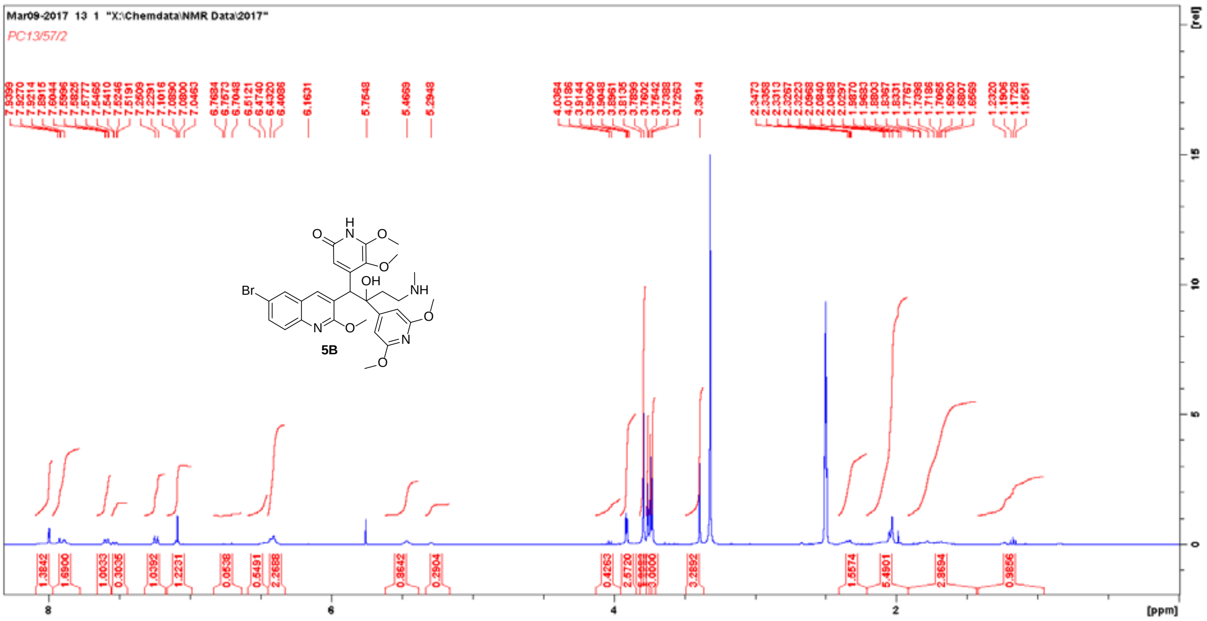

Figure 19: ^{1}H NMR of ***50***

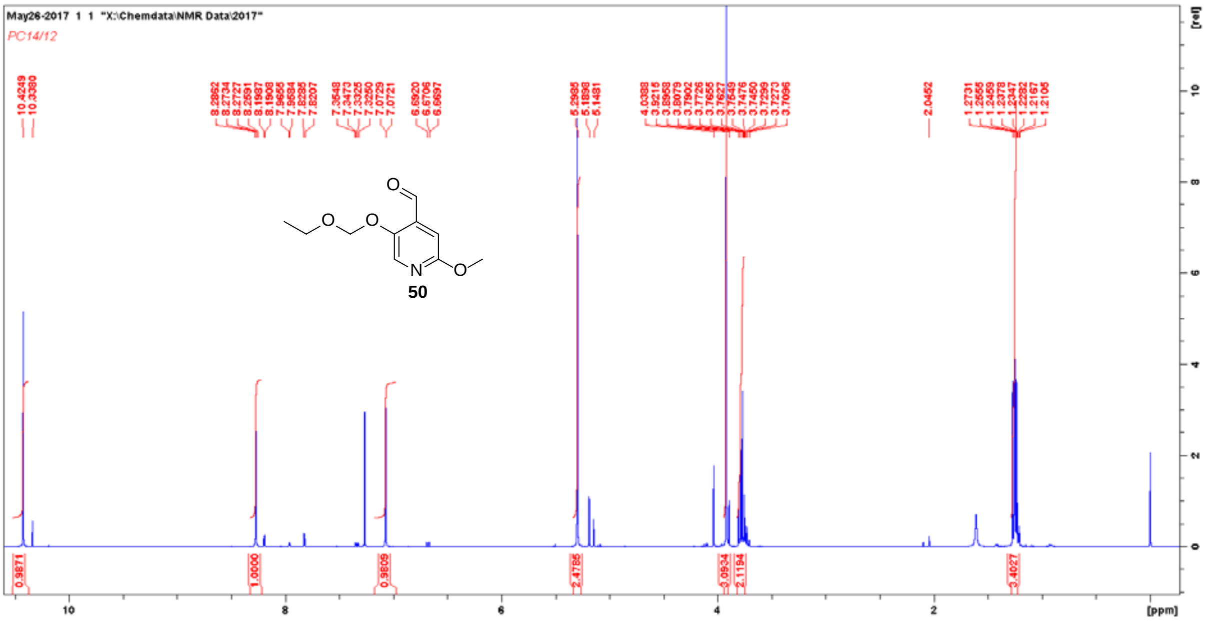

Figure 20: ^{1}H NMR of ***51***

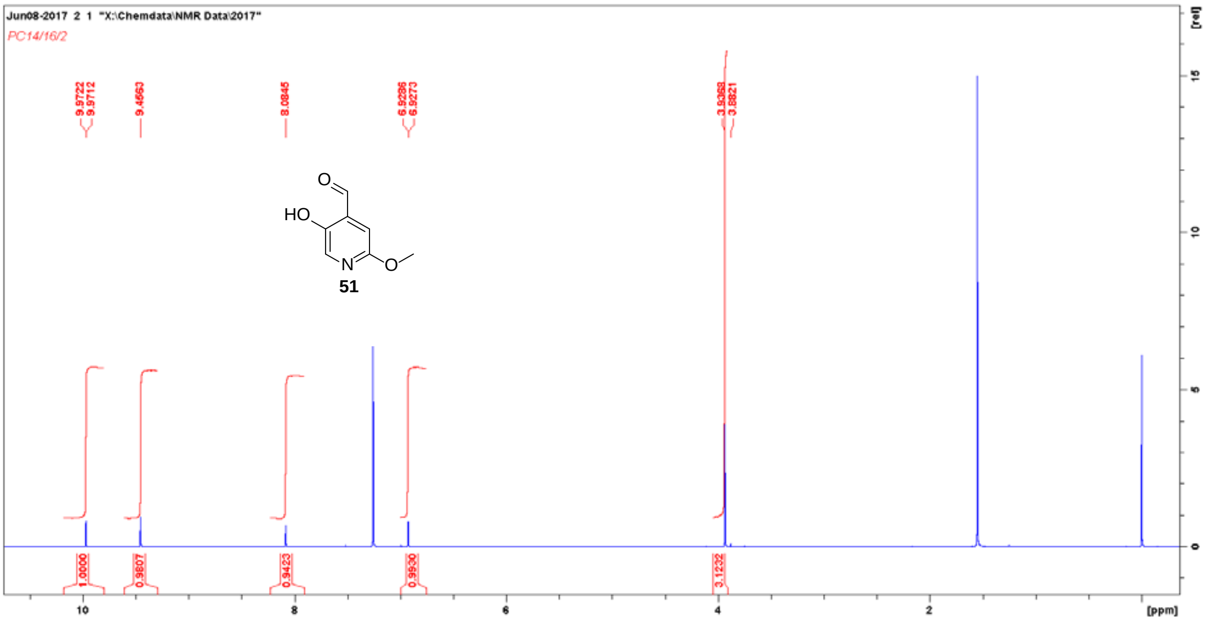

Figure 21: ¹H NMR of ***52***

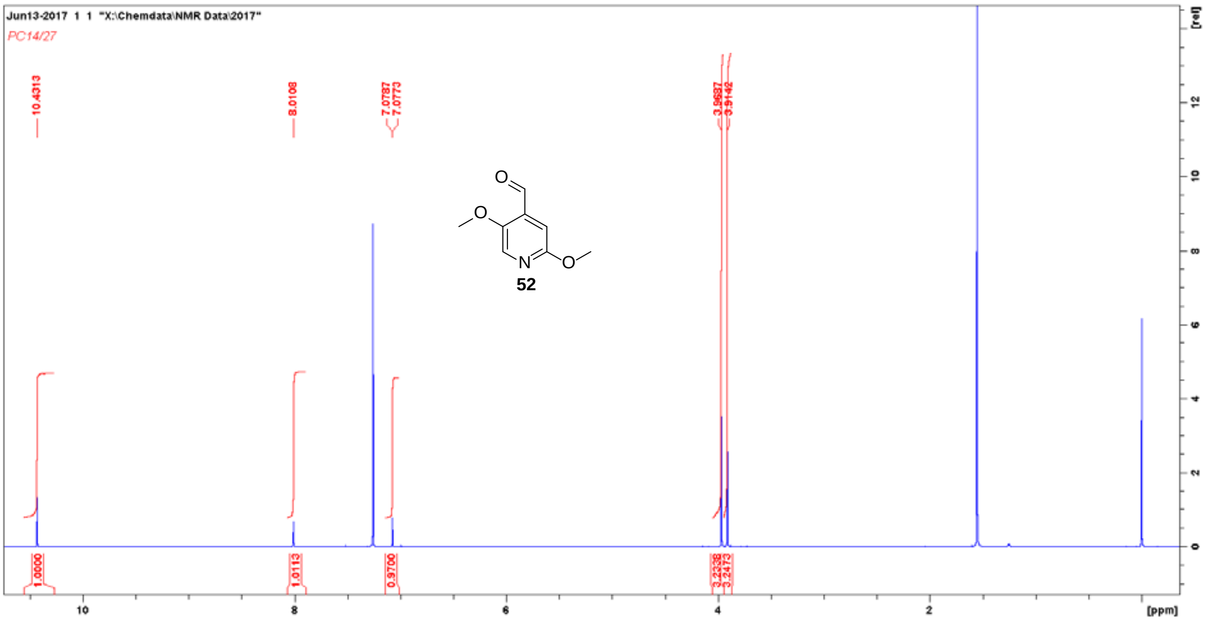

Figure 22: ¹H NMR of ***53***

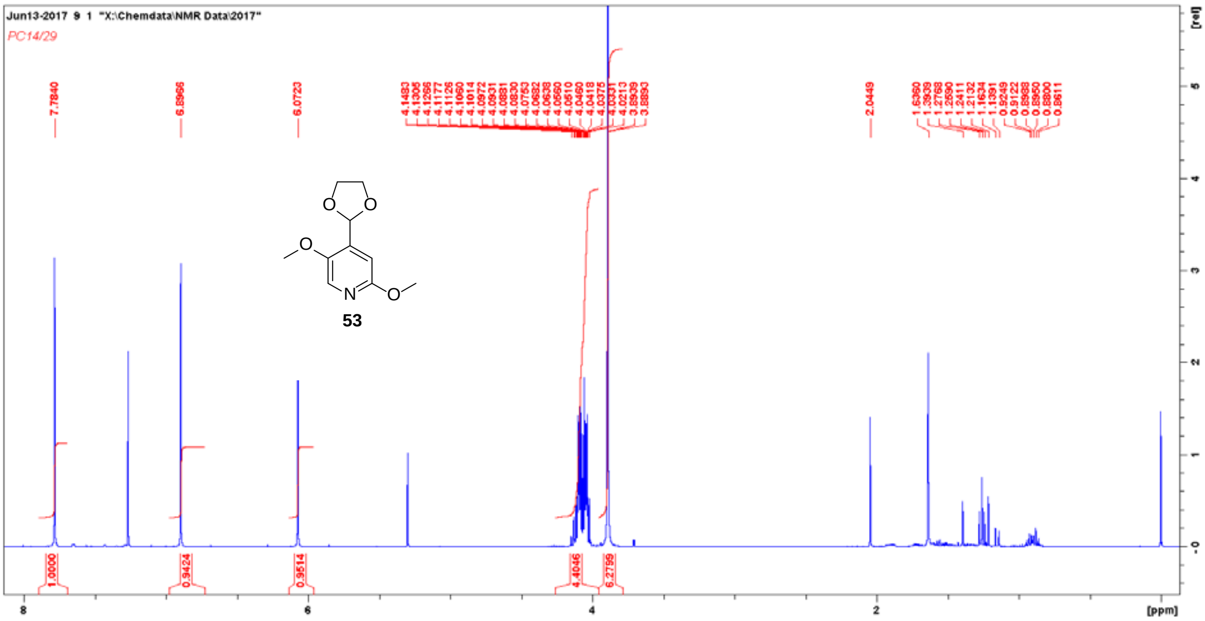

*Figure 23: ^{1}H NMR of **54***

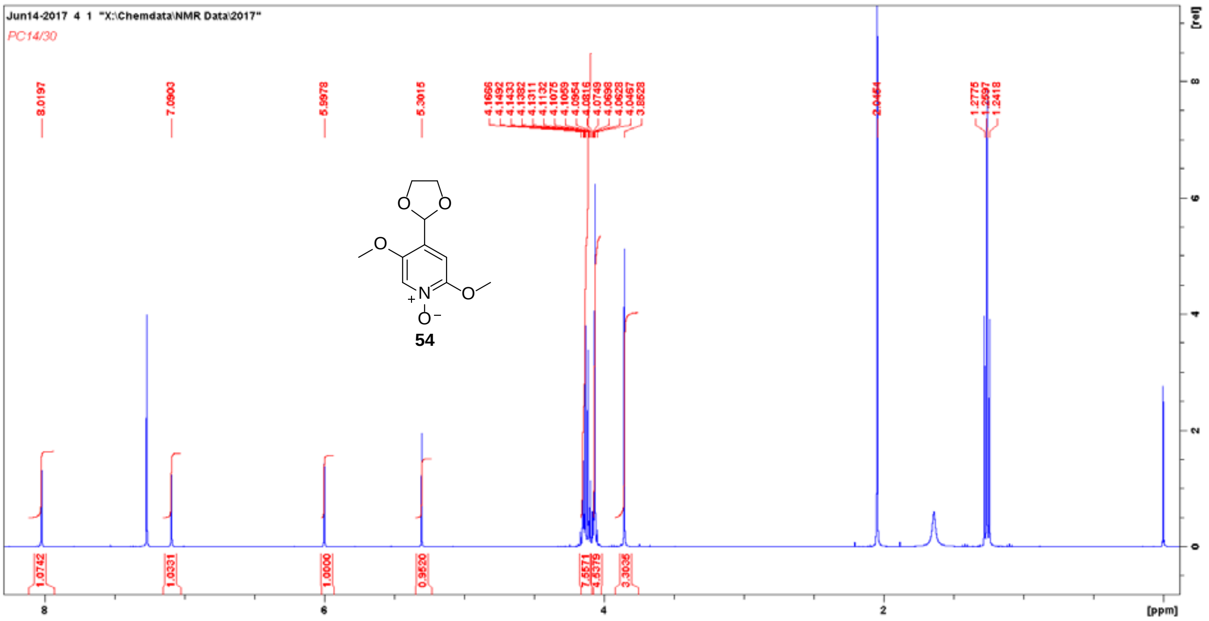

*Figure 24: ^{1}H NMR of **55***

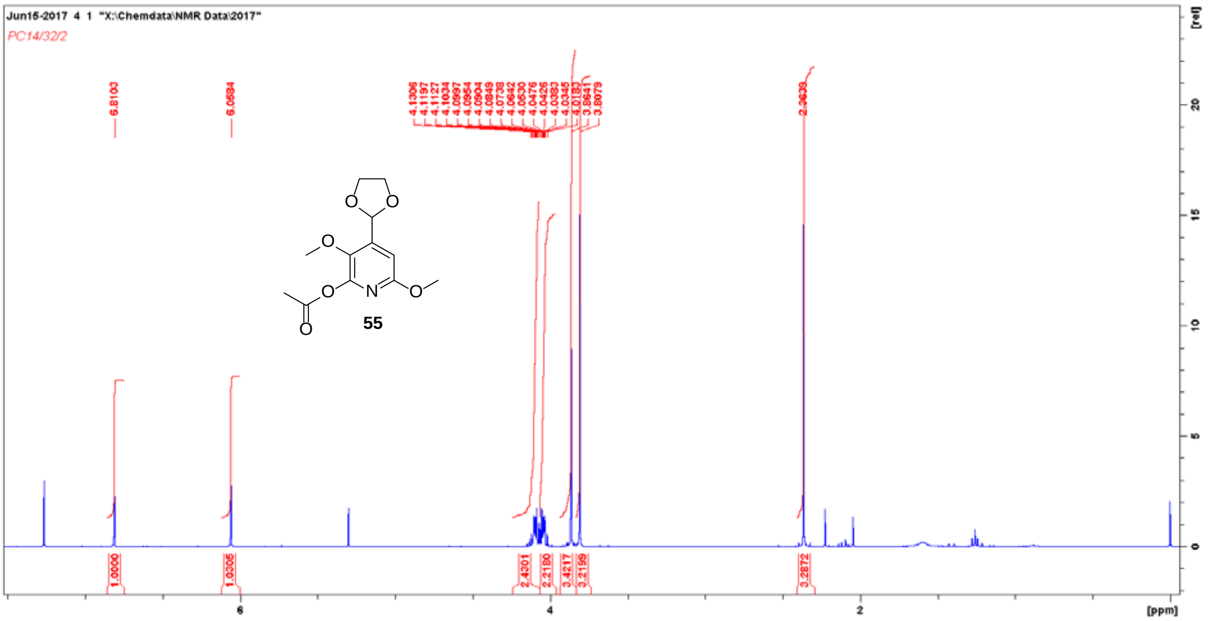

Figure 25: ^{1}H NMR of ***56***

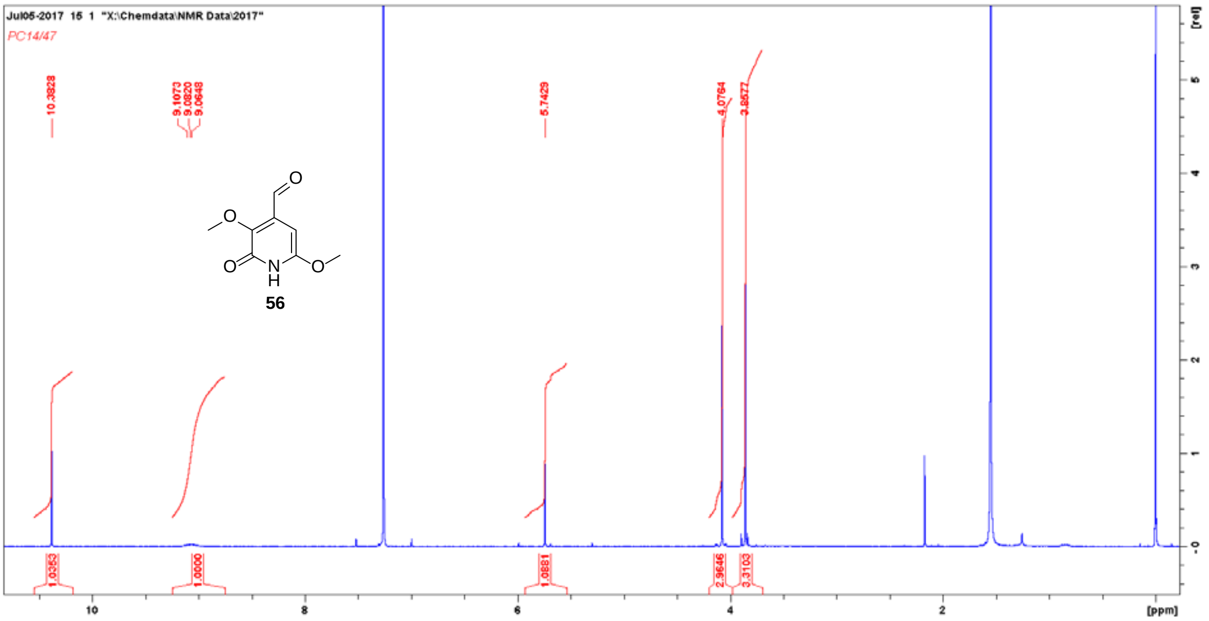

Figure 26: ^{1}H NMR of ***57***

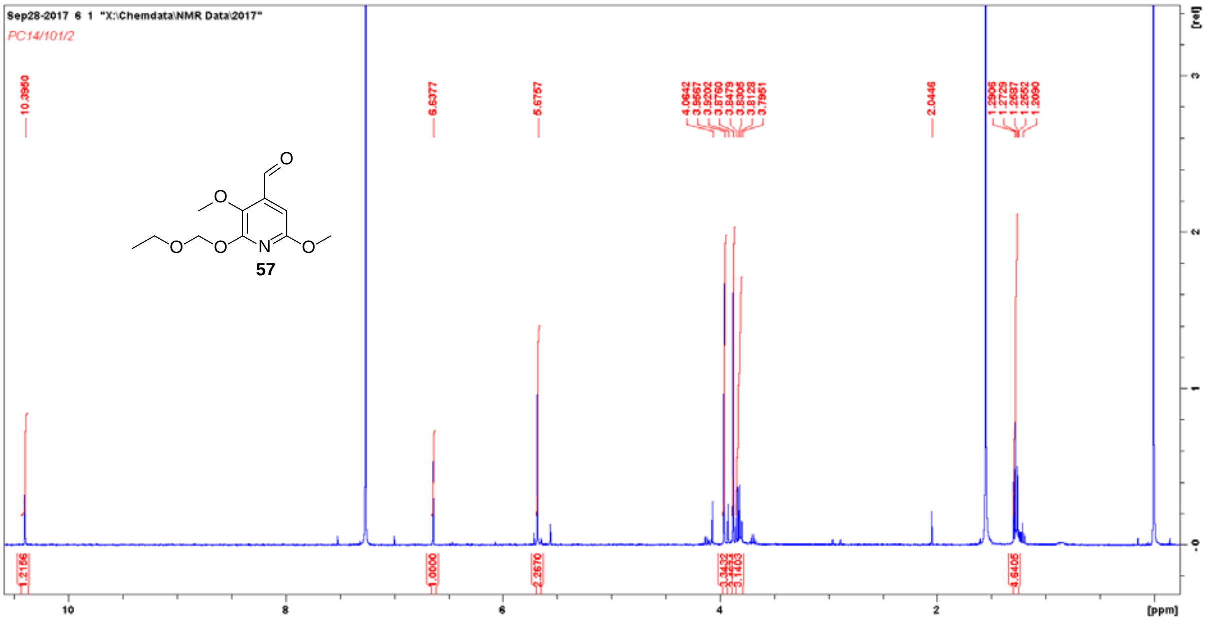

*Figure 27: ¹H NMR of **63***

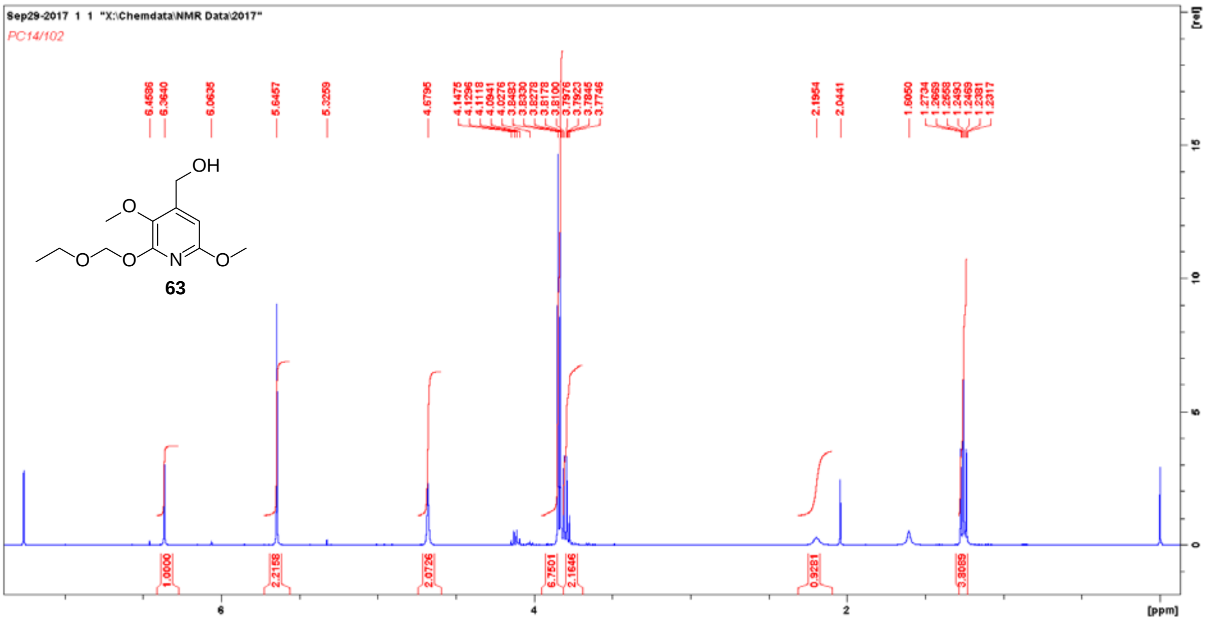

*Figure 28: ¹H NMR of **64***

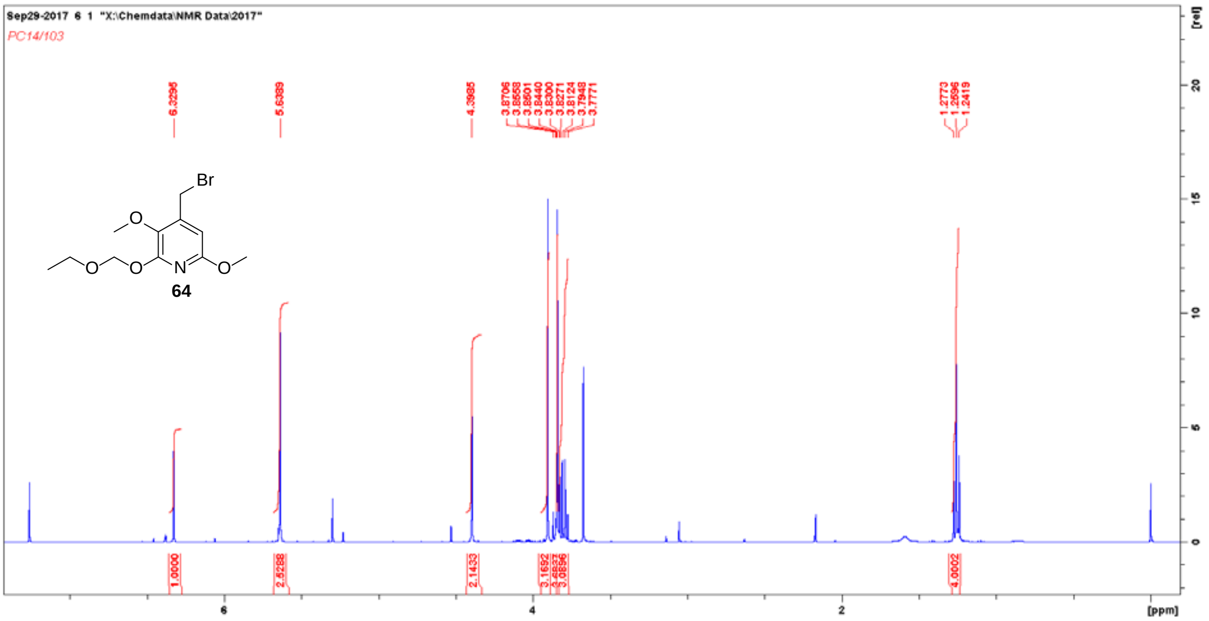

Figure 29: ^{1}H NMR of ***65***

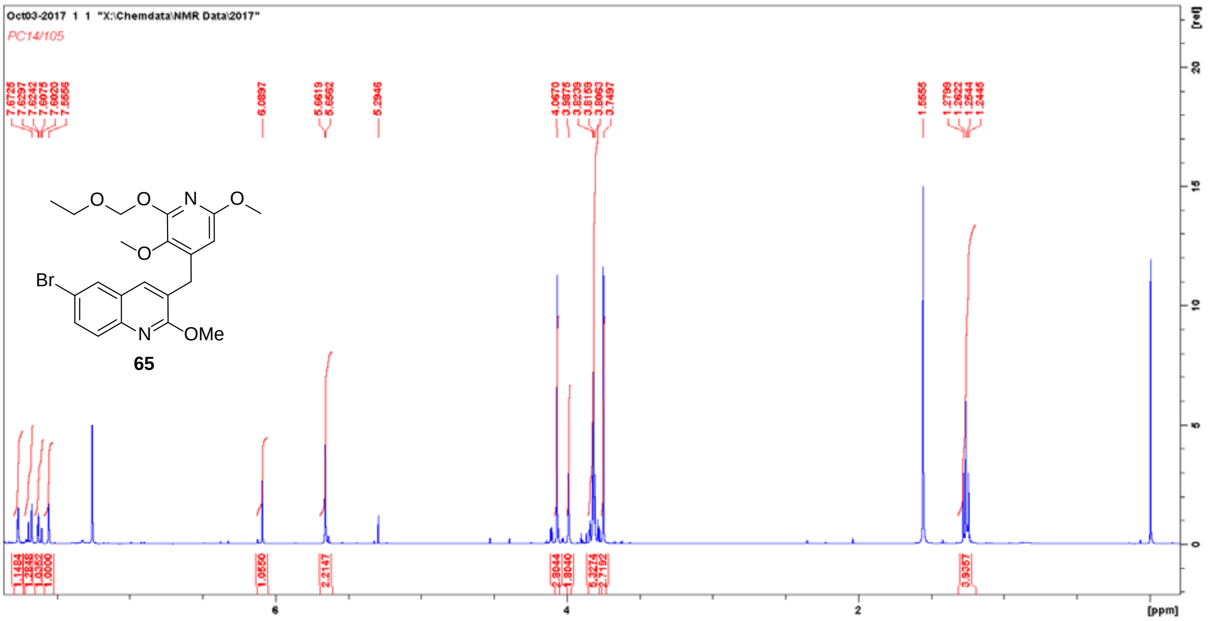

Figure 30: ^{1}H NMR of ***5C***

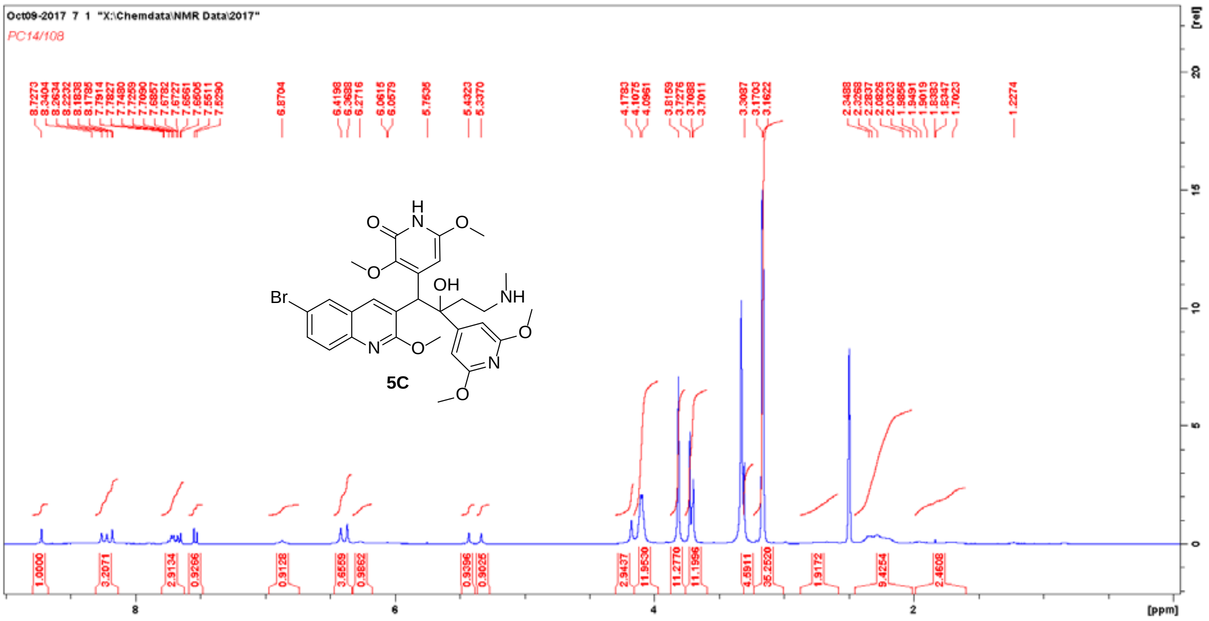

*Figure 31: ^{1}H NMR of **70***

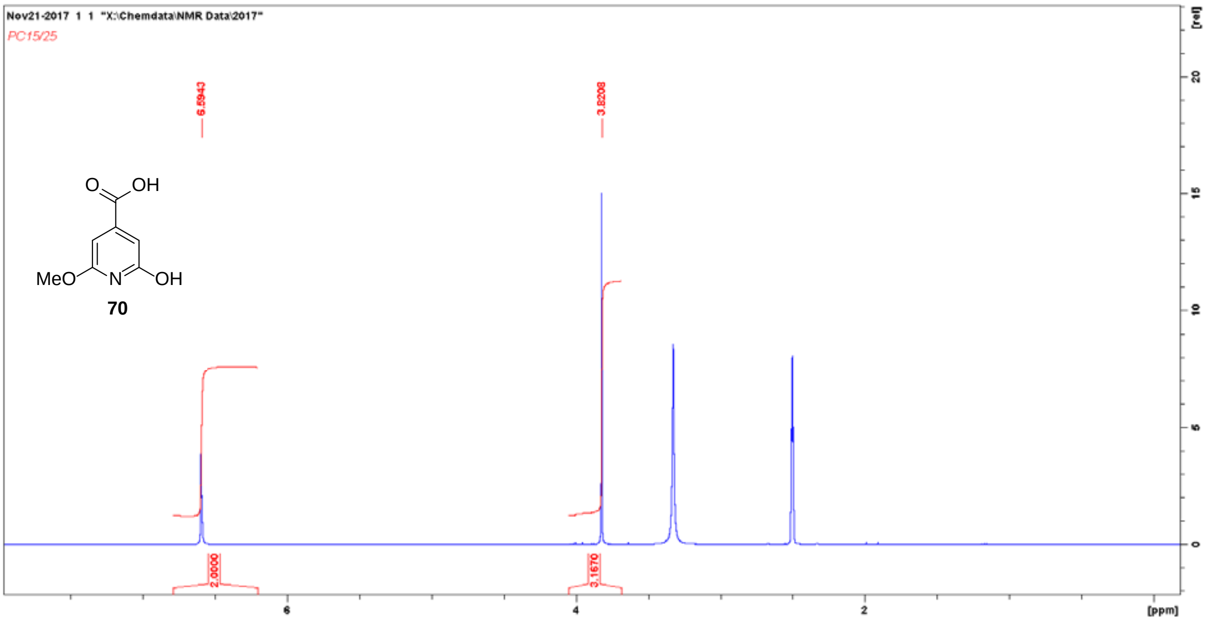

*Figure 32: ^{1}H NMR of **72***

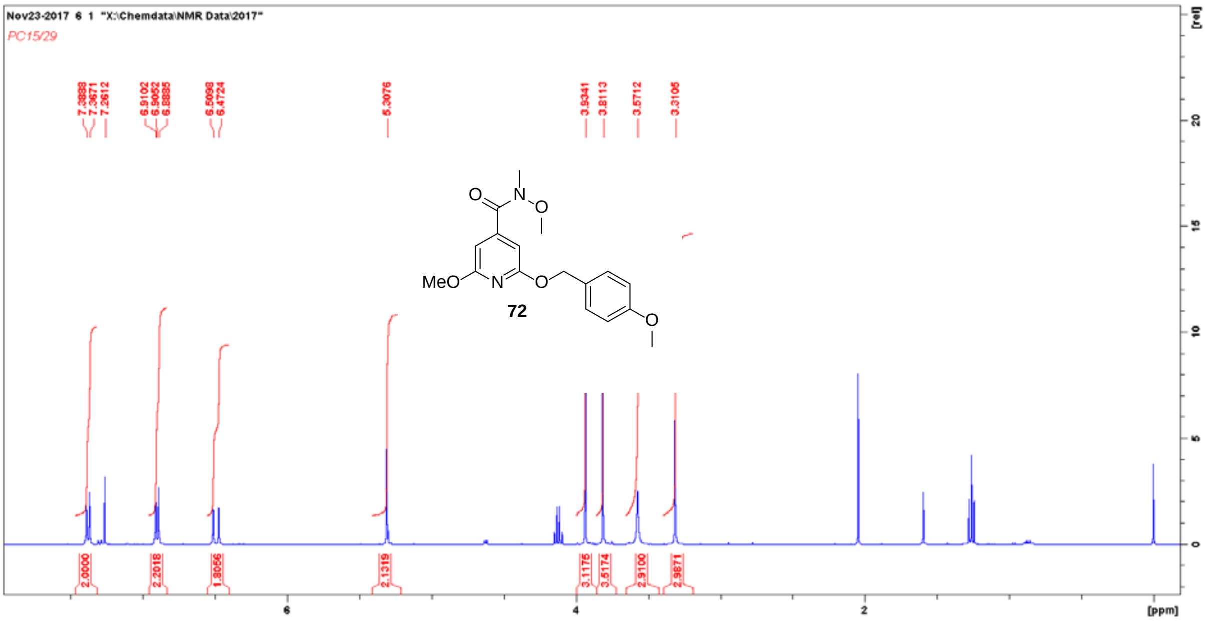

Figure 33: ^{1}H NMR of ***74***

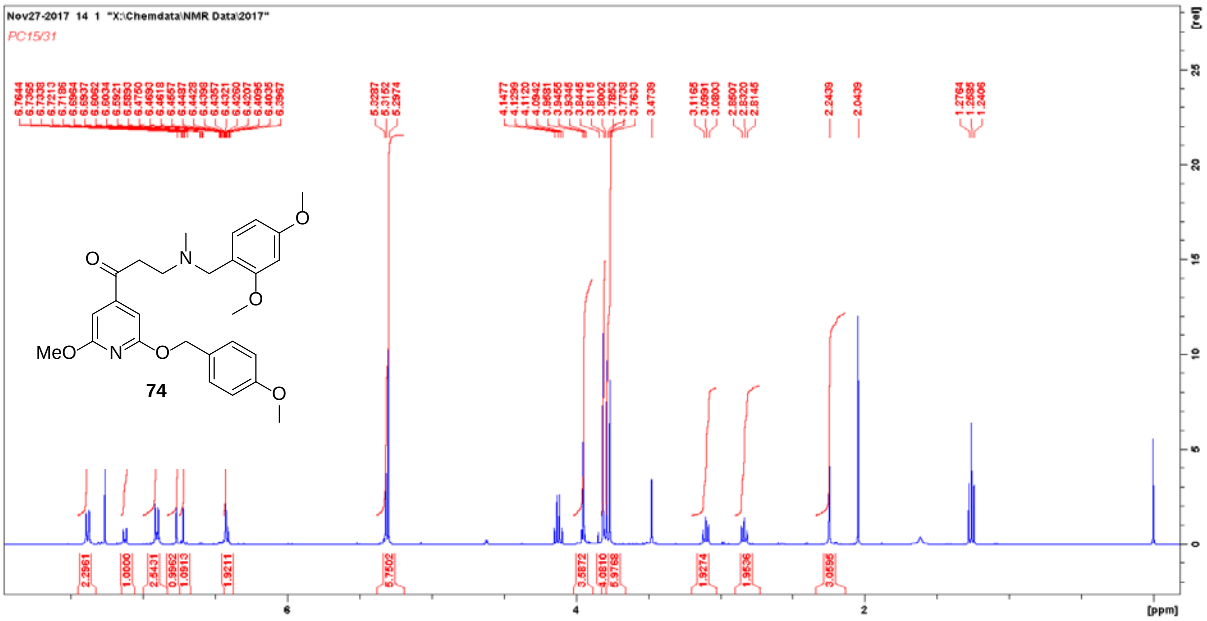

Figure 34: ^{1}H NMR of ***5D***

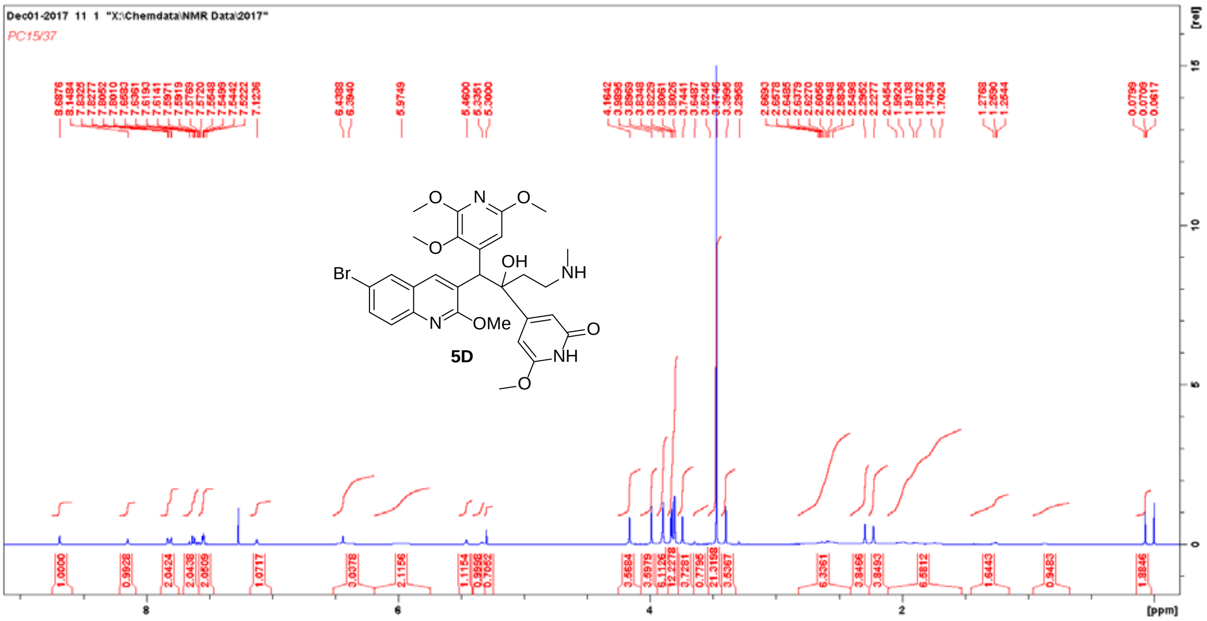

Figure 35: ^{1}H NMR of **81**

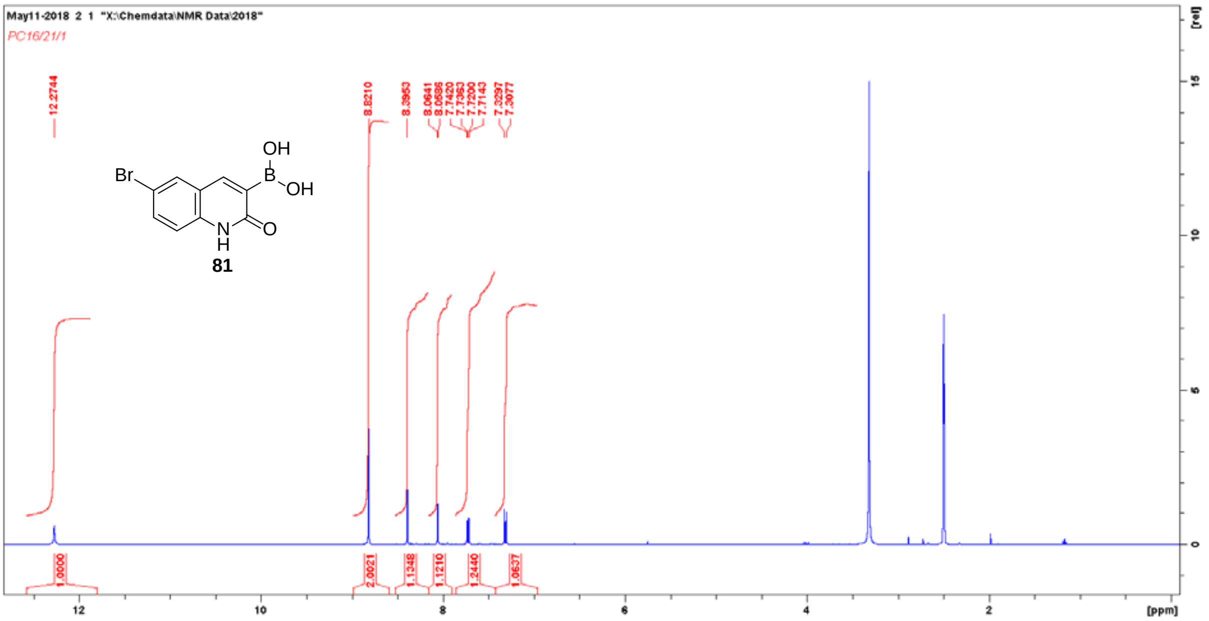

Figure 36: ^{1}H NMR of **82**

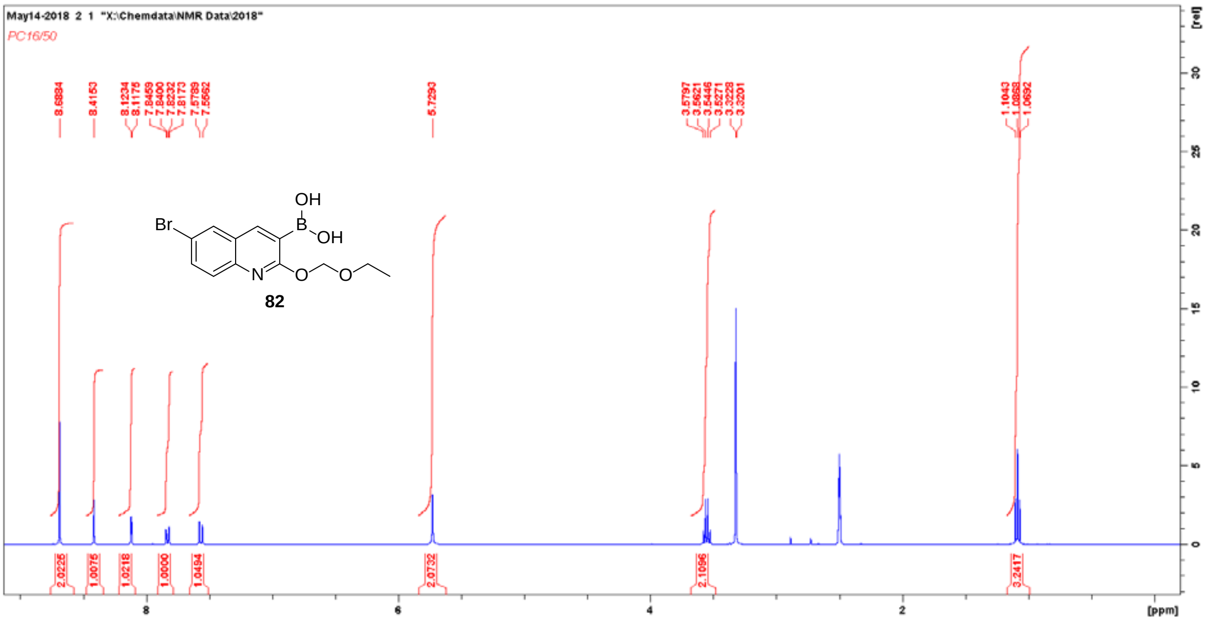

*Figure 37: ^{1}H NMR of **83***

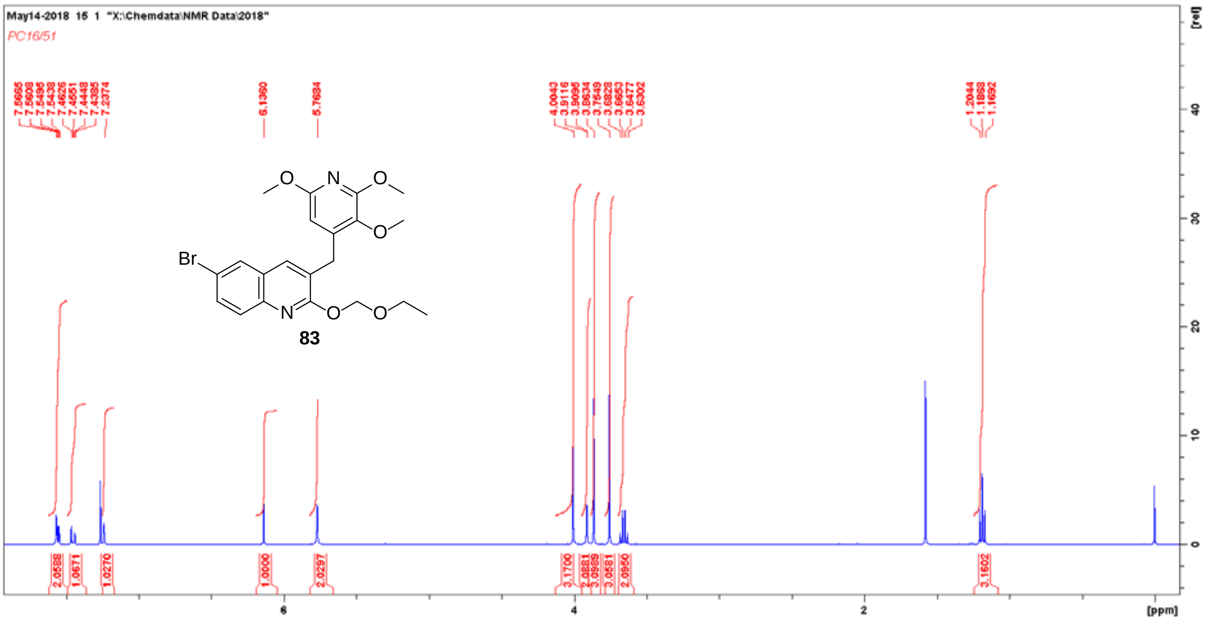

*Figure 38: ^{1}H NMR of **5E Isomer A***

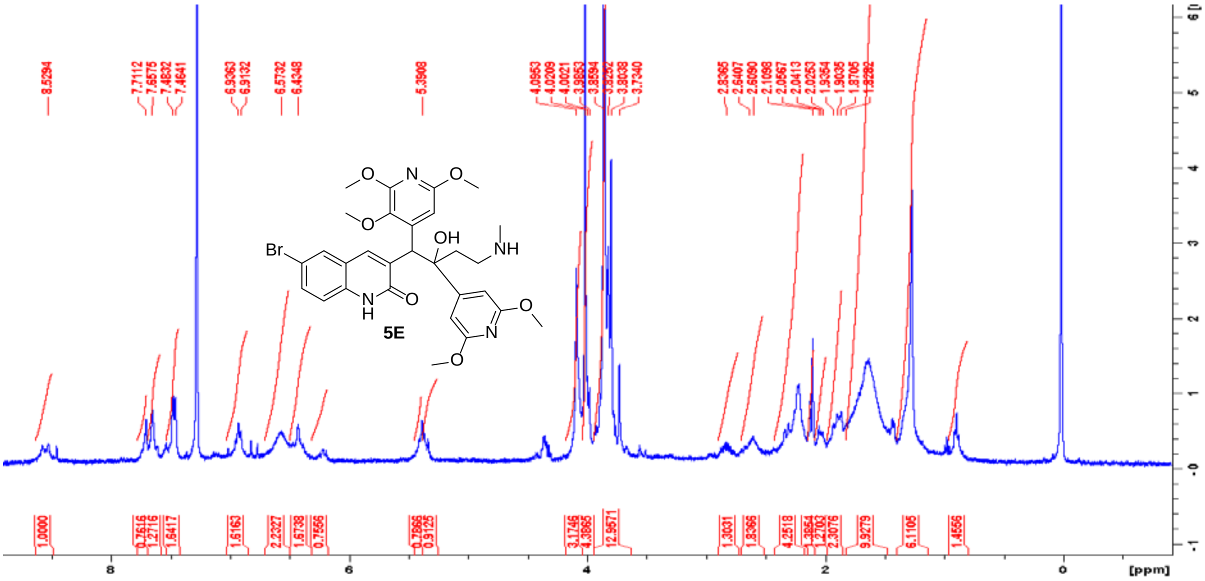

Figure 39: ^{1}H NMR of ***5E Isomer B***

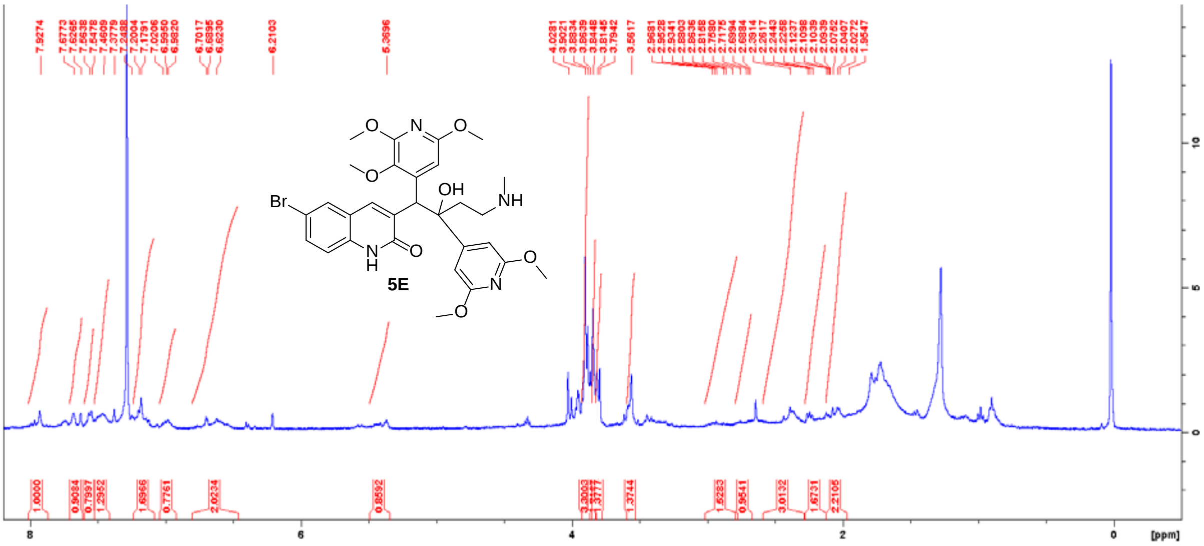

Supplement: Supplementary file 1 [file molecules-25-01423-s001.pdf]
